# On-DNA photochemical methods for expanding DNA-encoded library (DEL) chemical space

**DOI:** 10.1039/d6md00764c

**Published:** 2026-09-30

**Authors:** Oliver J. Corrigan, Sarah L. Allinson, Jamie H. Docherty

**Affiliations:** a Department of Chemistry, School of Science and Technology, Lancaster University LA1 4YB UK j.docherty2@lancaster.ac.uk; b Division of Biomedical and Life Sciences, School of Health and Medicine, Lancaster University LA1 4YB UK s.allinson@lancaster.ac.uk

## Abstract

DNA-encoded libraries (DELs) have become a mainstay of small-molecule discovery, enabling millions to billions of compounds to be screened in a single pooled affinity selection in which each small molecule is covalently linked to a unique DNA identifier. However, the chemistry available for their construction remains limited compared to conventional synthetic chemistry. On-DNA chemistry is constrained by the requirement for aqueous, dilute, and DNA-compatible conditions. Classical on-DNA reactions such as amide coupling, reductive amination, S_N_Ar and transition-metal cross-coupling have enabled reliable library synthesis, but they tend to favour planar, sp^2^-rich products. Photochemistry and photocatalysis have recently emerged as a powerful strategy to broaden this toolbox. Excited-state activation opens reaction pathways that are difficult or impossible to reach through conventional ground-state chemistry and can facilitate novel transformations towards the construction of more complex and sp^3^-rich scaffolds. This review surveys the field of on-DNA photochemistry, looking at different activation modes including photoredox single-electron transfer (SET), hydrogen-atom transfer (HAT), metallaphotoredox dual catalysis, electron donor–acceptor (EDA) complexes, energy-transfer (EnT) catalysis, and direct photolysis. Collectively, these methods have translated a broad range of useful transformations to be amenable to on-DNA synthesis and unlocked access to many medicinally valuable motifs.

## Abstract 2

On-DNA photochemistry and photocatalysis enable the synthesis of diverse structures for DNA-encoded libraries, expanding the chemical space available for DEL screening and the discovery of active compounds.

## Graphical abstract

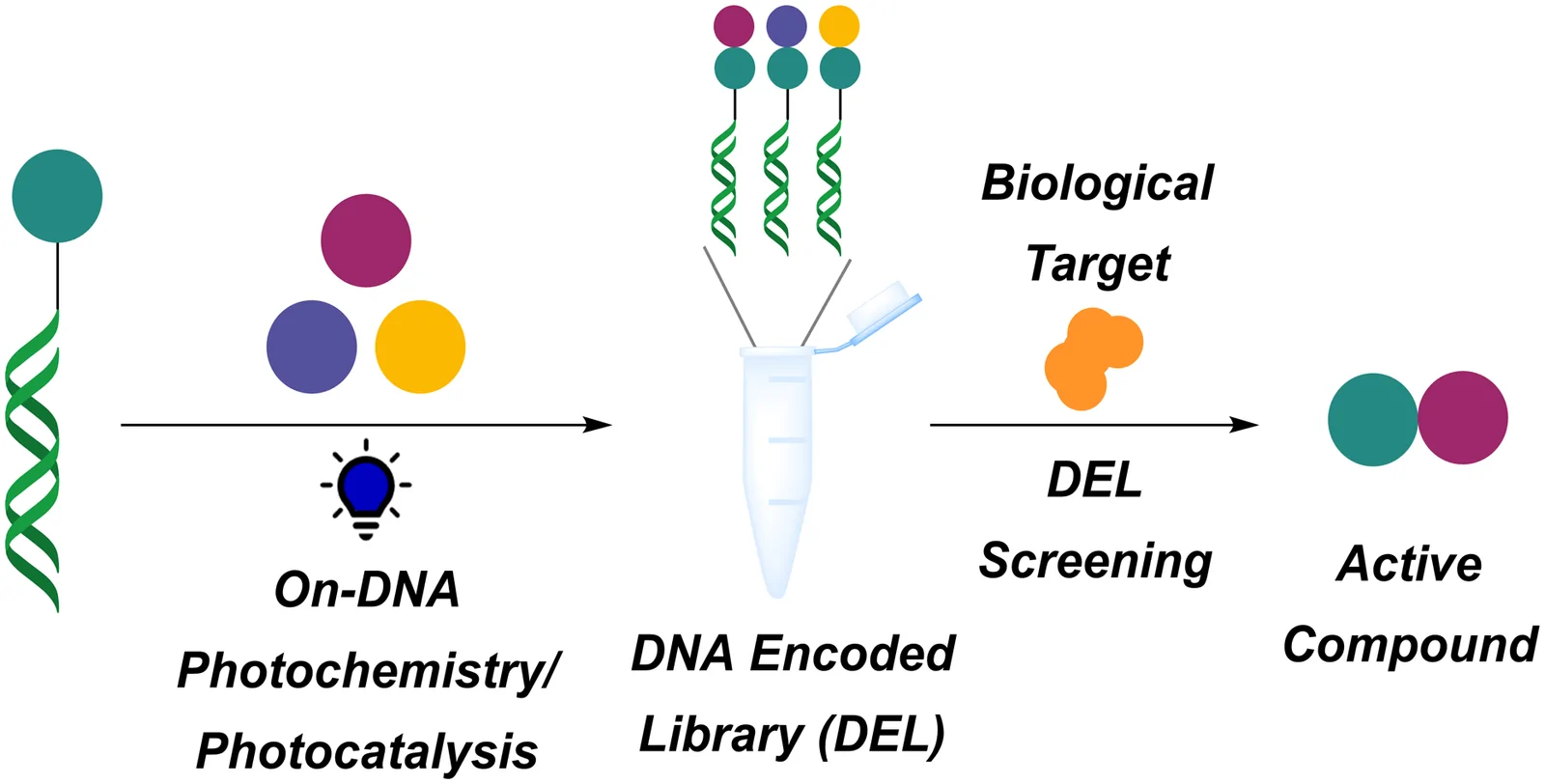

## 1. Introduction

### 1.1 DNA-encoded libraries

DNA-encoded libraries (DELs) present a powerful technology in drug discovery, enabling the rapid and cost-effective screening of small molecules against biological targets. In a DEL, each small molecule is covalently linked to a DNA sequence that records its synthetic history and therefore encodes its chemical identity. This link between DNA sequence and chemical identity allows millions to billions of compounds to be screened together in a single pooled affinity-selection experiment, rather than as individually synthesised and purified compounds.^1–3^ When a DEL is exposed to a target protein, molecules that bind to the target are enriched through affinity selection while the non-binding molecules are washed away. Subsequently, the DNA tags conjugated to each compound are amplified using the polymerase chain reaction (PCR) and sequenced using high-throughput methods. This sequencing allows for the DNA ‘barcode’ to be ‘read’ and helps identify the specific chemical structures that interacted with the target (Scheme 1A).^1,2^

**Scheme 1:**
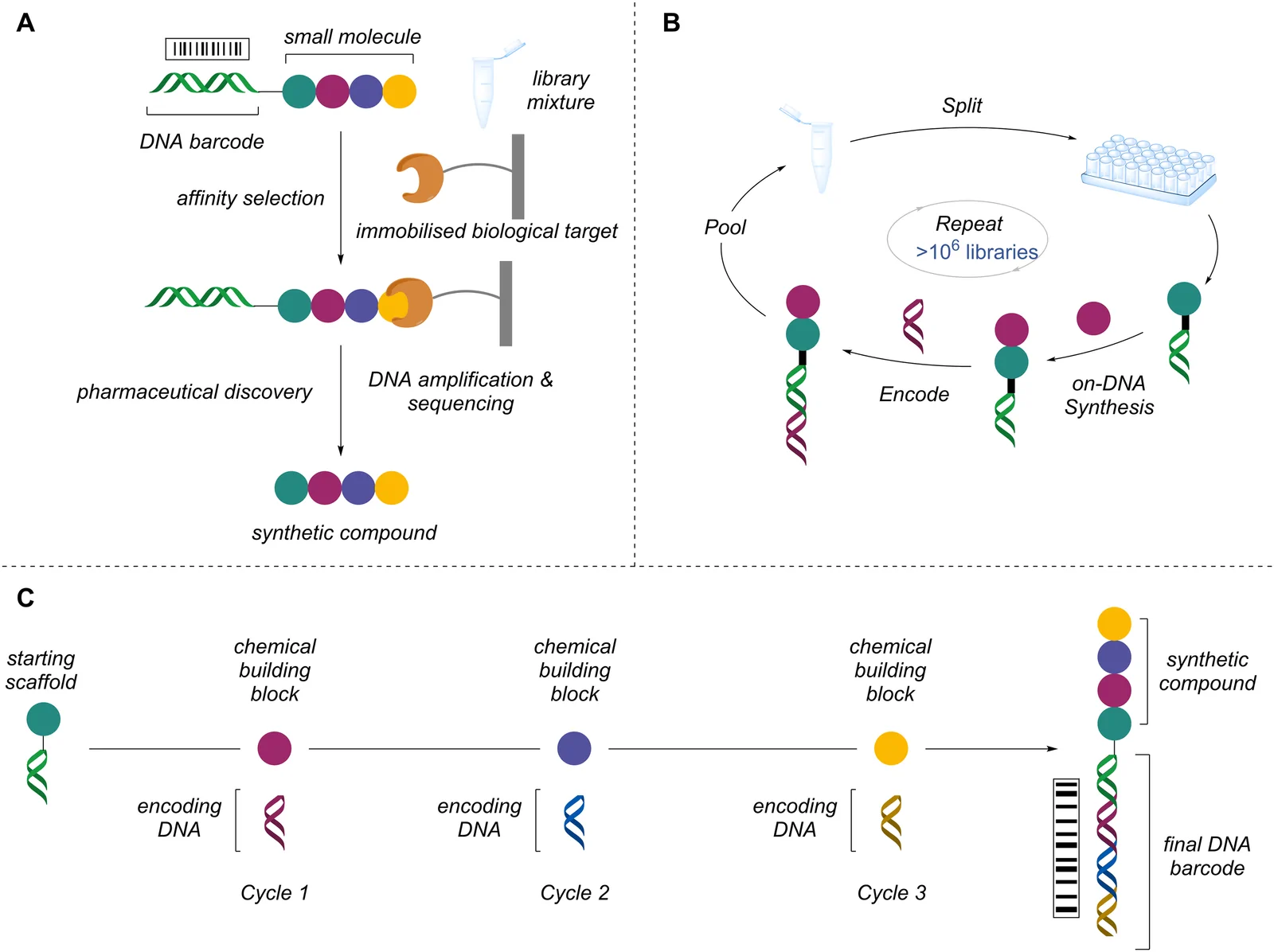
(A) DEL screening: A library of DNA-encoded, drug-like compounds is screened against a biological target. Compounds that successfully bind are retained, their DNA tags are amplified and sequenced to reveal the hit compound. (B) Split and pool synthesis: A DNA-conjugated chemical fragment is split into a multi-well plate. To each well, a unique chemical building block is added followed by a unique encoding strand of DNA. The products are then pooled before another round of synthesis. (C) DEL construction: DELs are typically constructed in three cycles from a starting scaffold. In each cycle, a series of building blocks is coupled followed by a unique DNA tag for each building block.

The idea that synthetic molecules could be tracked by DNA was first articulated in 1992 by Brenner and Lerner, who envisioned the construction of a library of polypeptides each conjugated to a unique DNA identifier.^4,5^ Although initially limited by the cost and labour of DNA sequencing, as these processes became more routine, the concept of DNA-encoded libraries was revisited and eventually made its way into modern drug discovery. As proposed in the seminal 1992 paper, DNA-encoded libraries exploit the novel use of combinatorial chemistry to rapidly generate large libraries. DNA-conjugated chemical scaffolds can be split into multi-well plates where they can subsequently undergo on-DNA chemical reactions with chemical building blocks, followed by encoding with a unique DNA tag (Scheme 1B). The addition of a unique building block to each well allows for rapid diversification of the scaffold. Following on-DNA synthesis and encoding the DNA-conjugated functionalised products are pooled and split into wells once again for another round of synthesis at an orthogonal functional handle. This split-and-pool process can be repeated across successive rounds of synthesis, with each chemical step paired to an encoding step so that the final DNA barcode records the full synthetic history of each library member. In many DEL formats, library construction proceeds through two to three encoded synthetic cycles, with three-cycle libraries representing a common design (Scheme 1C). This format provides powerful multiplicative growth. For example, the incorporation of 100 building blocks in each of three cycles would generate a theoretical library of 10^6^ uniquely encoded compounds. Additional cycles can further increase library size, but also increase the risk of incomplete conversion, truncated sequences, accumulated impurities and high-molecular-weight products with less desirable drug-like properties. DEL design therefore involves a balance between numerical scale and chemical quality. The most useful libraries are not simply the largest, but those in which each encoded cycle introduces meaningful structural diversity while preserving DNA integrity, synthetic fidelity and downstream sequencing potential.

In an industrial context, DEL hits typically provide an initial active compound or scaffold that undergoes several rounds of further optimisation to give a candidate for clinical trials. It is rarely the initial DEL hit itself that makes it to this stage. It is difficult to quantify the success of DEL screening in producing clinical candidates due to the confidential nature of this data. However, over the past decade there have been growing reports in the literature of DEL-derived compounds with potent biological activity (Scheme 2). In 2013 there were already several reports of bioactive compounds derived from DELs which showed promising *in vitro* activity.^6–8^ However, in this year, GlaxoSmithKline reported the first DEL-derived clinical candidate: the selective sEH (soluble epoxide hydrolase) inhibitor GSK2256294.^9,10^ The initial hit for this compound was discovered from an affinity selection of two 1,3,5-triazine-based DELs which had a combined library size of around 9 × 10^8^ compounds.^11^ Phase I studies in healthy volunteers and COPD patients showed a long half-life and good tolerability.^12^ Following this, two phase IIa trials were conducted: one in aneurysmal subarachnoid haemorrhage and another in obese/pre-diabetic subjects. Treated patients showed target engagement and trends toward clinical benefit.^13–15^ This validated that DEL hits could be optimised into drug-like compounds with favourable pharmacokinetics and safety.

**Scheme 2:**
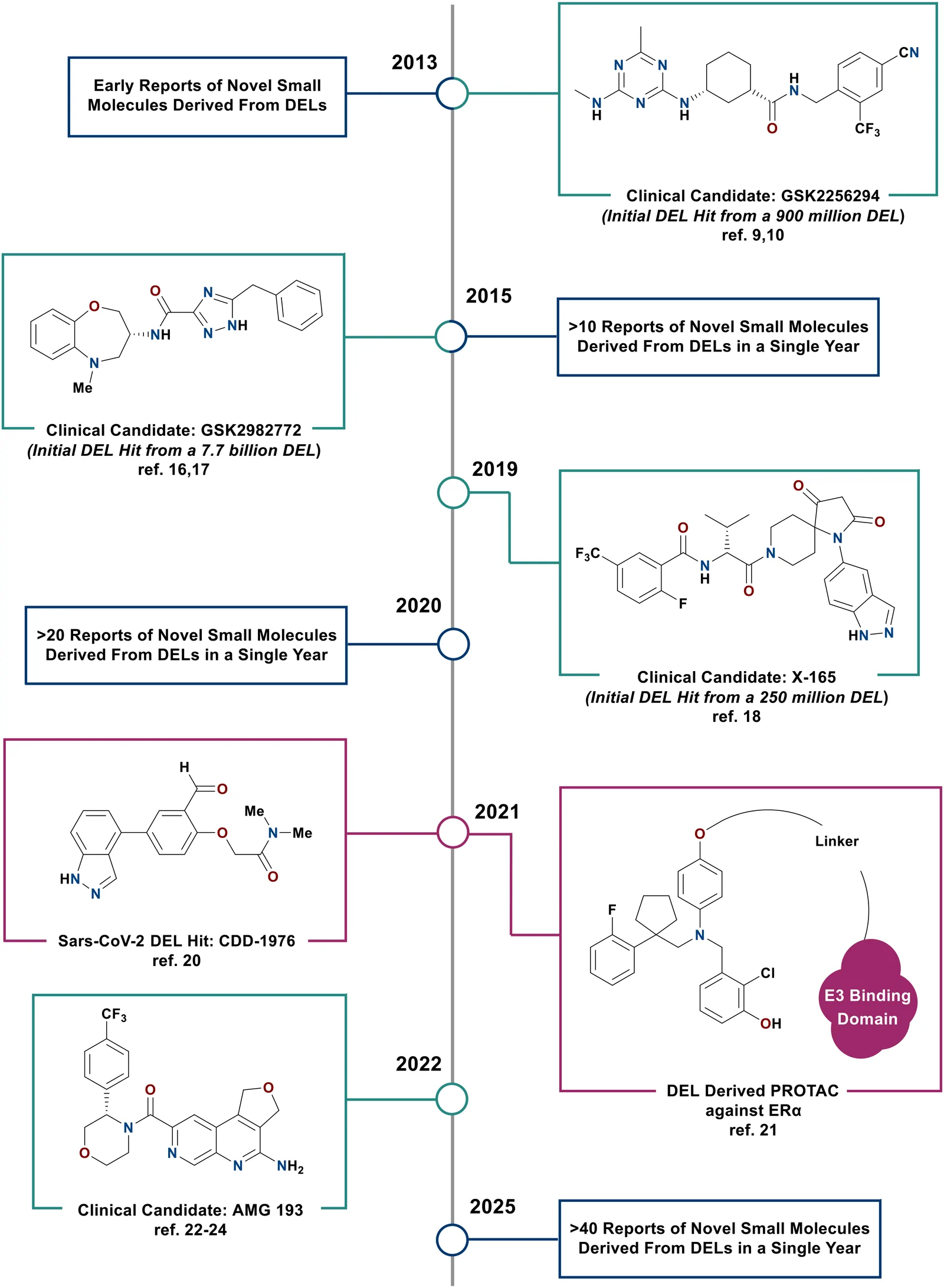
Timeline of DEL breakthroughs and clinical candidates. The first clinical candidate from a DEL was the sEH inhibitor GSK2256294 in 2013 followed by RIP1 kinase inhibitor GSK2982772 in 2015 and autotaxin (ATX) inhibitor X-165 in 2019. In 2021 DELs were used to try and find a SARS-CoV-2 treatment with CDD-1976 and were also utilised in PROTAC discovery for the first time. In 2022 DEL-derived PRMT5 inhibitor AMG 193 entered clinical trials.

Following on from this, DEL screening grew as an early-stage modality and by 2015 there were approximately 10 publications per year detailing novel, DEL-derived compounds. GSK's DEL platform continued to be a success with GSK2982772, a novel RIPK1 inhibitor also entering clinical trials in 2015. The original hit which led to the development of this candidate was discovered as a result of screening a 7.7 billion DEL library.^16^ Medicinal chemistry optimisation led to GSK2982772, a first-in-class clinical candidate with high kinase specificity. Subsequent clinical studies of GSK2982772 showed acceptable tolerability and pharmacological engagement but limited clinical efficacy across the inflammatory disease indications tested.^17^ This highlights that DEL screening successfully delivered a potent, selective and clinically testable RIPK1 inhibitor, while further progression depended on exposure, disease biology and demonstrable therapeutic benefit in patients.^13,17^

Another key clinical candidate derived from a DEL screen is the autotaxin (ATX) inhibitor, X-165. The initial DEL screen against ATX included over 200 million DNA-tagged molecules from which a hit was found to bind the ATX enzyme's substrate pocket in a novel manner.^13^ After hit optimization, X-165 emerged with low-nanomolar ATX inhibition, excellent oral bioavailability, and efficacy in preclinical IPF models.^18^ In early 2019, the FDA cleared the Investigational New Drug Application for X-165, enabling progression into phase I clinical testing and making it the first DEL-derived clinical candidate from X-Chem's platform to reach this regulatory stage.^19^

From 2020 onwards, DEL screening became much more prevalent in the scientific literature with more than twenty DEL derived compounds reported per year.^13^ During the COVID-19 pandemic, DEL screening was used as a highly effective tool in rapidly identifying possible treatments with CDD-1976 being reported as an effective inhibitor of the SARS-CoV-2 Mpro protein.^20^ Furthermore, DEL screening began to be utilised beyond classical small molecules, with X-Chem using DEL screening to discover a potent fragment for incorporation into a PROTAC against ERα.^21^ Most recently, Amgen has had success with PRMT5 inhibitor AMG 193 which was optimised from an initial DEL hit to give a strong clinical candidate.^22,23^ Clinical trials of AMG 193 began in 2022 with initial phase I results showing a good safety profile and promising anti-tumour activity.^24^ As of 2025, the number of reported novel molecules derived from DEL screening has risen to over forty publications per year. This highlights the increased prevalence of DELs in pharmaceutical screening which will naturally lead to more DEL-derived clinical candidates and hopefully lead to a greater clinical success rate.

## 2. On-DNA chemistry

Although DELs can screen vast numbers of compounds, their true value is limited by the chemical reactions that can be performed in the presence of DNA. These limitations are mostly associated with the immense challenge of developing DNA-compatible chemical reactions which can be applied to the ‘split and pool’ library synthesis. In order to avoid precipitation of the DNA conjugate, the solvent mixture of these reactions must consist of at least 20% water.^25^ Furthermore, dilute concentrations (∼1 mM) are typically needed and harsh conditions such as high temperature (>80 °C), strong acids (pH < 4) and strong oxidants or reductants generally need to be avoided.^25,26^ In this light, buffer systems (*e.g.* phosphate, borate, or carbonate buffers) are often included to control pH during the reaction. These constraints significantly limit the use of much of the synthetic chemistry toolbox and thus necessitates the development of creative solutions.^27^ Therefore, until the appropriate on-DNA chemistry is developed, there will be classes of compounds that are inaccessible for incorporation into DEL libraries.

Since the development of DNA-compatible reactions is crucial to the success and efficacy of DELs, significant efforts have been made by both academia and industry to develop and adapt common chemical transformations to be applicable to on-DNA synthesis. Due to the reaction constraints described previously, the development of on-DNA synthetic protocols is far different from typical synthetic chemistry. On-DNA reactions are usually carried out on a nanomolar scale in dilute conditions (∼1 mM), therefore, it is common for reagents and coupling partners to be used in vast excess (20–1000 equivalents) and catalysts are often used stoichiometrically. Although near-quantitative conversion is desirable, DEL synthesis is not governed by the same yield requirements as preparative organic synthesis. Because library members are not isolated individually, reactions with moderate conversion can still be useful provided that the desired product is formed cleanly, the DNA barcode remains amplifiable, and sufficient product survives selection and sequencing.^28^ When developing new on-DNA transformations a common headpiece is typically used: 5′-/5Phos/GAGTCA/iSp9/iUniAmM/iSp9/TGACTCCC-3′ (Scheme 3). This tag has a CC overhang which can be used to carry out subsequent ligation and amplification studies post reaction and ensure that these processes are not compromised by the developed reaction as this would limit its utility in library building.

**Scheme 3:**
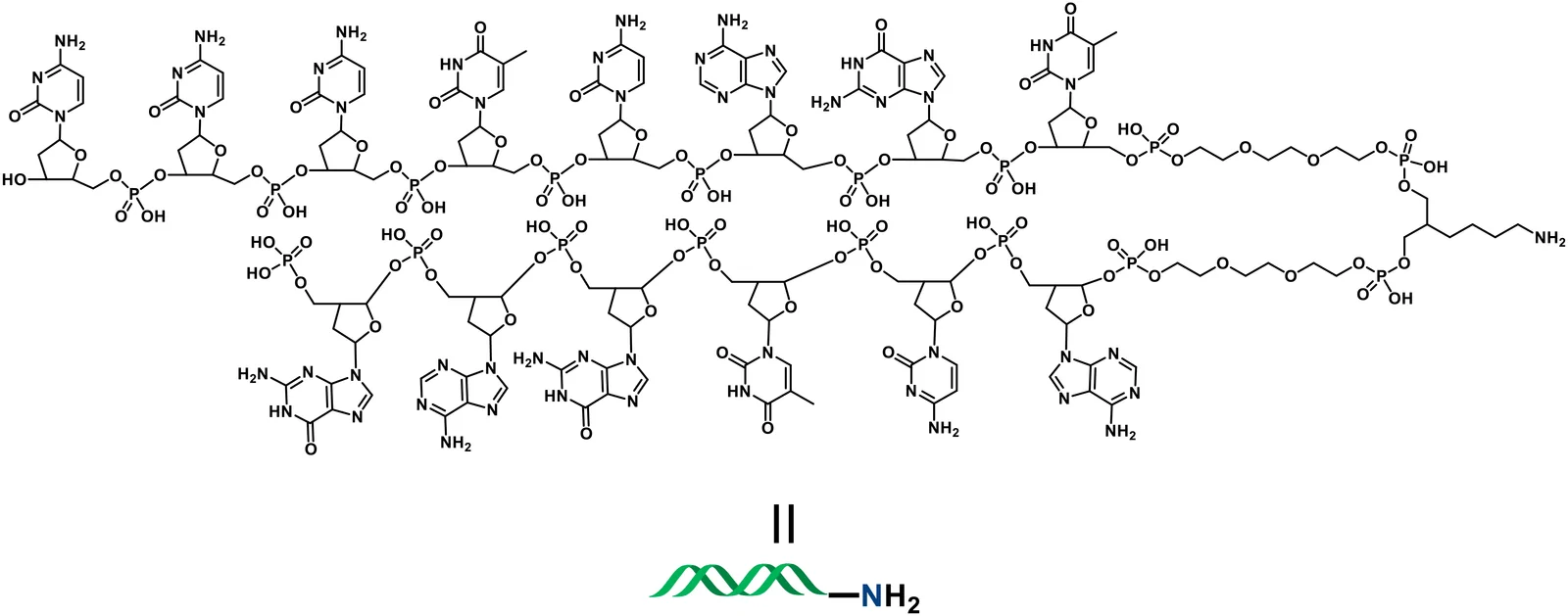
Structure of the AOP extended DNA headpiece (5′-/5Phos/GAGTCA/iSp9/iUniAmM/iSp9/TGACTCCC-3′) used in the development of novel on-DNA chemical transformations.

### 2.1 Classical on-DNA chemistry

On-DNA chemistry starts with an initiation reaction whereby a chemical scaffold is conjugated to a DNA tag. Following the conjugation of the initial scaffold, there is a range of diversification reactions which make up the split and pool cycles and contribute to library building and expansion. The on-DNA amide coupling reaction has been the most developed and commonly utilised initiation process and is most easily achieved by coupling the amine terminus of the commercially available DNA headpiece HP-1 with a carboxylic acid 1 to give functionalised headpiece HP-2. The extensive optimisation of this reaction has led to a range of effective strategies being proposed with varying effectiveness depending on the carboxylic acid substrate 1 (Scheme 4A).^29–31^ Reductive amination offers a complementary approach using an aldehyde 2 and the amine terminus to afford an amine-conjugated scaffold (HP-3 and HP-4) as an alternative to an amide (Scheme 4A).^32^ These two approaches allow for tailoring of the electronic properties of the scaffold which can be tuned to enhance downstream reactivity.

**Scheme 4:**
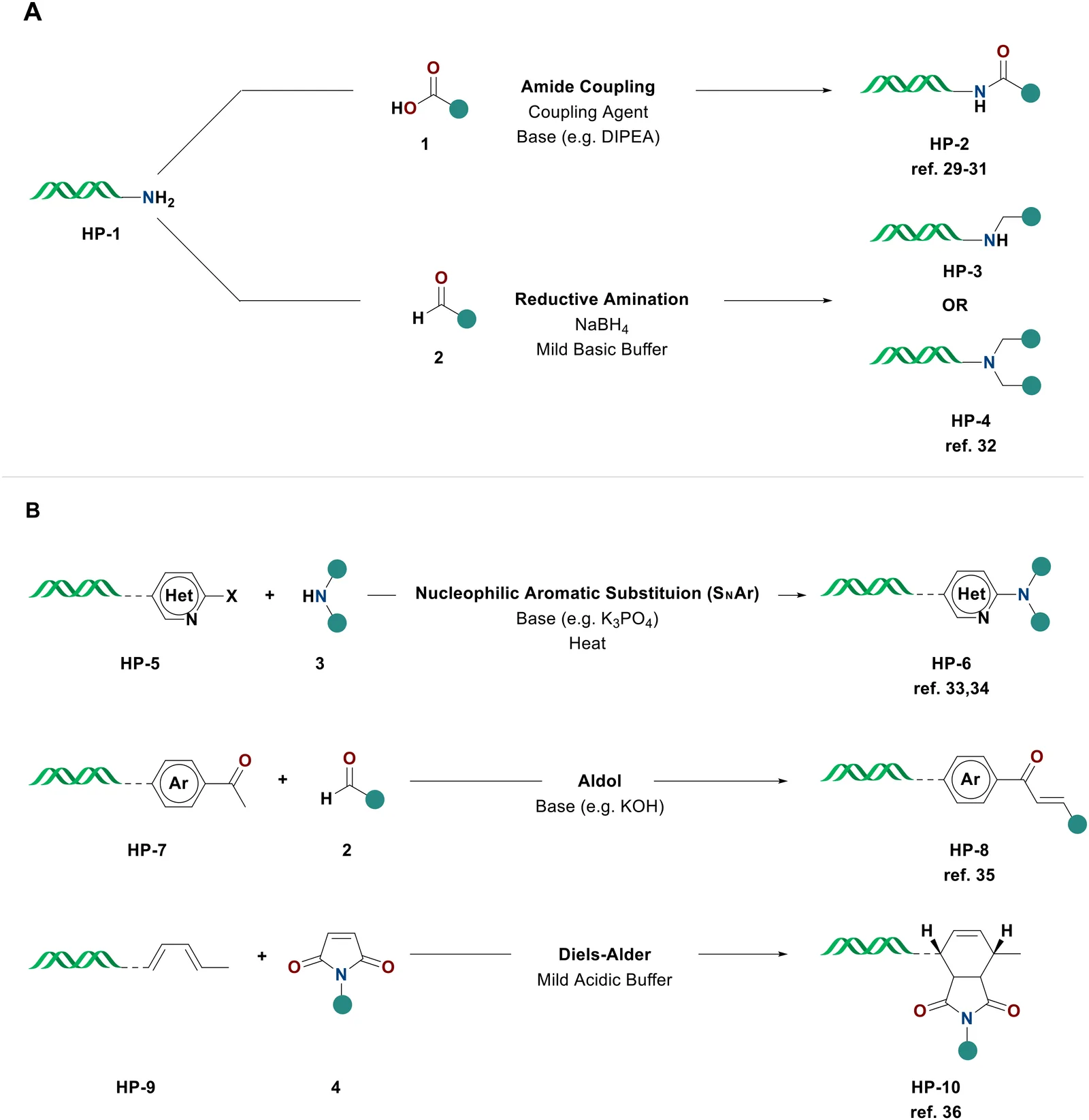
Overview of common chemical transformations on-DNA. (A) Initiation reactions such as amide coupling and reductive amination whereby an initial chemical scaffold is conjugated to a DNA-tethered amine. (B) Diversification reactions which act to decorate the initial scaffold such as nucleophilic aromatic substitution reactions (S_N_Ar), aldol reactions and Diels–Alder reactions.

Beyond amide chemistry, a suite of classical reactions has been translated for use in aqueous, micromolar-scale DNA contexts (Scheme 4B). For example, nucleophilic aromatic substitution (S_N_Ar) enables late-stage aryl halide functionalisation;^33,34^ aldol reactions allow for C–C bond formation,^35^ and Diels–Alder cycloadditions install bicyclic scaffolds.^36^ Each reactivity class operates below 80 °C and at pH 7–9, preserving DNA integrity while giving up to quantitative conversions. The value of these classical transformations lies in their broad commercial availability. Carboxylic acids, aldehydes, amines, activated heteroaryl halides, carbonyl compounds, dienes and dienophiles are all readily accessible, allowing these reactions to translate simple catalogue fragments into encoded library diversity. As a result, these methods have become core DEL reactions because they are reliable, modular and compatible with the split and pool logic of library construction.

### 2.2 Transition metal catalysed on-DNA chemistry

Metal-catalysed cross-couplings have been key for the expansion of DEL chemical space beyond the polar functional-group logic of amide or reductive amination chemistry. Adapting these transformations for micromolar, aqueous DNA settings has required careful selection of (pre-)catalyst, base, solvent and temperature, but several robust protocols are now routine (Scheme 5A). In recent years, reactivity of these transition metal processes has been enhanced by advances in micelle-promoted, on-DNA chemistry. This was highlighted by Waring, who used the TPGS-750-M surfactant to create a lipophilic core around organic reaction components and catalysts, separating them from the DNA tag and thus preventing unwanted interactions.^37–40^

**Scheme 5:**
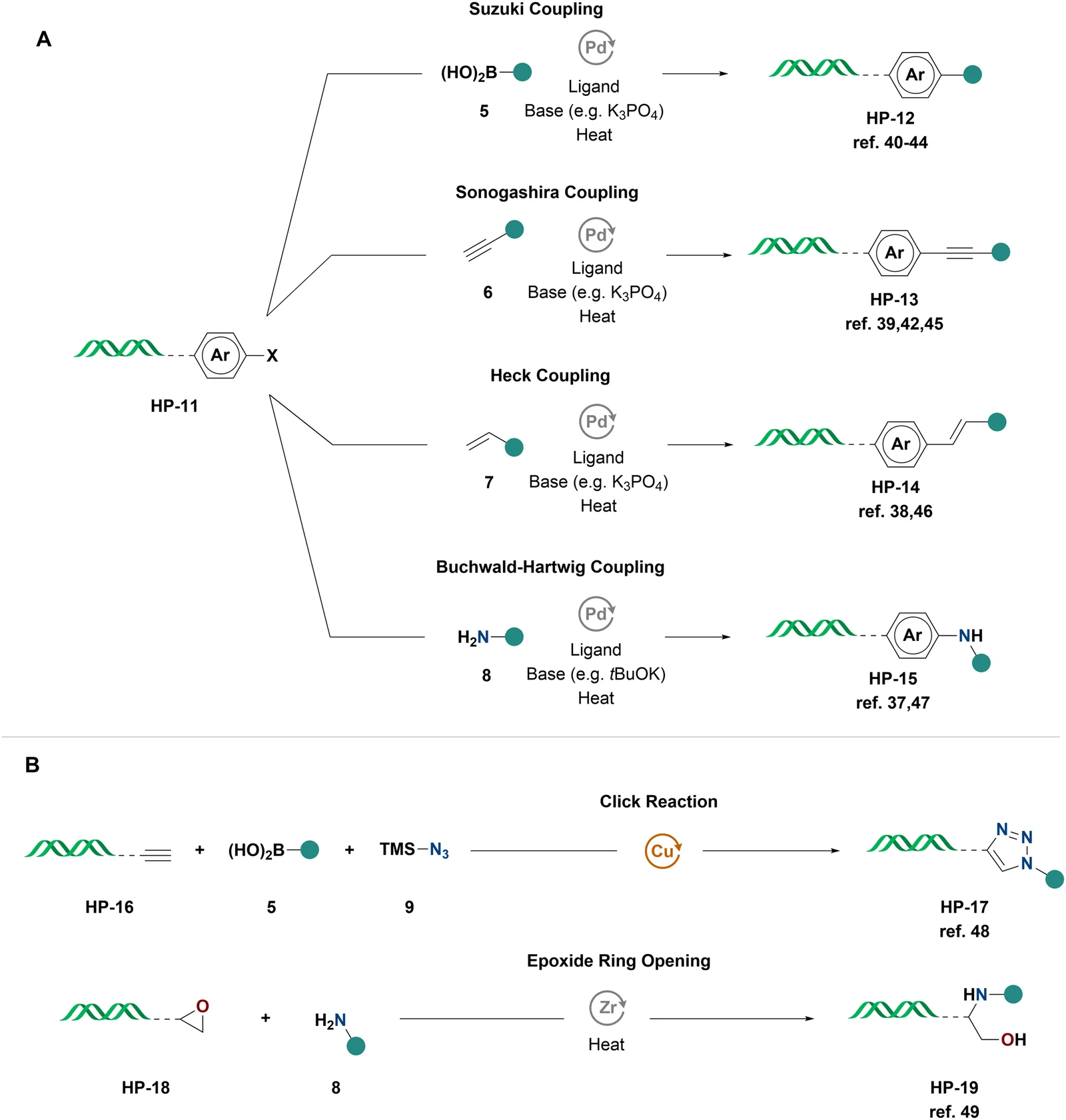
Overview of common transition-metal-catalysed reactions on-DNA. (A) On-DNA palladium-catalysed cross couplings including Suzuki couplings, Sonogashira couplings, Heck reactions and Buchwald–Hartwig couplings. (B) On-DNA reactions catalysed by other transition metals such as the copper-catalysed Click reaction and the zirconium-catalysed epoxide ring opening.

The Suzuki–Miyaura coupling is the most widely reported on-DNA metal-catalysed reaction, coupling DNA conjugates aryl halide HP-11 with boronic acid 5 to give HP-12 under palladium catalysis.^40–44^ Similarly, DNA-compatible Sonogashira,^39,42,45^ and Heck couplings,^38,46^ with alkynes 6 and alkenes 7 affording HP-13 and HP-14 respectively have also been extensively studied. The installation of aryl amines in HP-15 in an on-DNA Buchwald–Hartwig coupling between HP-11 and 8 has been recently reported.^37,47^ Beyond palladium-catalysed coupling, the on-DNA Click reaction is also well established on HP-16 utilising a copper(ii) catalyst in the presence of trimethylsilyl azide 9 and a variable boronic acid 5 to induce functionality and generate triazole HP-17 (Scheme 5B).^48^ Furthermore zirconium catalysis has also been used in the ring opening of DNA-conjugated epoxides HP-18 with amines 8 to construct amino alcohols in HP-19.^49^

The development of these on-DNA coupling reactions was revolutionary in allowing for diverse library expansion across a range of abundant chemical building blocks. However, most of these transition-metal-mediated reactions necessitated heating to 50–80 °C to achieve useful conversions, pushing to the edge of the tolerance window for on-DNA synthesis. Furthermore, there is a risk of trace metal coordination to nucleobases or transition-metal-promoted DNA degradation which can compromise PCR amplification or downstream biochemical assays.^50,51^

## 3. On-DNA photochemistry

While transition-metal cross-couplings have transformed on-DNA synthesis, their impact has largely been in the construction of sp^2^-rich products through reliable bond-forming diversifications.^52–54^ The next challenge for DEL chemistry is to move beyond simple substitution and generate molecular complexity more efficiently. This is particularly important because most DELs are assembled in only two to three encoded synthetic cycles, leaving limited opportunities to build shape, stereochemical information and heteroatom-density into each library member. Reactions that can form rings, increase sp^3^-character or generate privileged three-dimensional structures in a single DNA-compatible step are therefore highly valuable. However, achieving this under the constraints of on-DNA synthesis remains a major challenge.^55^

Photochemistry provides a powerful way to address this challenge. Excited-state activation opens reaction pathways that are difficult, or impossible, to access using conventional ground-state chemistry, allowing simple DNA-conjugated precursors to be converted into structurally complex products under comparatively mild conditions. This is particularly attractive for DEL syntheses, where increasing molecular complexity per encoded step is often more valuable than simply increasing the number of building blocks. Photochemical methods can operate through several distinct activation modes, including single-electron transfer (SET), energy transfer (EnT), hydrogen-atom transfer (HAT), electron donor–acceptor (EDA) complex formation, metallaphotoredox catalysis and direct photolysis. In many cases, these manifolds are enabled by photocatalysts (PCs) whose excited-state redox potentials or triplet energies can be tuned to match the target transformation (Scheme 6, see Fig. 1 for a full list of PCs covered in this review).^56–60^ At the same time, photochemistry introduces additional parameters that are key on-DNA, including wavelength, light intensity, oxygen sensitivity, photocatalyst compatibility and potential DNA degradation. These opportunities and constraints define the emerging field of on-DNA photochemistry. For these methods to translate successfully to split-and-pool DEL synthesis, they must also tolerate large and chemically diverse building-block sets under a common set of reaction conditions. Most reported on-DNA photochemical transformations have so far been evaluated primarily through scope studies using short DNA headpieces, meaning that their robustness at true library scale remains underexplored. Nevertheless, there are encouraging examples of translation beyond this stage. Yen-Pon and co-workers demonstrated compatibility of their photochemical methodology with a full-length DNA tag, while Suo and co-workers used a light-promoted reaction to construct and screen a >100 000-member indazolone DEL against p300.^61,62^ These studies demonstrate that photochemical reactions can be sufficiently robust for DEL construction, although validation across the large and diverse building-block collections required for routine library production remains limited.

**Scheme 6:**
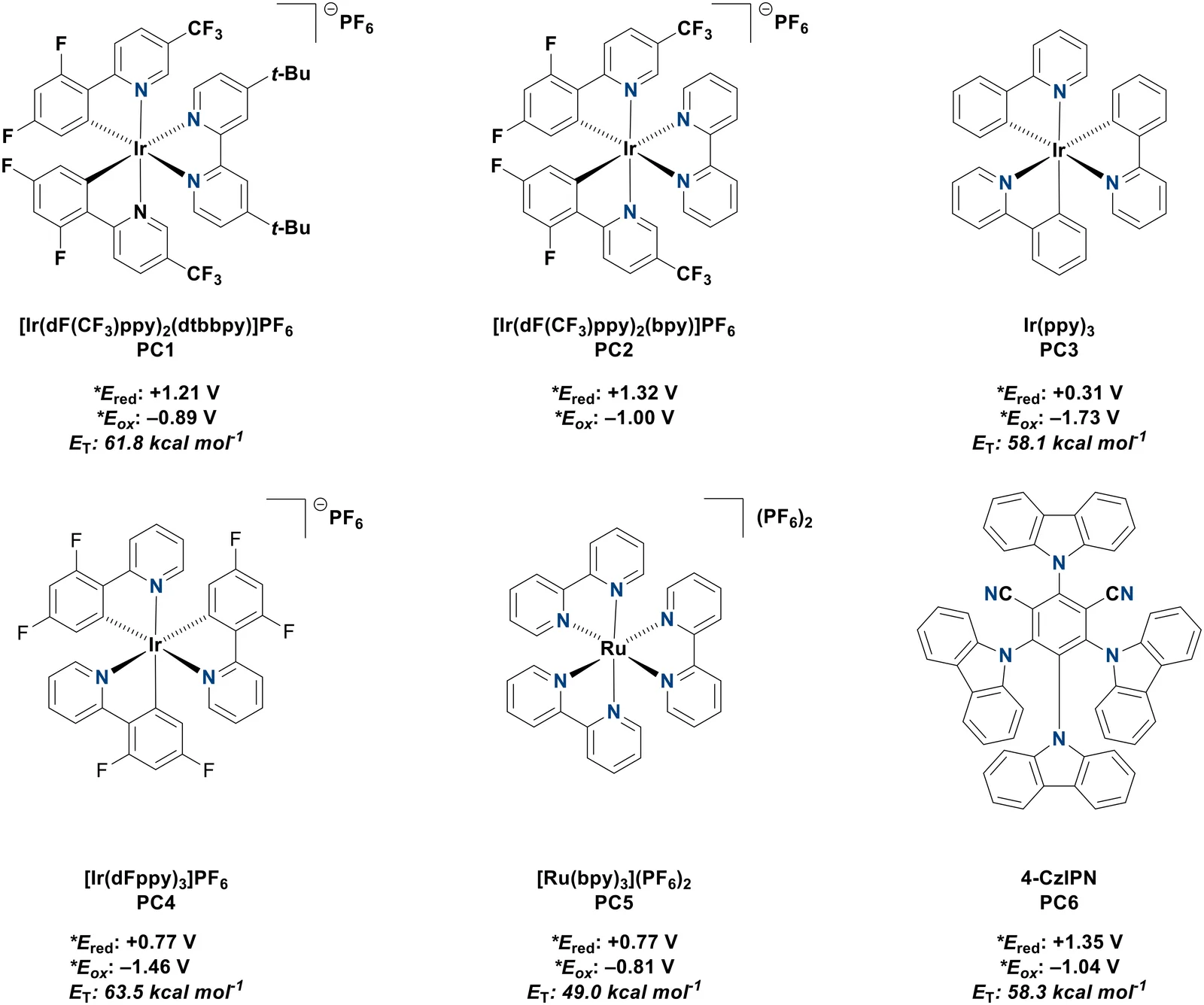
Selection of common iridium-based, ruthenium-based and organic photocatalysts used in photochemical reactions. Redox potentials are referenced to the saturated calomel electrode (SCE). See Fig. 1 for a comprehensive list of the photocatalysts covered in this review.

**Fig. 1:**
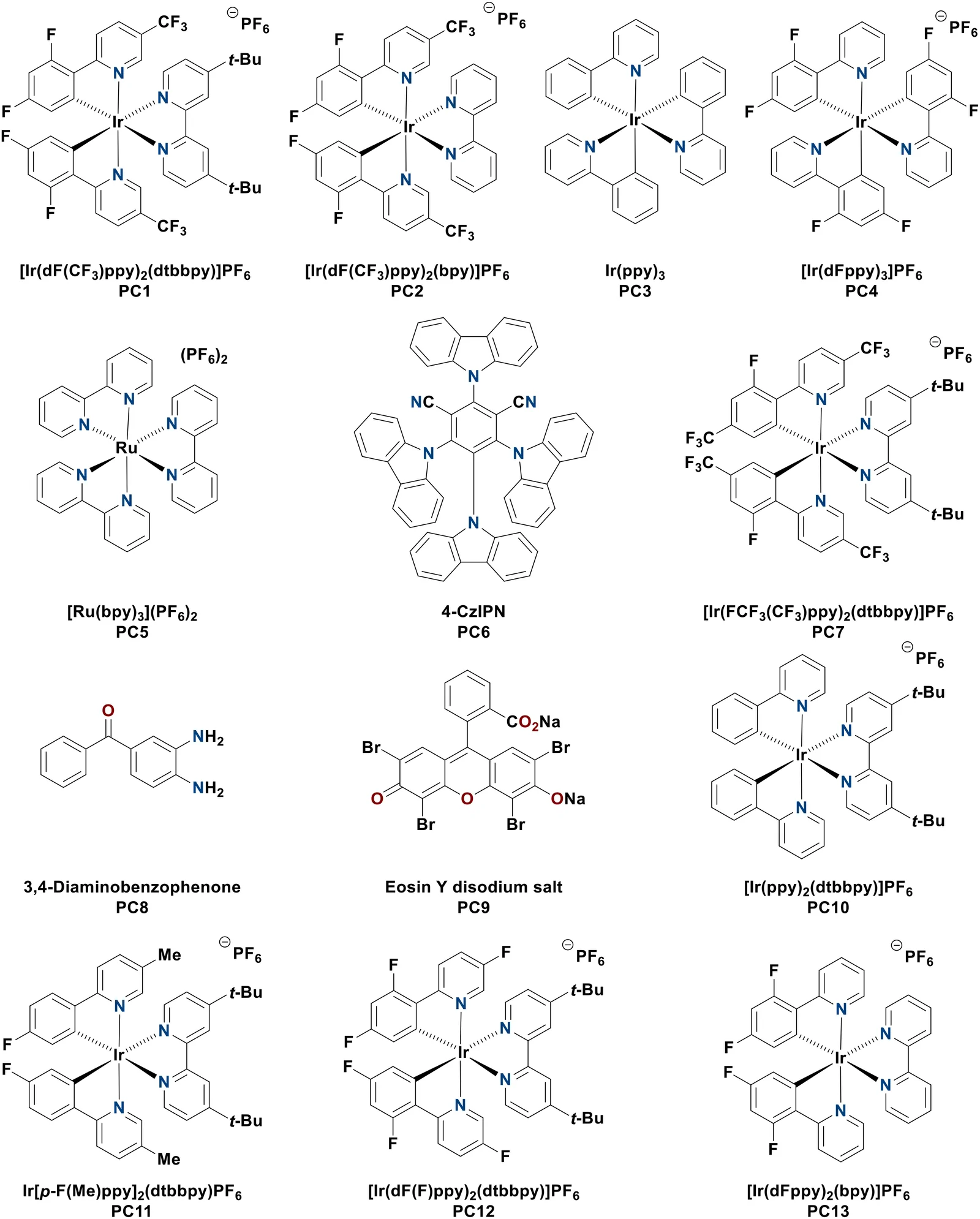
Chemical structures for all the inorganic and organic photocatalysts used for photocatalytic reactions in this review.

Recent advances in photochemical cycloaddition chemistry highlight how excited-state reactivity can rapidly convert simple feedstocks into complex, medicinally relevant structures. Glorius and Brown reported a photochemical [4 + 2] cycloaddition reaction towards bridged bicyclic heterocycles of type 12 from simple quinoline 10 and alkene 11 (Scheme 7A).^63,64^ Beyond this, Tortosa demonstrated how the direct irradiation of diene 13 in the presence of a rhodium Lewis acid catalyst (Λ-RhS) could afford enantioenriched bicyclo[2.1.1]hexane 14, providing a simple, one-step route towards benzene bioisosteres (Scheme 7B).^65^ Both of these reactions provide access to complex, bridged bicyclic fragments with enhanced physicochemical properties.^66^ Finally, Dinér presented a facile route towards nitrogen-containing spirocyle of type 17 from redox-active ester 15 and alkene 16.^67^ Again this is a simple, one-step transformation with minimal additives (Scheme 7C). These spirocyclic compounds demonstrate high molecular complexity and are increasingly sought after due to their rigid, sp^3^-rich nature.^68^

**Scheme 7:**
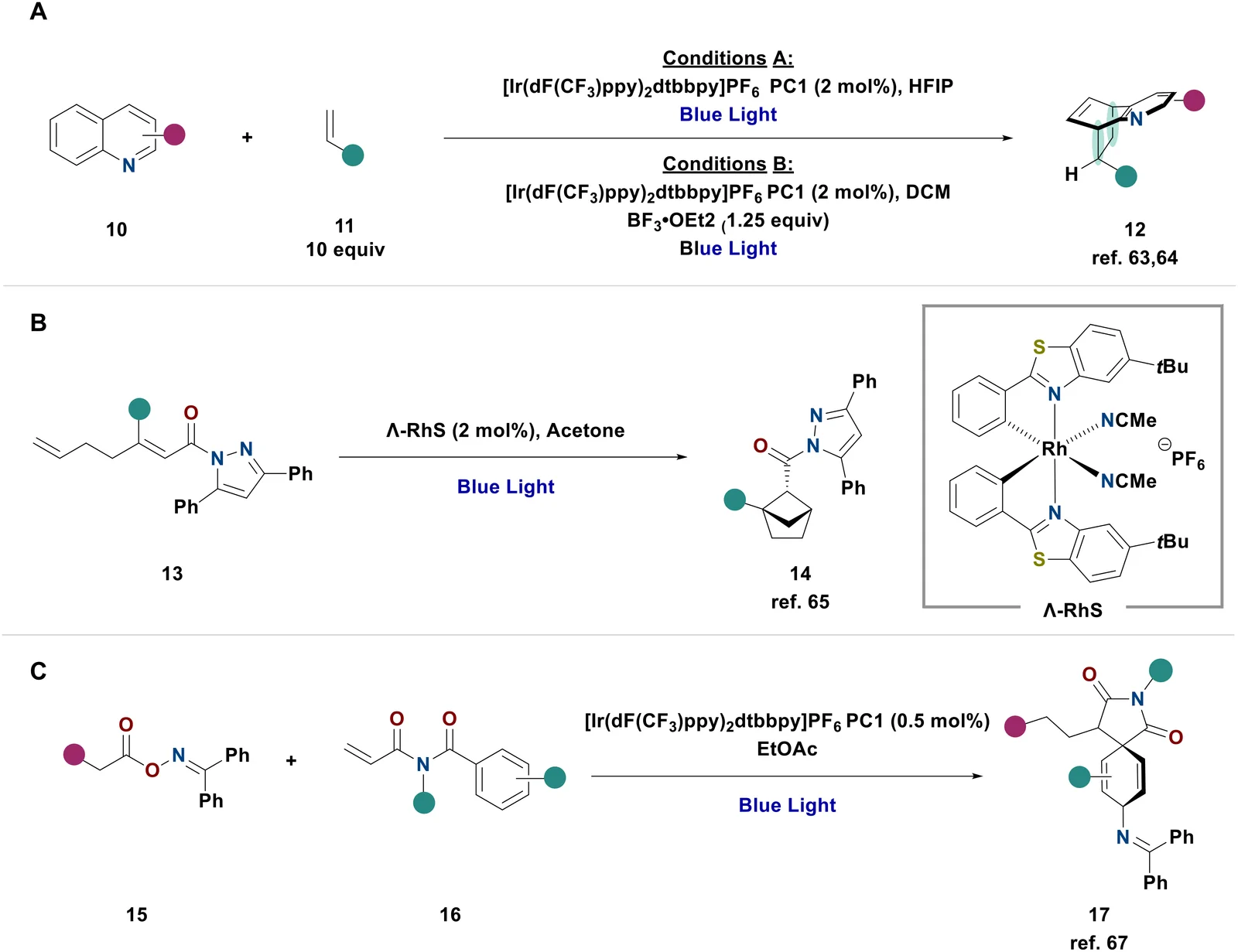
Overview of novel off-DNA photochemical transformations that build molecular complexity under mild conditions. (A) A [4 + 2] photocycloaddition reaction between quinolines 10 and alkenes 11 to afford bridged bicyclic products 12. (B) Lewis acid catalysed, intramolecular, photochemical [2 + 2] reaction of 13 to afford bicyclo[2.1.1]hexane 14. (C) The energy-transfer (EnT) catalysed synthesis of spirocycles 17 from redox-active ester 15 and alkene 16.

In this light, the adaptation of photochemistry towards on-DNA synthesis provides the potential for these complex, medicinally relevant fragments to be incorporated into DEL screening.^25,69,70^ Furthermore, while transition metal catalysis pushes the platform toward its thermal and chemical limits, visible-light photocatalysis offers a complementary, much milder manifold: reactions typically run at room temperature in aqueous buffer, with catalyst loadings ≤200 mol% and negligible DNA degradation or metal carry-over.^25,69^ Despite this, on-DNA photochemistry has some additional considerations. Light intensity and lamp distance are important factors in controlling photon flux which can greatly impact reactivity and/or DNA degradation. Furthermore, photochemical reactions carried out in the presence of oxygen can promote DNA damage through both single-electron transfer (SET) and energy-transfer (EnT) pathways. In SET processes, an excited photocatalyst can participate in electron transfer with either the nucleobase or molecular oxygen, generating reactive radical ions and ROS such as superoxide (O_2_˙^−^). Alternatively, EnT from an excited photocatalyst to ground-state triplet oxygen can generate singlet oxygen (^1^O_2_).^71–73^ Of the canonical nucleosides, deoxyguanosine (dG) is particularly susceptible to these oxidative processes.^72^ Photooxidation of dG can give oxidised products including spiroiminodihydantoin (dSp) and oxazolone (dZ), although the product distribution depends on the mode of photocatalytic activation. For example, riboflavin-mediated oxidation gives dSp and dZ in near-equal amounts, while anthraquinone predominantly gives dSp with some dZ. In contrast, singlet oxygen generated through EnT using Rose Bengal gives dSp exclusively.^72^ This damage is particularly problematic if dG forms part of the ssDNA overhang, where modification could prevent ligation of the subsequent DNA strand and hinder further encoding. Furthermore, nucleobase modification can compromise subsequent ligation and amplification, reducing the amount of amplifiable DNA available for barcode decoding.^74^

Beyond oxidative nucleoside damage, direct excitation of DNA can generate additional photolesions. DNA absorbs strongly in the UV region, with an absorption maximum around 260 nm, making direct photochemical damage particularly important under UVC and UVB irradiation.^75,76^ Excitation of adjacent pyrimidine bases can promote [2 + 2] cycloaddition to give covalent intrastrand photolesions known as cyclobutane pyrimidine dimers (CPDs, Scheme 8B).^75,76^ CPD formation is particularly efficient at short UV wavelengths and remains significant throughout the UVB region, although CPDs can also be generated under UVA irradiation with much lower efficiency.^75,76^ If these lesions persist, they can inhibit PCR amplification and DNA sequencing and render the barcode inaccessible.^74–76^

**Scheme 8:**
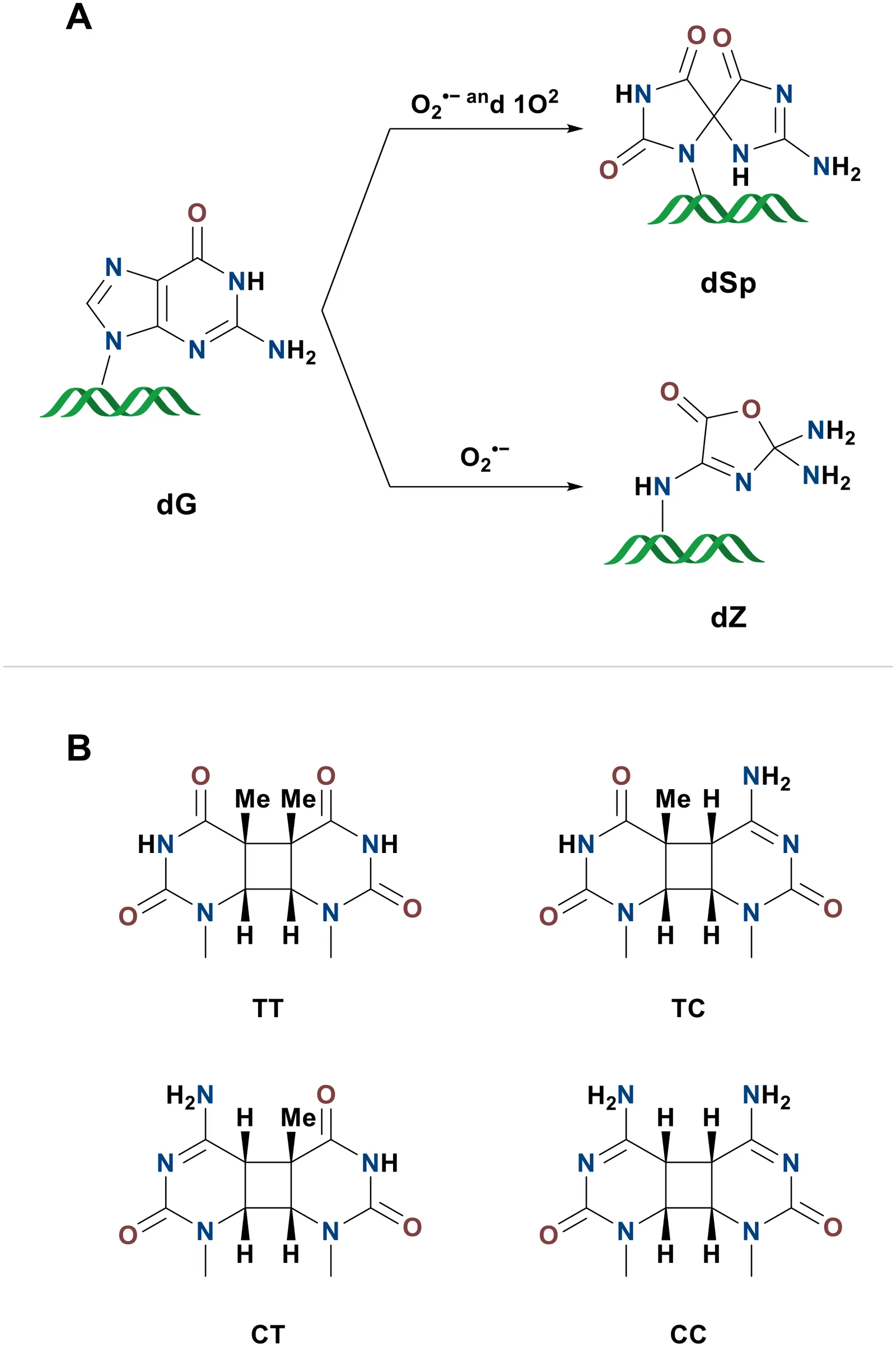
Modes of DNA damage. (A) Oxidation of guanine (dG) nucleoside with superoxide (O_2_˙^−^) and singlet oxygen (^1^O_2_) to form dSp or dZ. (B) Cyclobutane pyrimidine dimers (CPD) formed by the absorption of UV light (<315 nm) by DNA pyrimidine bases.

To circumvent these damaging processes, careful optimisation is required. For example, the wavelength of light must be carefully selected so that it is sufficiently energising to activate the desired reaction but not too much so as to induce DNA crosslinks or other forms of DNA damage. Similarly, the selection of a PC is a vital decision as the redox potentials and triplet energies must be finely tuned so that the PC sensitises the desired motif while minimising the formation of ROS. Furthermore, these reactions can require the presence of an inert atmosphere (either a glovebox or carrying out the reaction under a flow of argon or nitrogen) or the addition of a mild radical scavenger such as glycerol.^77^ In this case, however, it is important to consider the practical complexity of such techniques, as reactions that are simple to set up are more desirable to users when constructing DELs.

In this review, an extensive range of on-DNA photochemical, photocatalysed and light-driven processes will be explored. Different modes of radical activation and coupling will be detailed including photoredox single-electron transfer (SET), hydrogen-atom abstraction (HAT), metallaphotoredox chemistry whereby SET is coupled to transition metal catalysis, photoactivation following the formation of an EDA complex and photolysis-enabled reactions.

### 3.1 On-DNA photoredox single electron transfer (SET)

Upon irradiation with blue light, the ground-state Ir(iii) complex absorbs a photon to generate a short-lived singlet excited state, which rapidly undergoes intersystem crossing to a longer-lived triplet metal-to-ligand charge-transfer excited state, *Ir(iii). This excited state possesses substantially altered redox properties compared with the ground-state catalyst, enabling it to act as either a strong oxidant or reductant, depending on the reaction partner and the relevant redox potentials (Scheme 9). During reductive quenching, the excited photocatalyst, PC*, accepts an electron from an electron donor (ED), generating the reduced photocatalyst, PC˙^−^, and the corresponding donor radical cation, ED˙^+^. When the electron donor is itself a radical precursor, the resulting donor radical cation, ED˙^+^, may undergo fragmentation, deprotonation or bond cleavage to generate a reactive radical intermediate. Alternatively, the electron donor may act as a sacrificial reductant, with PC˙^−^ subsequently reducing a separate radical precursor. This resulting radical intermediate can then attack a second coupling partner to generate a second radical intermediate. In one common redox-neutral pathway, this radical intermediate subsequently accepts an electron from PC˙^−^ in a second SET event. This regenerates the ground state PC and converts the radical intermediate into a polar anion which upon protonation, completes the formation of the coupled reaction product. Conversely, during oxidative quenching, the excited PC* transfers an electron to an electron acceptor (EA), generating the oxidised photocatalyst, PC˙^+^, and the corresponding acceptor radical anion, EA˙^−^. The electron acceptor may itself be a radical precursor or may function as a separate oxidant. This SET event can generate a substrate radical anion, which may undergo fragmentation, extrusion or bond cleavage to release a reactive radical intermediate. As before, this reactive radical can add to a second coupling partner to generate another radical intermediate which can in turn donate an electron to the PC radical cation and thus close the catalytic cycle. In some cases, additional additives may be required to promote fragmentation of the radical precursor, facilitate subsequent proton-transfer or polar steps, or complete regeneration of the ground-state photocatalyst and this will be described where applicable.

**Scheme 9:**
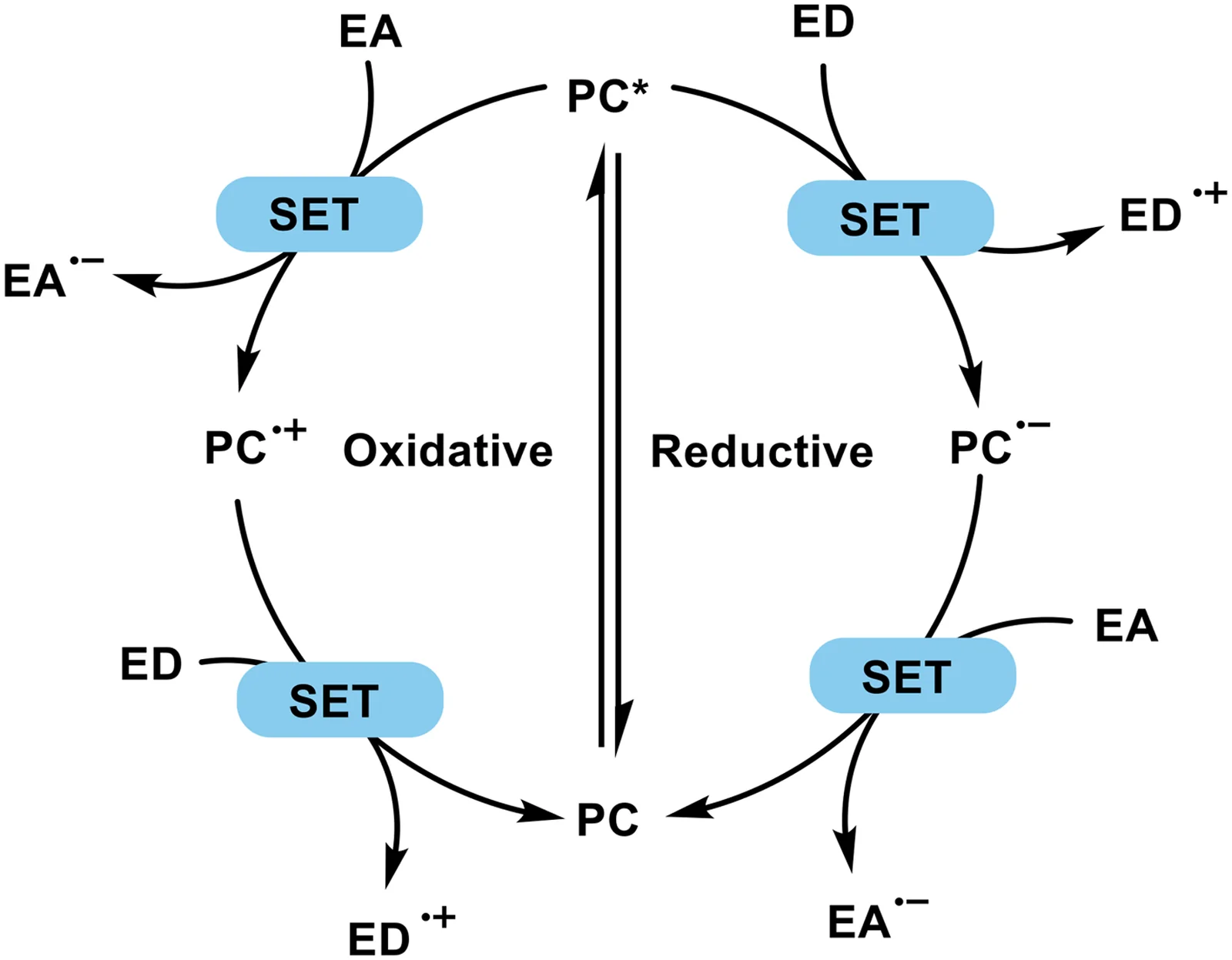
Oxidative and reductive quenching catalytic cycle of a photocatalyst (PC). In an oxidative quenching cycle the excited PC* donates an electron to an electron acceptor (EA) forming a photocatalyst radical cation species that then accepts an electron from an electron donor (ED) to return to the ground state. In a reductive quenching cycle, the excited PC* accepts an electron from an electron donor (ED) forming a photocatalyst radical anion species that then donates an electron to an electron acceptor (EA) to return to the ground state.

The utility of photoredox chemistry in the context of on-DNA chemistry was pioneered by Flanagan at Pfizer in 2018 with their photoredox-mediated Giese coupling between DNA-conjugated activated alkene HP-20 and simple amino acids 18 to afford product HP-21 in the presence of an iridium PC.^78^ This reaction proceeded in a reductive quenching pathway as described previously whereby decarboxylation of the amino acid releases an α-amino radical intermediate. DNA-tagged acrylamide HP-20 was used as a model substrate for optimisation and scope studies and was found to be a suitable coupling partner for a wide range of α-amino radicals (Scheme 10). Primary (18a), secondary (18b), and tertiary (18c) *N*-Boc-protected amino acids were all seen to be well tolerated with increasing substitution allowing for higher yields due to the formation of more stable radical intermediates.^78^ Aromatic side chains (18d and 18e) along with carboxylic acid side chains (18f) also gave good to excellent yields. In the case of the carboxylic side chain in 18f, the reaction was seen to be regioselective towards reactivity at the α-amino site.^78^ This was due to the enhanced stability of a secondary α-amino radical intermediate over a primary alkyl radical. Similarly excellent yields were observed with privileged medicinal chemistry fragments such as heterocyclic α-amino acids 18g–h while the heterocyclic α-oxo acid counterpart 18i gave a good yield.^78^ However, when the amino acid was replaced with a secondary alkyl carboxylic acid, a significant decrease in the yield was observed (18j).^78^ This highlights the necessity of an α-heteroatom to stabilise the radical intermediate in this case. A range of activated alkene radical acceptors were also explored and seen to be widely tolerated when coupled with a phenylalanine radical precursor (19). Other Michael acceptors were seen to be amenable to 1,4-addition under these conditions. For example, HP-22 and the cyclic α,β-unsaturated ketone HP-23 reacted in moderate to good yields while styrene HP-24 and vinylpyridine HP-25 were similarly successful and thus allowed access to a new class of products.^78^

**Scheme 10:**
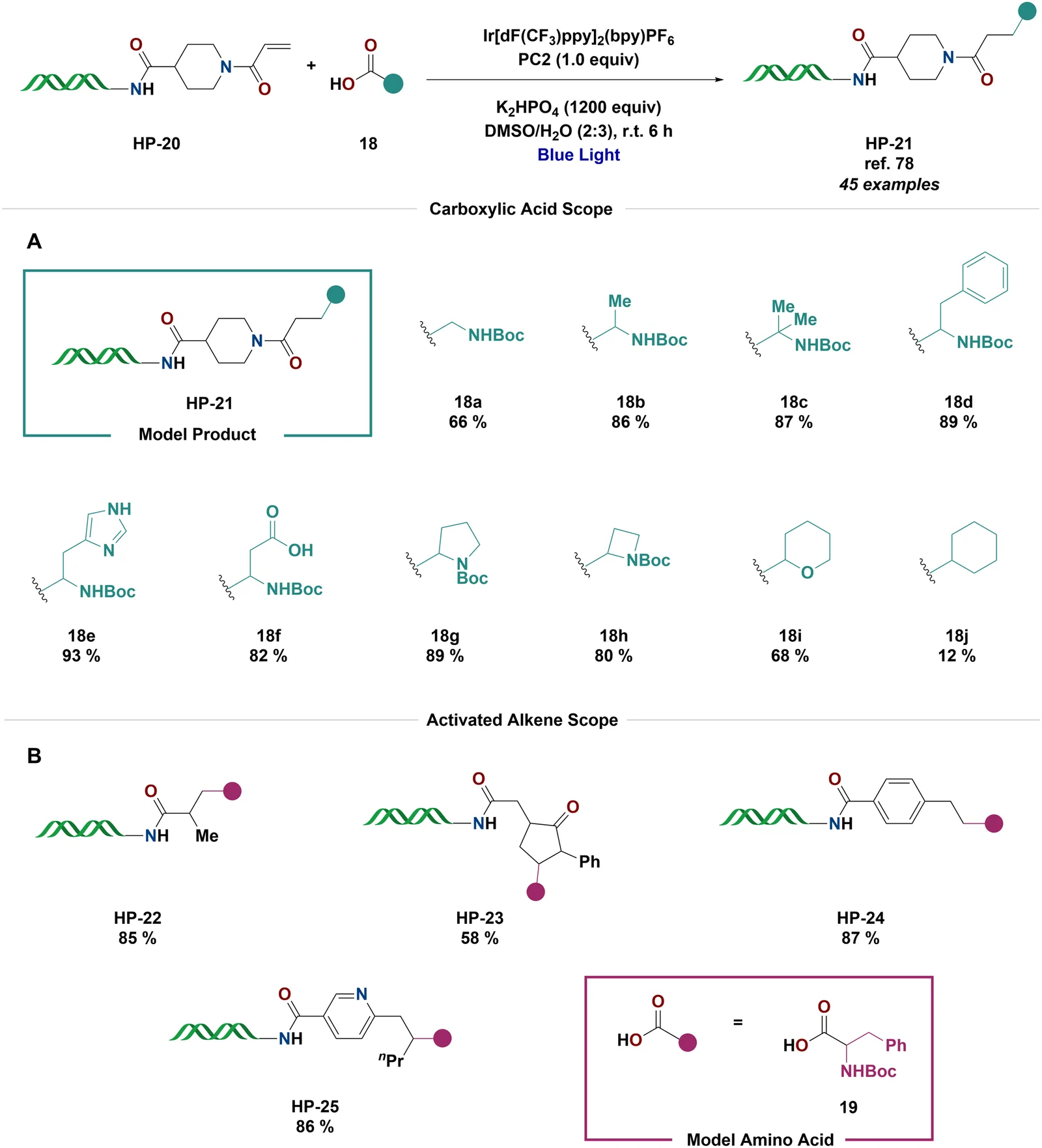
On-DNA Giese reaction between DNA conjugated alkene HP-20 and amino acid 18 towards the synthesis of HP-21. (A) Scope of amino acids compatible with the reaction with HP-20. (B) Scope of DNA-conjugated alkenes compatible with the reaction with amino acid 19.

Following on from this seminal work, several other on-DNA Giese reactions have been proposed using alternative radical precursors to widen the scope of coupling partners available (Scheme 11). Chheda recently disclosed an on-DNA Giese reaction facilitated by sodium xanthate radical precursors 20, which are particularly beneficial as they can be readily prepared from alcohol feedstocks.^79^ These conditions allowed for the facile coupling of alkyl radicals to both DNA-conjugated Michael acceptors and vinyl arenes (HP-26 and HP-27), thus significantly expanding the scope of this reaction as coupling to these substrates was previously seen to be poor with carboxylic acid radical precursors (*vide supra*, Scheme 10, compound HP-21j). In this process, a source of phosphine was required to facilitate the breakdown of the xanthate radical species in two sequential β-scission events to release the reactive intermediate which could react to form HP-28 and HP-29.^80^ Similarly, Liu (HitGen) demonstrated that alkyl radicals were accessible from imine precursors 21.^81^ Comparatively, these precursors show great promise as they can be easily synthesised from simple amines. Again, both Michael acceptors and styrenes (HP-26 and HP-27) were tolerated here giving HP-30 and HP-31 as the products. Finally, Niu developed a Giese process utilising sulfinate radical precursors 22 in order to couple glycosyl radicals to Michael acceptors or styrenes (HP-26 and HP-27) affording HP-32 and HP-33, thus further widening the scope of the reaction to a whole new class of substrates.^82^ In this light, the photocatalytic Giese reaction is considered the most robust and widely studied on-DNA photocatalytic reaction and has paved the way for the utility of photocatalytic reaction in DEL synthesis.

**Scheme 11:**
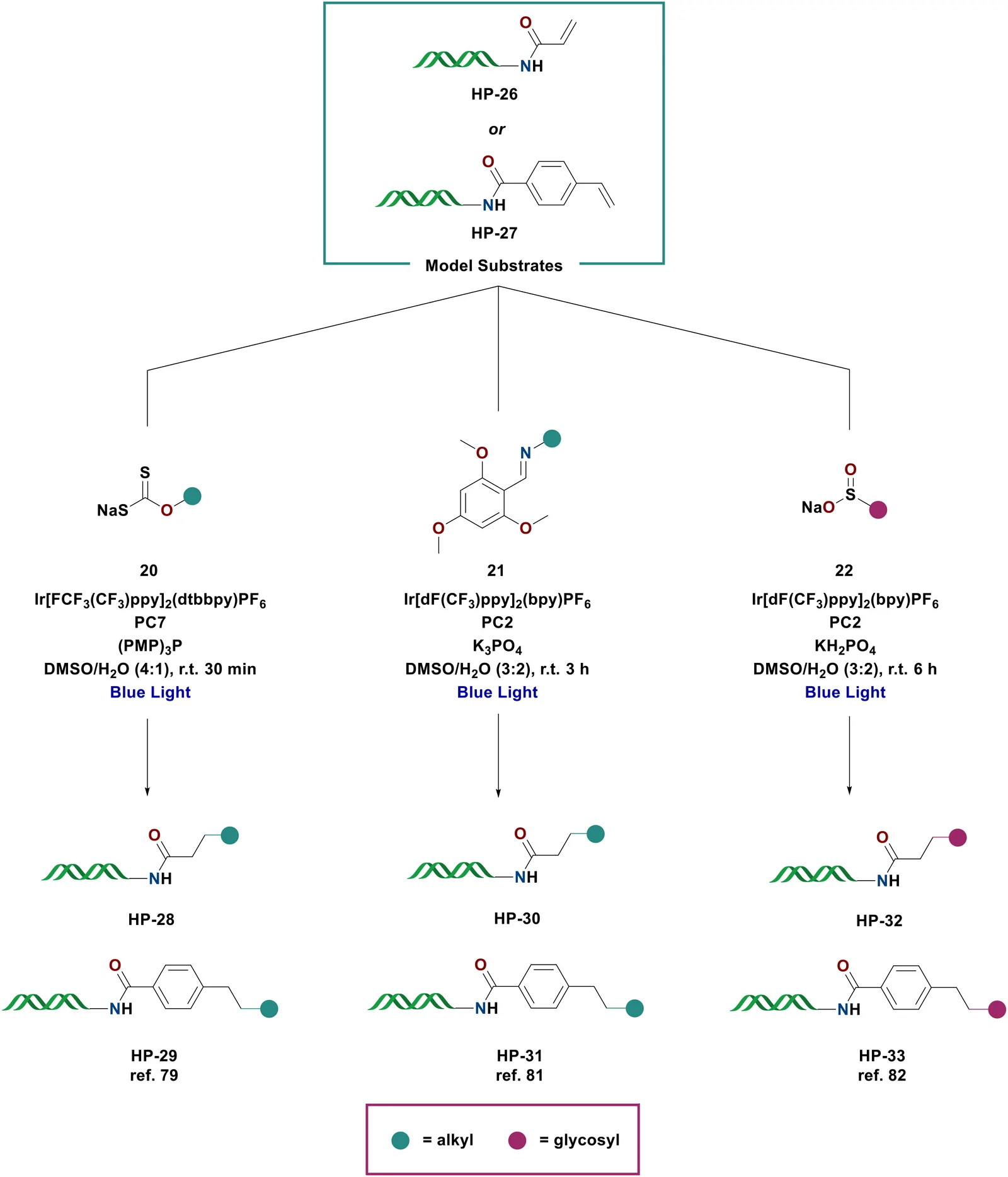
On-DNA Giese reactions on DNA conjugated Michael acceptors HP-26 and DNA-conjugated styrenes HP-27 using alternative radical precursors. Alkyl radicals were added using sodium xanthate 20 and imine precursors 21. Glycosyl radicals were added using sulfinate precursors 22.

Inspired by these advancements to the on-DNA Giese-type reaction, Molander developed a versatile photoredox catalysed, on-DNA synthesis towards *gem*-difluoroalkenes from DNA-conjugated, trifluoromethylated alkenes HP-34 (Scheme 12). This process was proposed to follow a radical polar crossover mechanism. This is similar to the Giese reaction in that it follows a reductive quenching pathway upon PC excitation. After initial radical coupling, the intermediate radical accepts an electron from the reduced photocatalyst to generate an anionic species and reform the ground state PC. However, rather than being protonated to give a saturated product as seen previously, the trifluoromethyl group can undergo a loss of fluoride in an E1cb-type process to generate a *gem*-difluoroalkene group.^83^ This chemistry was seen to be tolerable to a wide range of radical precursors (23) and photocatalysts and thus allows the radical precursor and photocatalyst to be matched to the target library design. Both carboxylic acid and α-silylamine precursors were extensively investigated and allowed for the coupling of amino acid derived moieties in moderate to good yield (24a and 25a–c).^28,84^ Both of these processes were facilitated by iridium-based catalysts. The scope of carboxylic acid precursors was expanded to include α-oxy species (24b) and simple alkyl carboxylic acids (24c).^28^ DHP and silicate radical precursors were also observed to be effective in the presence of 4CzIPN rather than an iridium photocatalyst, however, the scope of these precursors was seen to be limited to aliphatic groups (26a–c and 27a–c).^28^ The development of this procedure towards *gem*-difluoroalkenes is of great benefit to the pharmaceutical industry as these groups are becoming increasingly popular as carbonyl bioisosteres.^28^

**Scheme 12:**
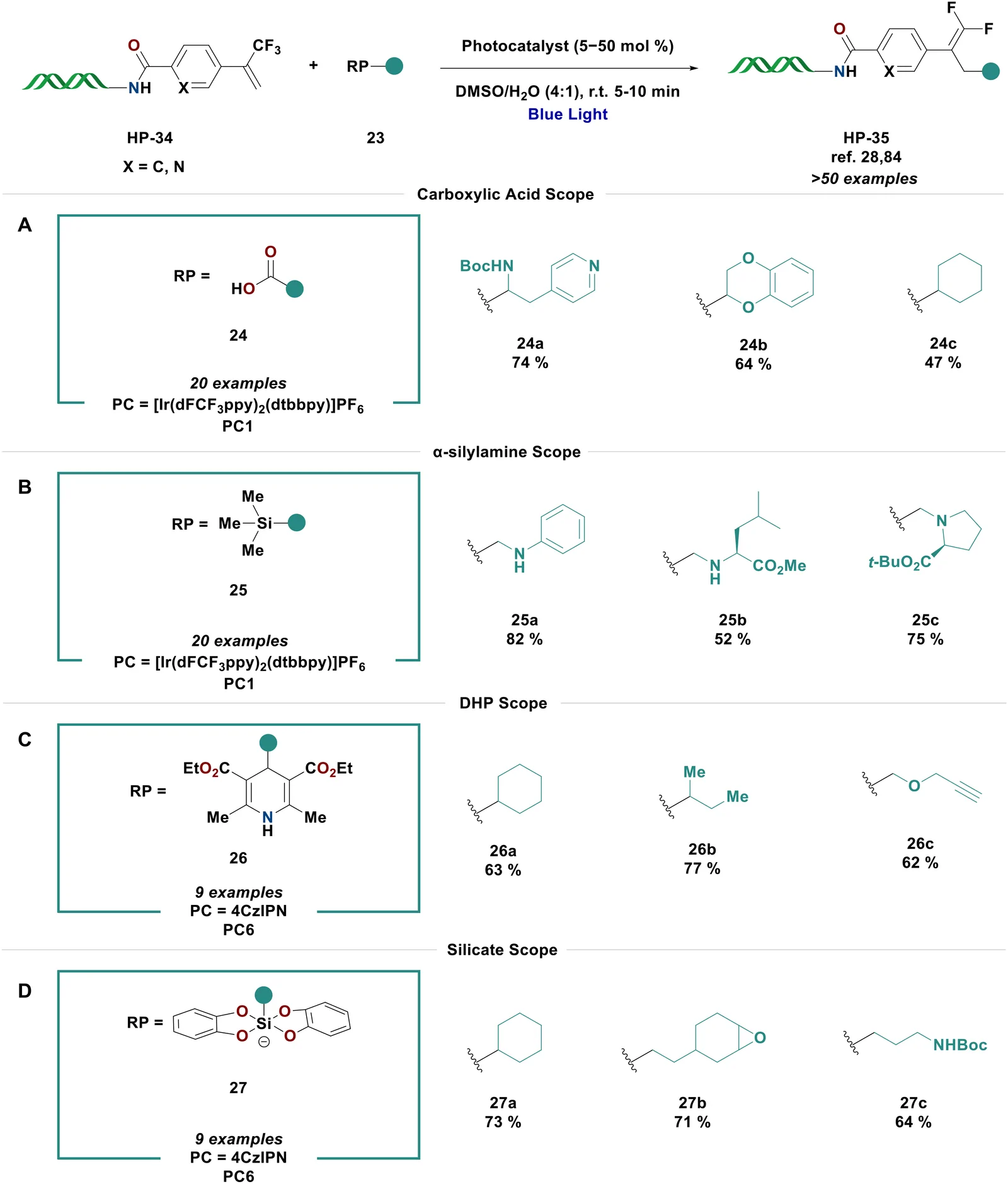
On-DNA reaction between DNA-conjugated alkene HP-34 and various radical precursors 23 towards the synthesis of *gem*-difluorinated alkene HP-35. (A) Scope of carboxylic acids 24 compatible with the reaction. (B) Scope of α-silylamines 25 compatible with the reaction. (C) Scope of DHP derivatives 26 compatible with the reaction. (D) Scope of silicates 27 compatible with the reaction.

Taking the well-known on-DNA Giese reaction and their novel on-DNA synthesis of *gem*-difluoroalkenes, Molander subsequently adapted these processes to be amenable to the incorporation of bicyclo[1.1.1]pentane (BCP) moieties in a halogen atom transfer process (XAT, Scheme 13).^61^ In the case of the on-DNA Giese reaction, BCP iodide 28 or alkyl iodide 29 were coupled with DNA-conjugated styrene HP-36 to afford products of type HP-37 or HP-38.

**Scheme 13:**
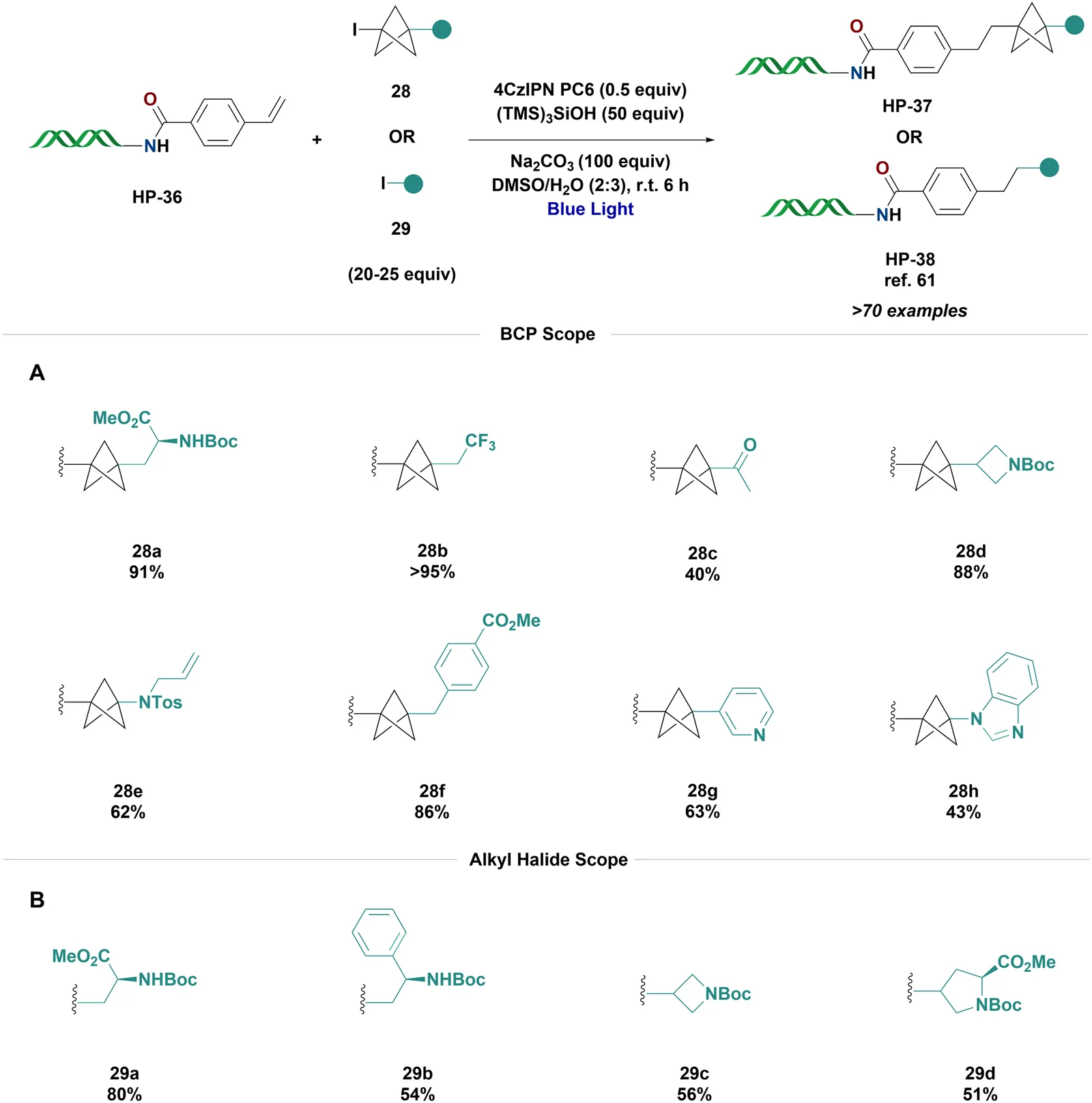
On-DNA Giese reaction between DNA-conjugated alkene HP-36 and BCP iodide 28 or alkyl iodide 29 towards the synthesis of HP-37 or HP-38. (A) Scope of BCPs compatible with the reaction with HP-36. (B) Scope of alkyl iodides compatible with the reaction with HP-36.

In this mechanism, a mediator such as (TMS)_3_SiOH 30 acts as the electron donor, donating an electron to the PC* and forming oxygen centred radical 31 and subsequently a silicon centred radical 32 following a radical Brook rearrangement.^85^32 can abstract the iodine (XAT) from 28 and generate the reactive BCP radical 33 along with iodo-silicon by-product 34 (Scheme 14). Radical 33 can then attack the DNA-conjugated alkene and close the photoredox cycle as before.

**Scheme 14:**
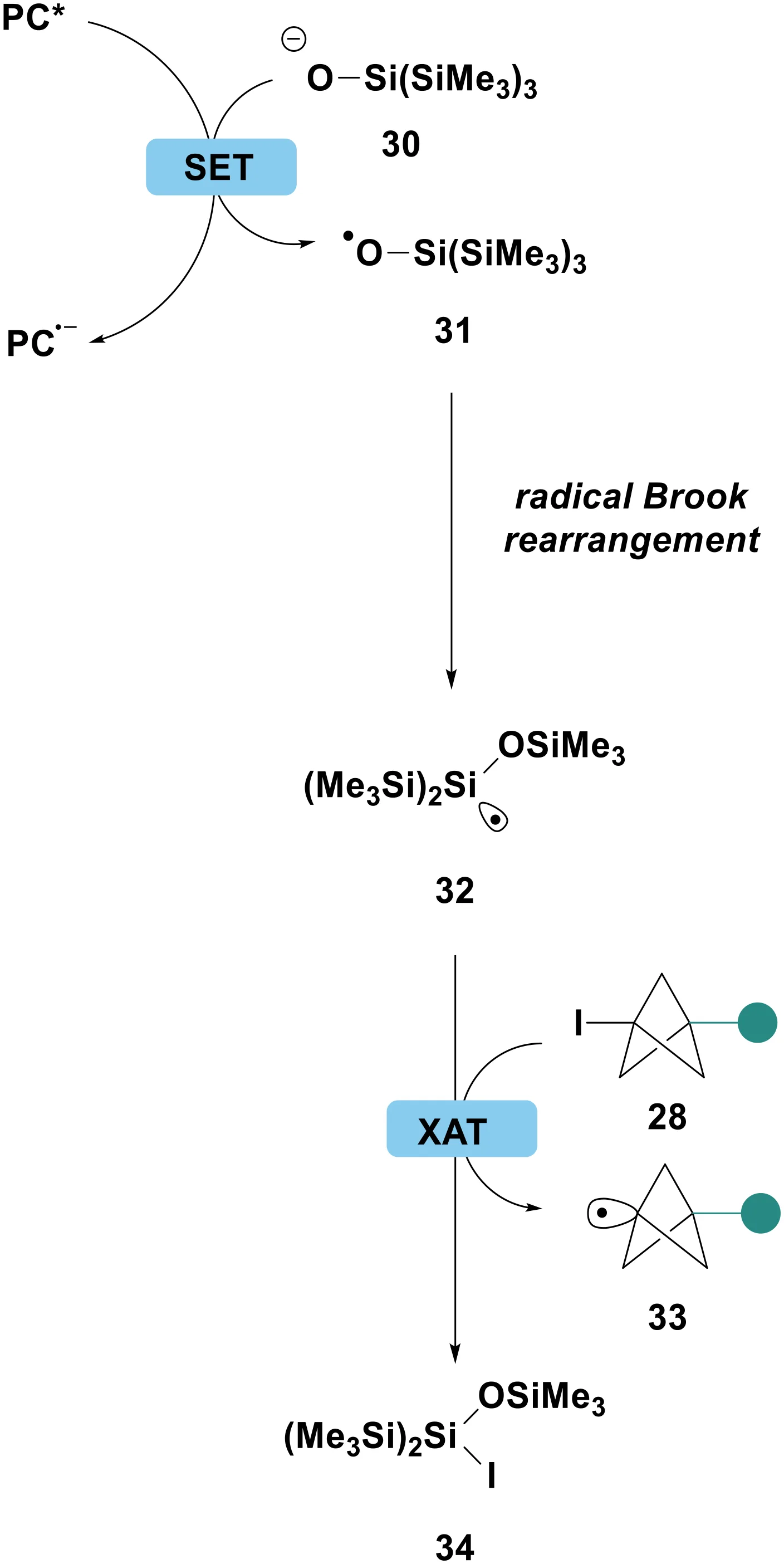
Mechanism by which (TMS)_3_SiOH assists in halogen abstraction and radical formation. (TMS)_3_SiOH 30 acts as an electron donor to form oxygen radical 31 which then undergoes a radical Brook rearrangement to form silicon radical 32 which can abstract a halogen from 28 to generate BCP radical 33 and iodo-silicon by-product 34.

In terms of the scope of the Giese reaction, amino esters (28a) and trifluoromethyl groups (28b) were both well tolerated while ketone (28c) only gave a moderate yield of 40% (Scheme 13).^61^ However, these moderate yields are still useful in terms of incorporation of these substrates into DELs. Aliphatic nitrogen heterocycles such as azetidine 28d were also amenable meanwhile amino BCP (28e) along with both benzylic (28f) and heteroaromatic (28g–h) BCP derivatives were accessible in moderate to good yield, allowing for the incorporation of privileged medicinal chemistry fragments.^61^ Finally, the reaction was further investigated with other non-BCP containing alkyl iodides and the reaction was seen to tolerate both linear (29a–b) and heterocyclic (29c–d) aliphatic amines.^61^ In the case of amino ester product 29a and azetidine product 29c, it is worth noting that the yields were improved in the presence of the BCP moiety (28a and 28d).

Alongside the Giese reaction, the same conditions were applied to *gem*-difluoroalkene synthesis (Scheme 15).^61^ Again, DNA-tethered trifluoromethylated alkene HP-39 was used and reacted with BCP iodide 28 to afford HP-40. Reaction with an amino ester containing BCP gave an excellent yield (28i) while an amino BCP gave a good yield (28j). When aromatic containing BCPs were screened, the benzylic BCP gave an excellent yield (28k), while a heteroaromatic BCP gave a moderate yield (28l).^61^ The incorporation of these BCPs into DEL is useful because they can serve as bioisosteres for *para*-substituted benzenes.

**Scheme 15:**
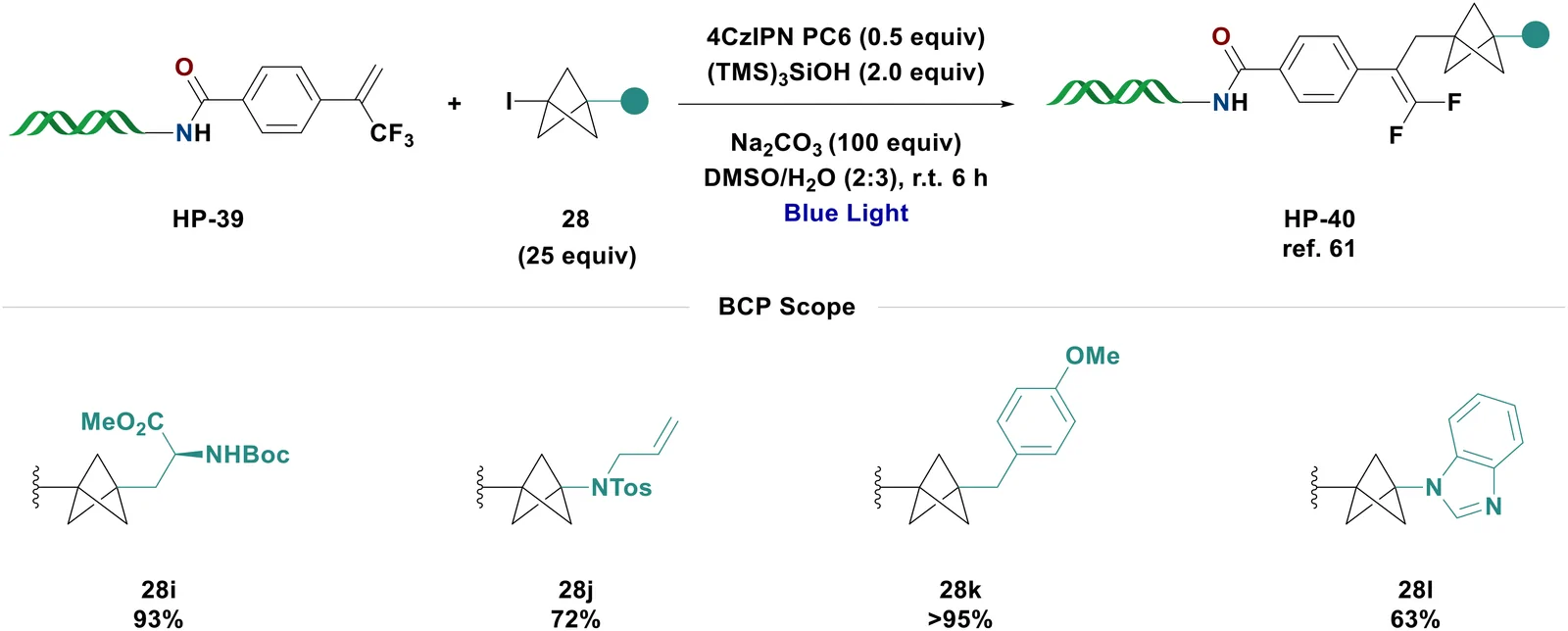
On-DNA reaction between DNA-conjugated alkene HP-39 and BCP iodides 28 towards the synthesis of *gem*-difluorinated alkene HP-40 along with the scope of BCPs compatible with the reaction.

Diazo compounds (35) were also shown to be useful radical precursors in on-DNA Giese reactions with DNA-conjugated styrenes (HP-41) to afford products of type HP-42 (Scheme 16A). In this reaction, a ruthenium photocatalyst PC5 was used, demonstrating how on-DNA photocatalysis is amenable to photocatalysts beyond iridium and 4CzIPN. Here, the excited Ru(ii)*, first oxidises the HE additive to generate a HE radical cation before reducing the diazo species 35 to facilitate the loss of nitrogen and generate the active radical species which can attack the styrene double bond and generate a second radical intermediate.^86^ This second radical species can abstract a hydrogen from the thiol additive to afford HP-42.^86^ The thiolate radical can then abstract a hydrogen from the Hantzsch ester (HE) radical cation to close the cycle.^86^ This reaction was limited to diazo species containing an electron-withdrawing group such as diazo esters (35a) and diazo amides (35b). Complex drug molecules such as paroxetine derivative 35c also performed well.^87^

**Scheme 16:**
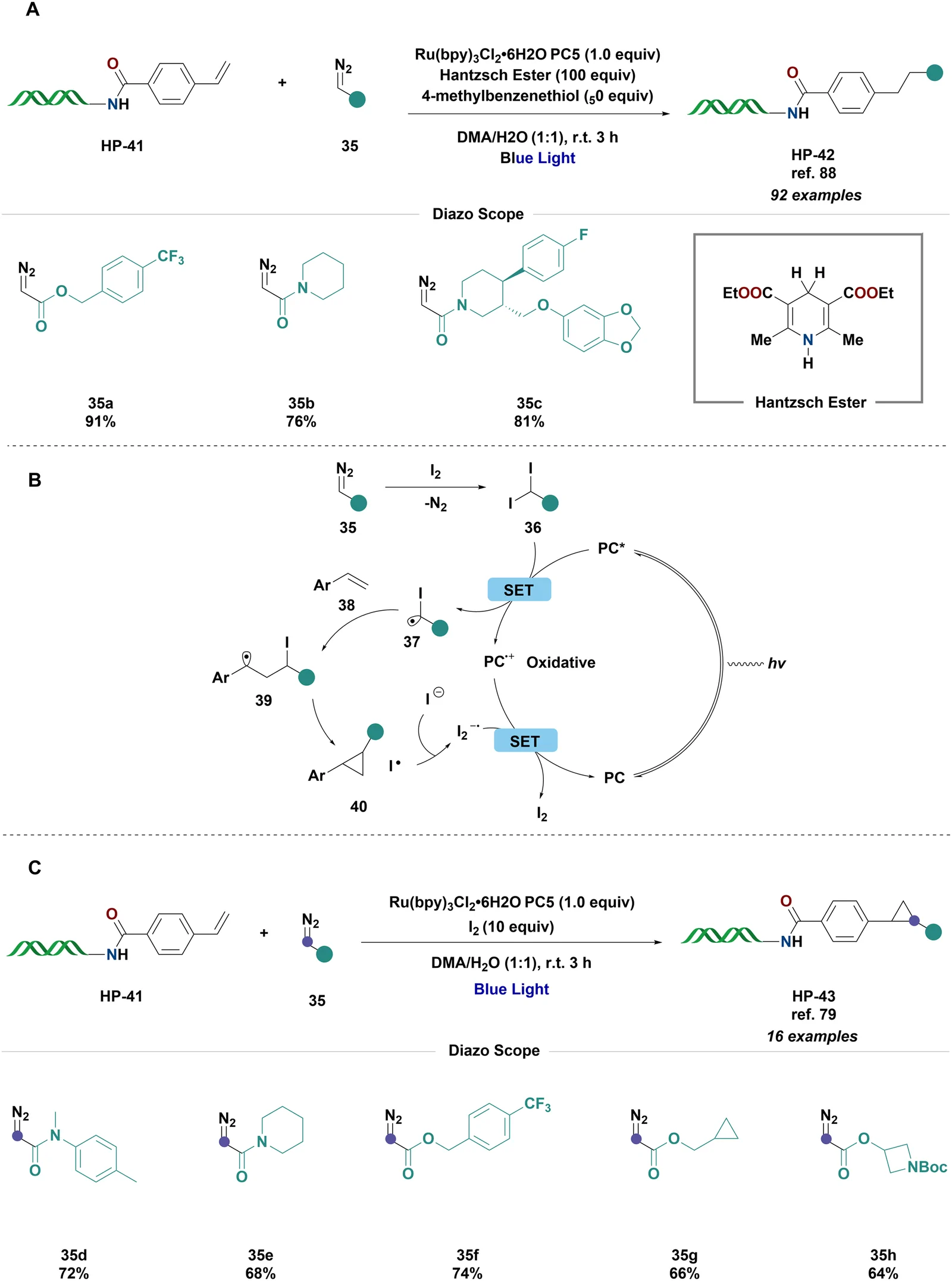
Reaction of diazo compounds 35 with DNA-conjugated alkenes HP-41. (A) Reaction between HP-41 and diazo species 35 in the presence of a Hantzsch ester and thiol to afford HP-42. Scope of diazo species tolerated in the reaction with HP-41 is shown. (B) Catalytic cycle that allows for the formation of cyclopropanated product HP-43 from diazo species 35. Upon reaction with iodine, diazo species forms di-iodo species 36. 36 can accept an electron from the excited PC* to form 37 which can capture aryl alkene 38 to form 39. 39 can then undergo a cyclisation process to eliminate an iodide radical and form product HP-43. The di-iodide radical anion formed can then donate an electron to the PC radical cation to return it to the ground state. (C) Cyclopropanation reaction between HP-41 and diazo species 35 in the presence of iodine to afford HP-43. Scope of diazo species tolerated in the reaction with HP-41 is shown.

While developing this reaction, Hu established that these substrates could also be effective at initiating on-DNA cyclopropanation in the presence of iodine.^87^ Driven by the loss of nitrogen gas, diazo species 35 can form diiodo intermediate 36 which can in turn be reduced by the ruthenium photocatalyst and form radical intermediate 37 along with an iodide anion (Scheme 16B).^88^ The radical species can then attack the DNA-tagged aryl alkene 38 to form a second radical intermediate 39 which can undergo radical cyclopropanation and release an iodine radical.^88^ This radical can combine with the iodide to form a diatomic iodine radical anion species. This donates an electron to the ruthenium radical cation to reform neutral iodine and the ground state PC.^88^

A range of diazo amides and esters were reacted with HP-41 under these reaction conditions to furnish cyclopropanated products of type HP-43 (Scheme 16C). Both aromatic (35d) and aliphatic (35e) amides were well tolerated as were benzylic (35f) and aliphatic (35g) esters including nitrogen heterocycles such as azetidine diazo ester (35h), a privileged medicinal chemistry fragment.^87^

While all the examples detailed so far have described the addition of a radical species to a DNA-conjugated alkene, Molander investigated the reverse process whereby a DNA-conjugate aryl iodide (HP-44) was used as the radical precursor (Scheme 17). In this case, an oxidative quenching process occurs here whereby the DNA-tagged iodo-arene HP-44 acts as the radical precursor and accepts an electron from the excited PC*.^89^ The resulting radical can attack the added alkene 41 to generate a second radical intermediate which can in turn abstract a hydrogen from the Hantzsch ester. The Hantzsch ester radical species formed from this can then donate an electron to the PC radical cation and close the catalytic cycle. Only five examples were reported in the study, but demonstrated that the reaction was amenable to a range of benzylic alkenes (41a–d) along with α,β-unsaturated carbonyl 41e.^90^ It is also worth noting the chemoselectivity of this reaction with the C–I bond being selectively reduced over the C–Cl bond.^90^

**Scheme 17:**
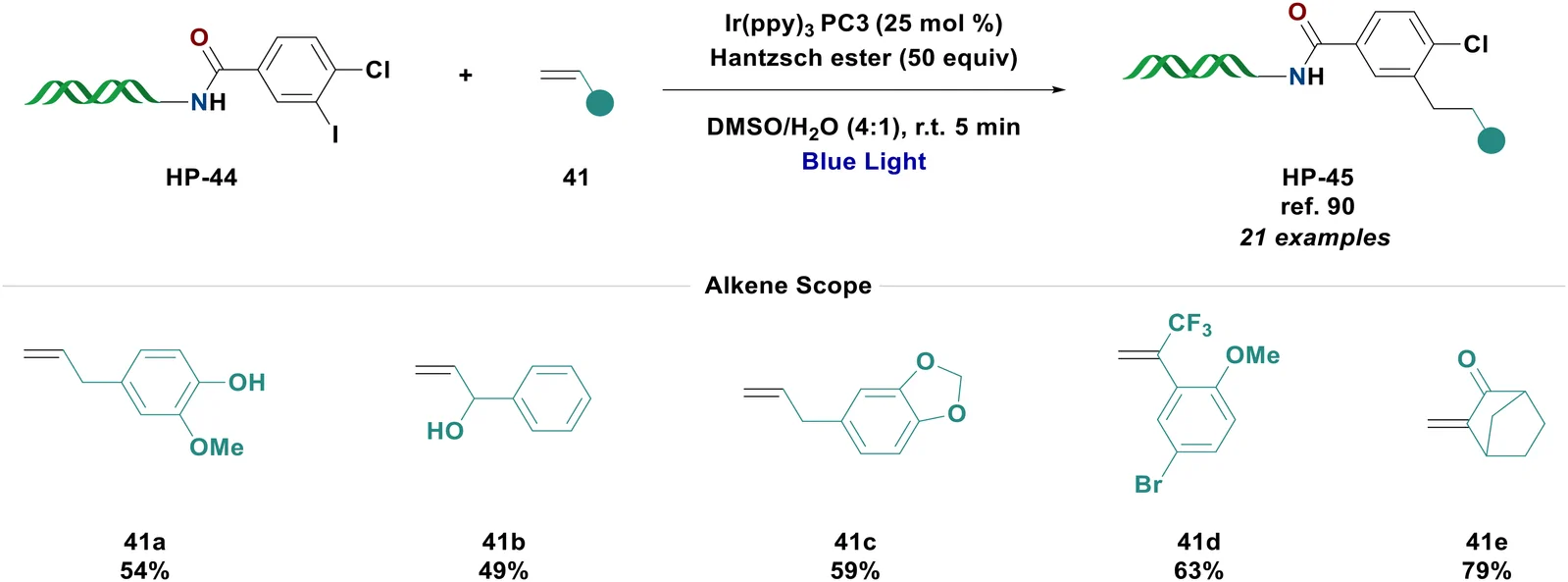
On-DNA reaction between DNA-conjugated aryl halide HP-44 and alkene 41 towards the synthesis of HP-45 along with the scope of alkenes compatible with the reaction.

Along with C–C bond formation, Giese-type reactions have also been used in the formation of C–S bonds. Hu reported a conjugate addition of thiols 42 to activated alkenes of type HP-46 using a ketone PC8 as the photocatalyst (Scheme 18).^91^ After single electron donation from the thiol to the activated ketone, the resulting thiolate radical anion can add across the alkene. However, unlike previous examples where the resulting alkyl radical is reduced and protonated, here the resulting alkyl radical captures oxygen to form a peroxide radical which undergoes three consecutive radical hydrogen abstractions to release water and add a hydroxy group to the α-position as seen in HP-47. The reaction was seen to tolerate thiophenol (42a) and heterocyclic derivative (42b) in excellent yield along with primary (42c), secondary (42d) and tertiary (42e) alkyl groups.^91^

**Scheme 18:**
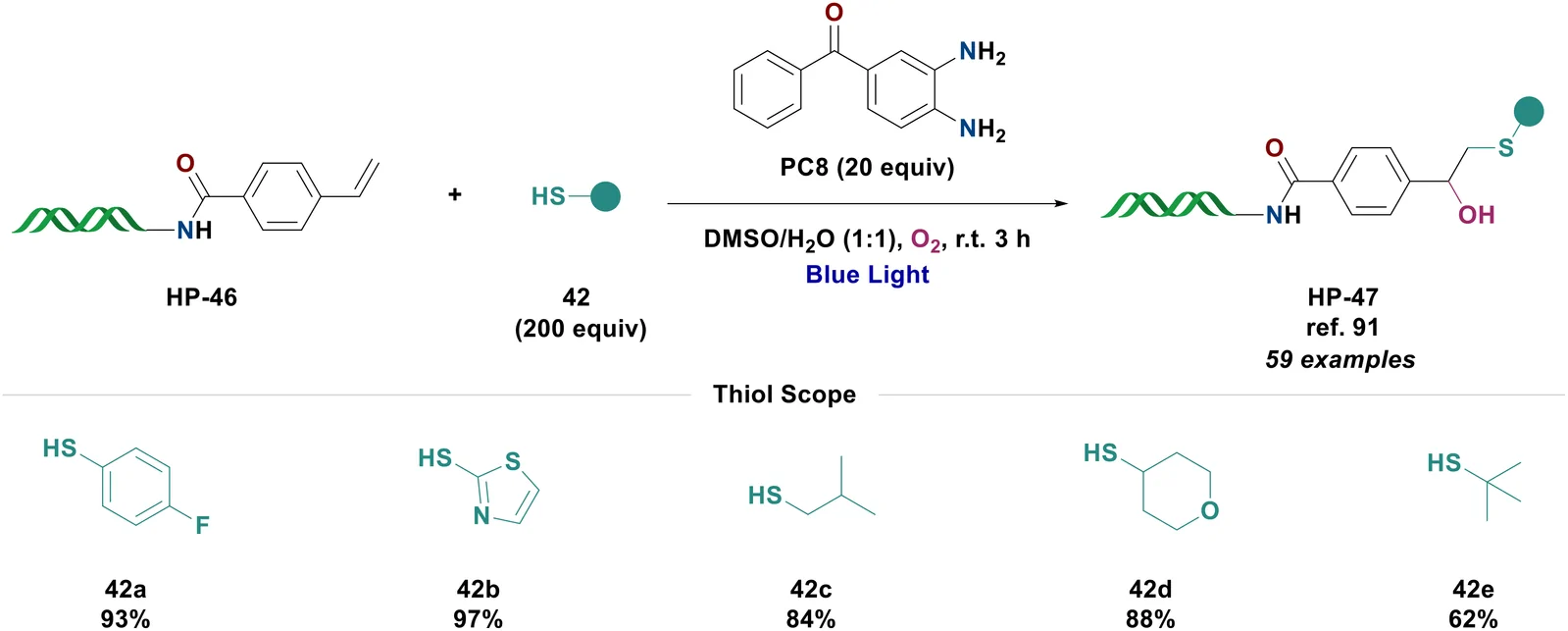
On-DNA reaction between DNA-conjugated alkene HP-46 and thiol 42 towards the synthesis of HP-47 along with the scope of thiols compatible with the reaction.

A similar on-DNA C–S bond forming reaction was proposed by Jia, demonstrating an on-DNA photoredox atom transfer radical addition (ATRA) reaction across an alkyne (HP-48) rather than an alkene as seen in most Giese reactions (Scheme 19).^92^ The model substrate used here was an ESAC-thiosulfonate 43 while the reaction was catalysed by eosin Y (PC9) with DMAP used as the initial photocatalyst quencher.^92^

**Scheme 19:**
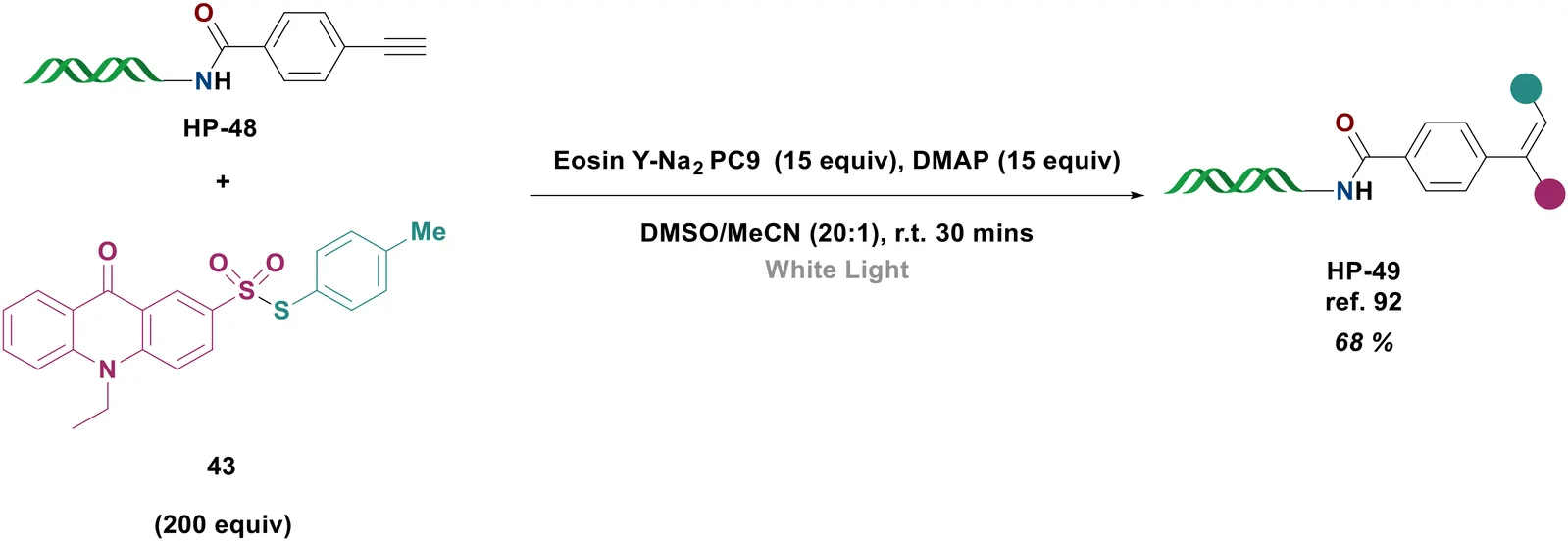
On-DNA ATRA reaction between DNA-conjugated alkyne HP-48 and ESAC-thiosulfonate 43 towards the synthesis of difunctionalised product HP-49.

Alongside alkenes, aldehydes are another potent radical coupling partner in photoredox chemistry. Researchers at WuXi AppTec developed a robust on-DNA synthesis of 1,2-amino alcohols from amino acid radical precursors (44) and aryl aldehydes (HP-50).^93^ Decarboxylation of the amino acid in a SET with the PC* generates an α-amino radical and PC radical anion in a reductive quenching pathway. The PC radical anion can then facilitate a second SET, donating an electron to the aldehyde to generate a second radical species which can undergo free radical–radical coupling with the α-amino radical to form the requisite 1,2-amino alcohol (HP-51) following protonation.^94^ A wide scope of aldehydes were compatible with these reaction conditions including both phenyl (HP-50a) and benzyl (HP-50b) derivatives (Scheme 20).^93^ Impressively, sterically hindered aldehydes such as HP-50c also gave good yields. Aryl groups bearing halogens (HP-50d) and electron-donating groups (HP-50e) demonstrated good to excellent reactivity while a range of privileged, heterocyclic aldehydes (HP-50f–h) were tolerated in moderate to good yield.^93^ The reaction scope was expanded to include ketones (HP-50i–l) although the reactivity of these substrates was diminished due to steric constraints.^93^ Nevertheless, these ketone substrates remain above the threshold for synthetic utility in DELs and therefore the amenability of this process to a wide range of carbonyls makes it ideal for expanding library sizes.

**Scheme 20:**
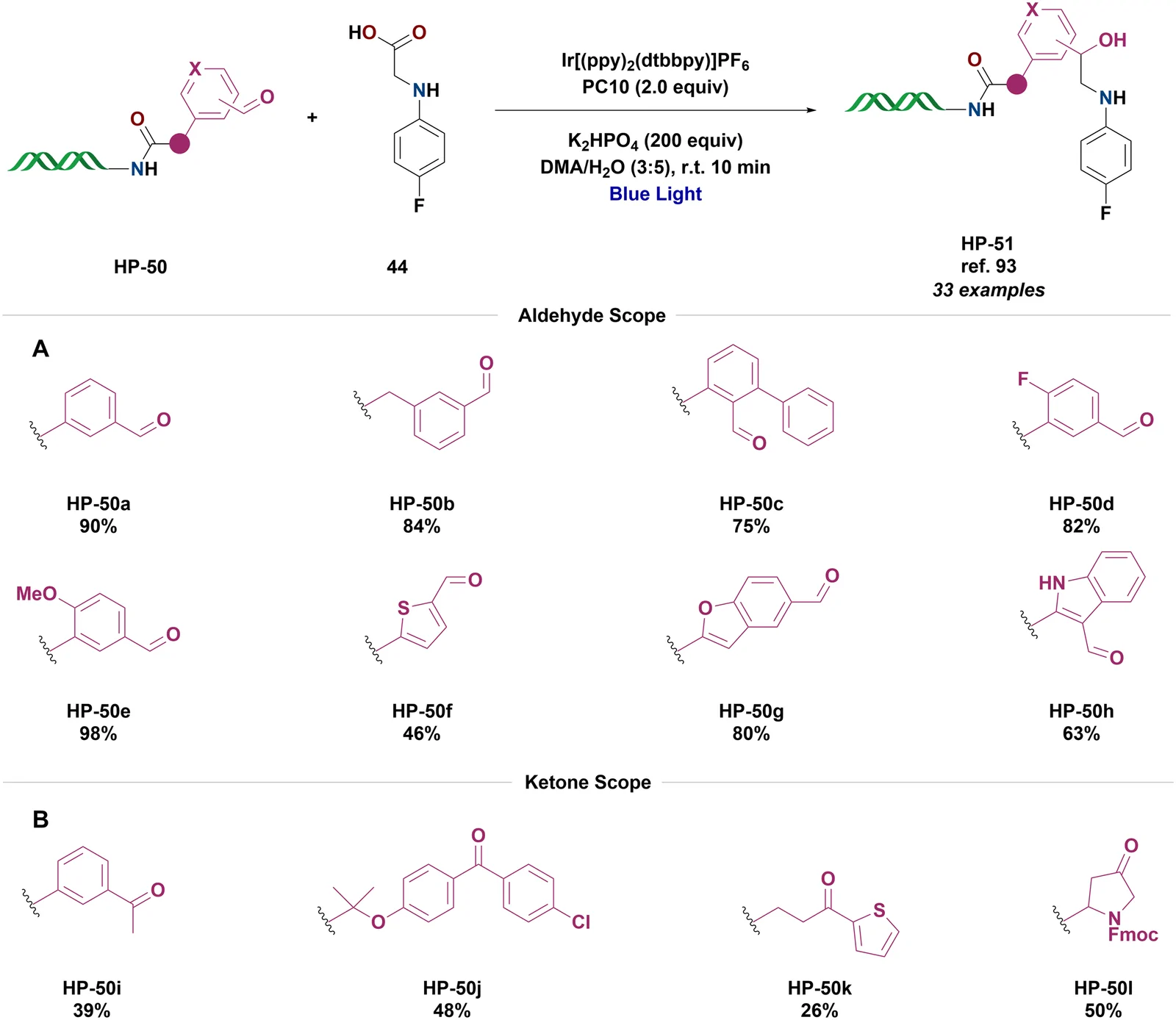
On-DNA carbonyl addition reaction between DNA-conjugated carbonyl HP-50 and carboxylic acid 44 towards the synthesis of HP-51. (A) Scope of DNA conjugated aldehydes compatible with the reaction with carboxylic acid 44. (B) Scope of DNA conjugated ketones compatible with the reaction with carboxylic acid 44.

Another class of photoredox reactions that has been successfully adapted towards on-DNA synthesis is the Minisci-type C–H functionalisation. The first example of this was reported by HitGen and involved the coupling of carboxylic acid radical precursors (45) with DNA-tagged *N*-methyl-quinoxalin-2-ones of type HP-52 (Scheme 21).^95^ Mechanistically, this proceeds in an oxidative quenching process to generate a radical intermediate which can attack the C=N bond and form a stabilised nitrogen centred radical.^96^ This resulting radical can then reduce the oxidised PC back to the ground state and upon loss of a proton, form the C–H functionalised product HP-53. In this on-DNA process, amino acids 45a–c were incorporated when an iridium photocatalyst was utilised. In the presence of 4CzIPN, α-oxy carboxylic acids (45d) and aliphatic carboxylic acids (45e–f) and privileged heterocyclic moieties (45g–h) could also be incorporated into the reaction scope.^95^

**Scheme 21:**
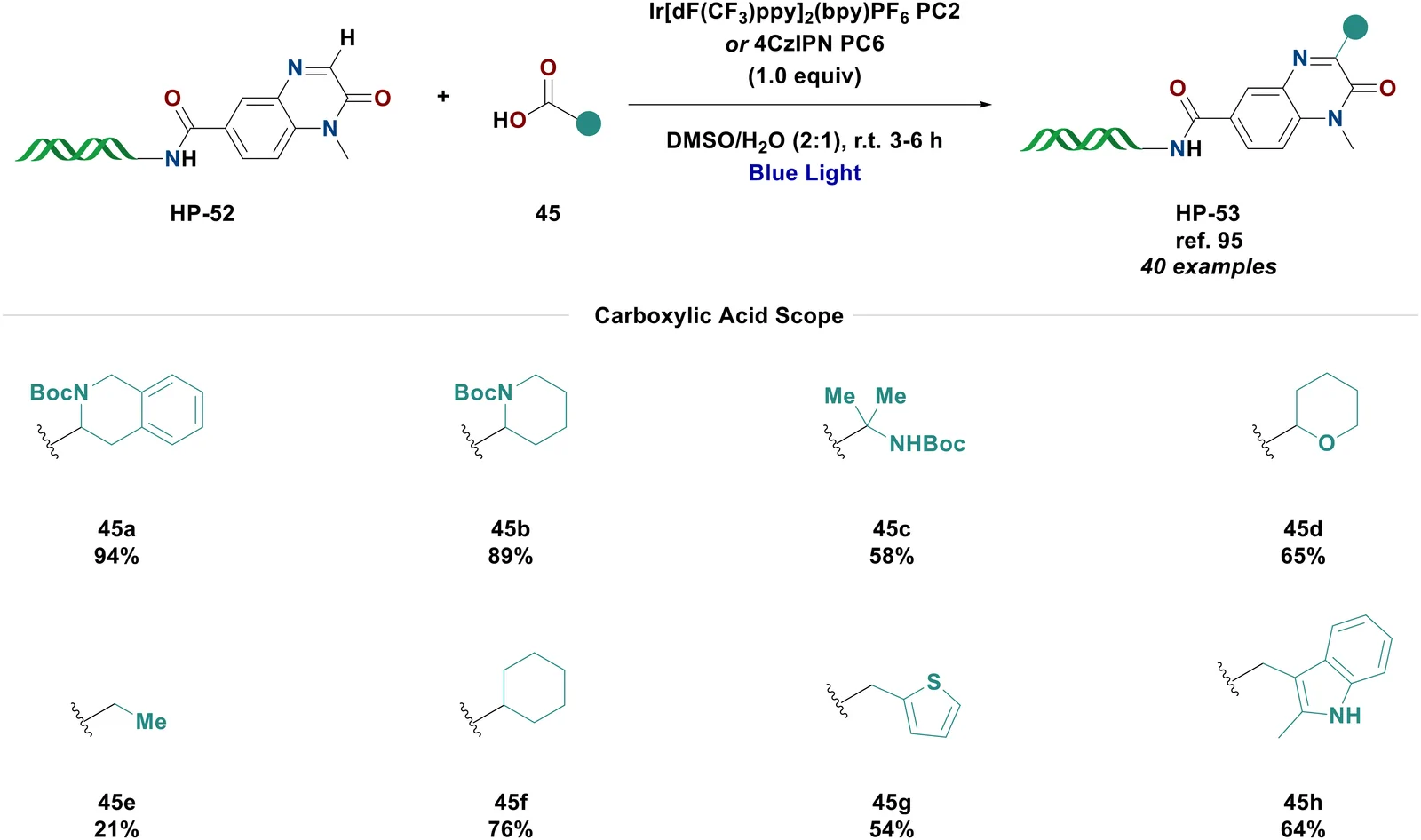
On-DNA Minisci reaction between DNA heterocycle HP-52 and carboxylic acid 45 towards the synthesis of HP-53 along with the scope of carboxylic acids compatible with the reaction.

Kim similarly used DHP-derived radical precursors 46 instead of carboxylic acids in order to install a range of amino ester groups to HP-54.^97^ In this case, a reductive quenching process occurred and Na_2_S_2_O_8_ was required as a sacrificial oxidant to afford products of type HP-55 (Scheme 22).^97^

**Scheme 22:**
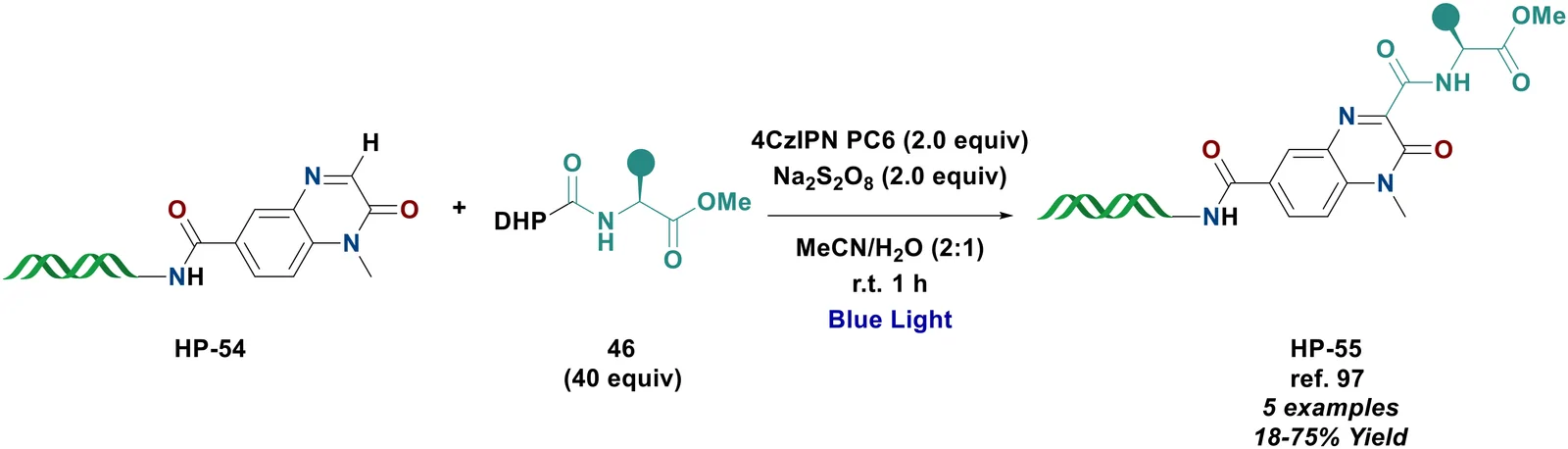
On-DNA Minisci reaction between DNA heterocycle HP-54 and DHP derivative 46 towards the synthesis of HP-55.

Molander has also explored the application of Minisci reactions in the context of on-DNA chemistry through their development of a C–H arylation process using a range of heteroarenes 47 which were coupled to DNA-tethered aryl halides HP-56 to afford products of type HP-57 (Scheme 23).^98^ In terms of the mechanism, this reaction is similar to the work of Kim *et al.* (HitGen) in that an oxidative quenching pathway is followed and a heteroatom-centred radical is initially formed which is key to the reaction's success.^95,97^ The main difference here is the inclusion of DIPEA which acts to facilitate deprotonation in the final step and therefore aid in the reduction of the PC radical cation to the ground state.^98^ This work exemplifies a vast scope with a range of privileged nitrogen-containing heterocycles amenable to the reaction conditions. These include quinolines (47a), isoquinolines (47b), pyrroles (47c), indoles (47d), pyrimidines (47e) and pyrazines (47f) with the main exclusion being pyridines which were not included in the scope due to their poor radical accepting abilities.^98^ Sulfur-containing heterocycles such as thiophene (47g) were also tolerated, as were biologically active molecules such as caffeine (47h). However, while the reaction is broad in scope, it can only achieve moderate to good yields with only two examples above 80% reported.^98^ It is worth noting, however, that this reaction is seen to be selective towards C–I functionalisation over C–F functionalisation. This selectivity was seen further when the scope of the headpiece was examined with heterocycle 48. Selective C–I functionalisation was seen to prevail even in the presence of a C–Cl bond (HP-58) while sterics were not seen to be an issue with the more hindered *ortho* position allowing functionalisation in HP-59. Along with aryl iodides, aryl bromides were also demonstrated to be successful coupling partners (HP-60, HP-61 and HP-62). In the case of HP-60, the unreactive nature of the pyridine group could be exploited to allow incorporation of a nitrogen heterocycle into the aryl halide structure while preventing any homo-coupling. Again, selective functionalisation was observed with C–Br functionalisation prevailing over C–F functionalisation.

**Scheme 23:**
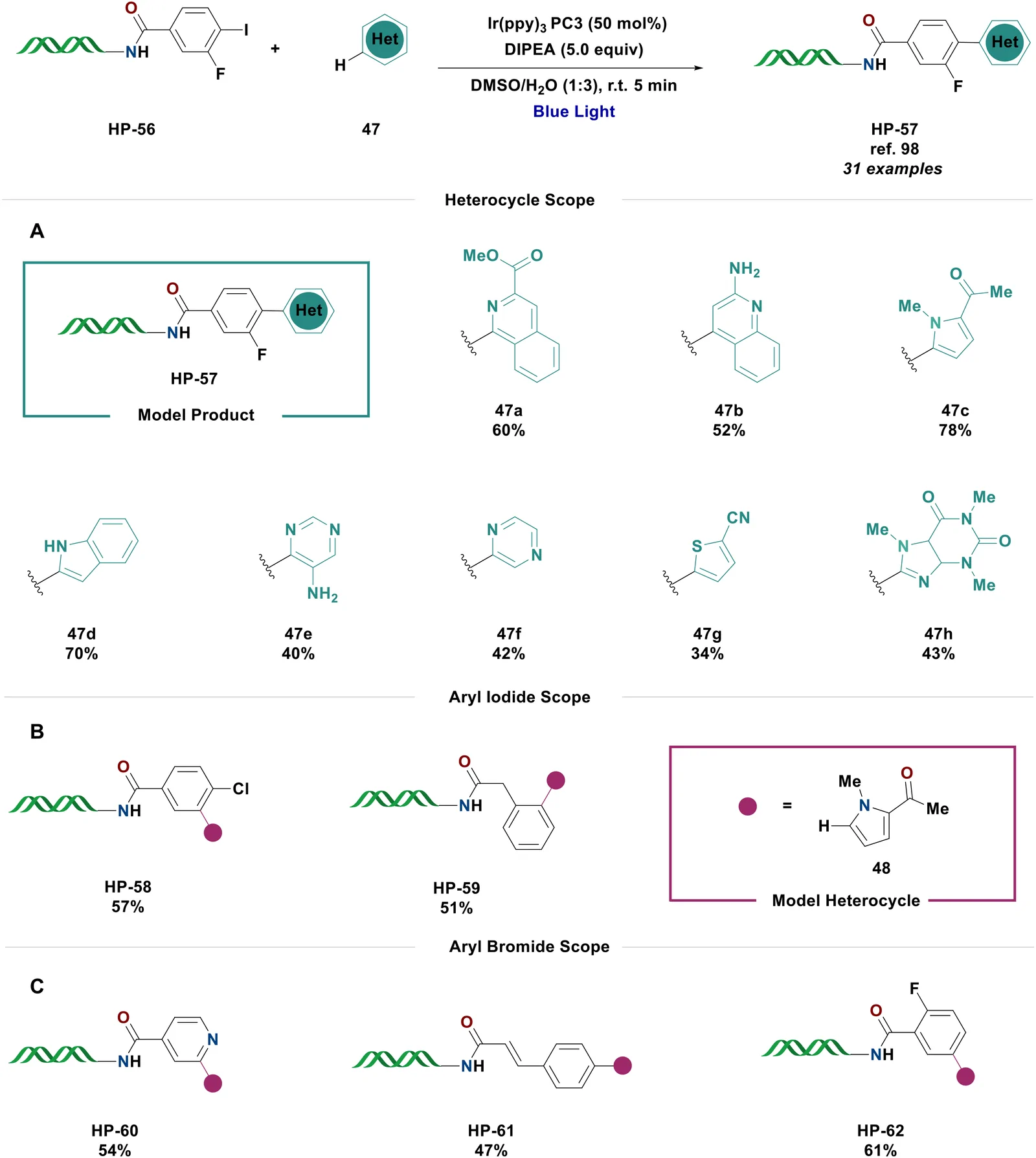
On-DNA Minisci reaction between DNA aryl-halide HP-56 and heterocycle 47 towards the synthesis of HP-57. (A) Scope of heterocycles compatible with the reaction with HP-56. (B) Scope of DNA-conjugated aryl iodides compatible with the reaction with heterocycle 48. (C) Scope of DNA-conjugated aryl bromides compatible with the reaction with heterocycle 48.

### 3.2 Hydrogen atom transfer (HAT) – photoredox

Another class of photoredox reactions is the hydrogen-atom transfer (HAT) photoredox coupling whereby the radical intermediate is formed by the abstraction of a hydrogen atom rather than from the elimination of a radical precursor. The radical forming HAT process can either be carried out by the PC directly or by a dedicated HAT catalyst. This mode of activation is desirable as it circumvents the need for pre-functionalisation, however, it is significantly more challenging as regioselectivity needs to be carefully controlled. These HAT photoredox processes have been adapted towards on-DNA synthesis, and to date, have once again focused on expanding routes towards Giese-type reactions. The first example of an on-DNA HAT reaction was performed by researchers at Hitgen and involved a DNA-conjugated Michael acceptor HP-63 (Scheme 24).^99^ Here, the HAT event was carried out directly by the PC in a classical reductive quenching pathway as commonly seen for Giese reactions. In order to overcome the challenges surrounding regioselectivity and to facilitate the formation of a stabilised radical, tertiary amines bearing an aryl substituent (49) were chosen as a suitable coupling partner to form product HP-64.^99^ Diphenyl amines (49a), aliphatic heterocycles (49b) and dimethyl amines (49c) were all tolerated.^99^ However, because amine and alkene substrates were explored simultaneously, rather than benchmarking a broad amine selection against a single DNA-conjugated alkene, the generality of the amine scope remains difficult to assess. This suggests that the reaction may only tolerate a small window of conditions and coupling partners. The scope of alkenes was more systematically explored across a range of substituted Michael acceptors such as HP-65 while maintaining high conversion.^99^ Similarly, styrene derivatives (HP-66) performed comparatively well.

**Scheme 24:**
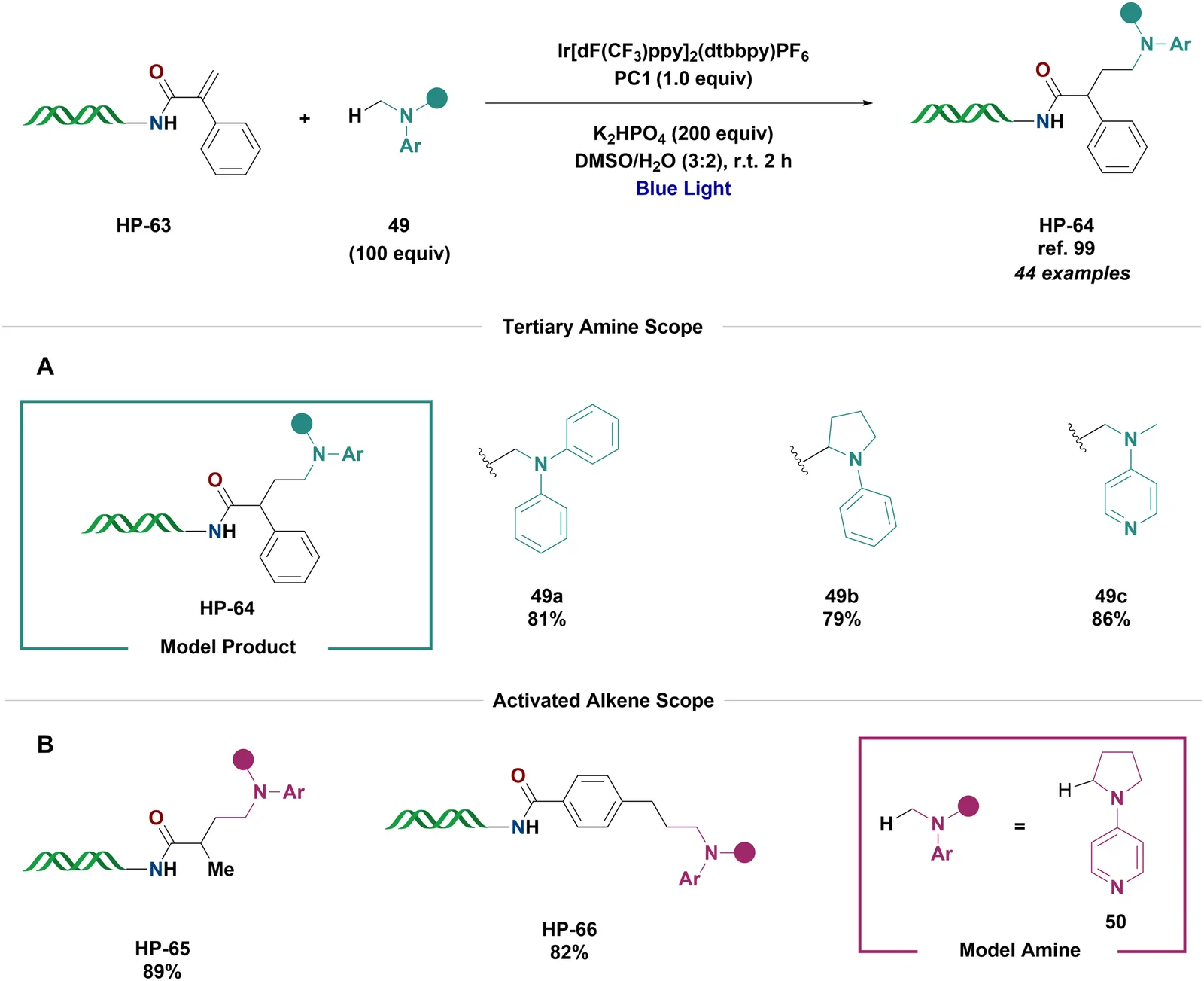
On-DNA Giese reaction between DNA-conjugated alkene HP-63 and aryl amines 49 towards the synthesis of HP-64. (A) Scope of aryl amines compatible with the reaction with HP-63. (B) Scope of DNA conjugated alkenes compatible with the reaction with amine 50.

Around the same time, Lu described the HAT-activated Giese reaction with tertiary amines.^100^ However, this work demonstrated the first utility of a HAT catalyst in on-DNA synthesis. In this case the HAT catalyst used was quinuclidine. Quinuclidine acts to donate an electron to the PC* in a reductive quenching pathway, forming a PC radical anion species and a quinuclidine radical cation 51 (Scheme 25).^100^ This quinuclidine radical cation can then carry out the HAT process and abstract a hydrogen from the amine 52 forming an α-amino radical 53 and an ammonium quinuclidine species 54 which can regenerate the quinuclidine HAT catalyst upon loss of a proton.^100^ The amino radical can then react with the Michael acceptor HP-67 in a Giese fashion to generate a second radical intermediate HP-68 which can accept an electron from the PC radical anion, reforming the ground state PC and the functionalised product HP-69 upon protonation.

**Scheme 25:**
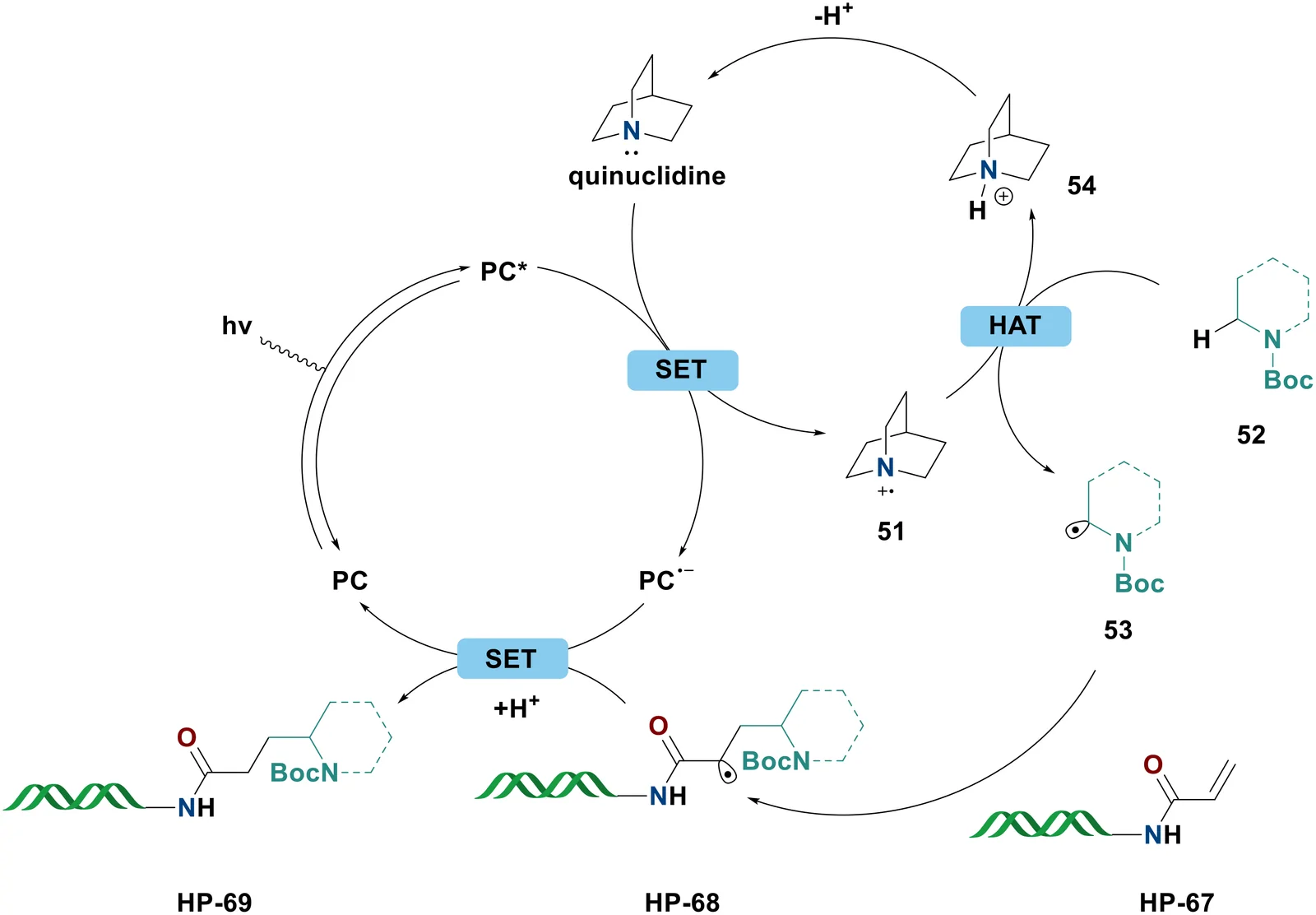
Dual catalytic cycle of photoredox HAT reaction involving a PC and HAT catalyst. Excited PC* accepts an electron from quinuclidine to form radical cation species 51 which can abstract a hydrogen from amine 52 to form 53. 53 can then capture alkene HP-67 to form HP-68 which can then accept an electron from the PC radical anion to form product HP-69.

In terms of the scope, this study focused on exploring privileged medicinal chemistry fragments such as aliphatic nitrogen heterocycles (Scheme 26). Five membered heterocycles (52a) were seen to be well tolerated including fused bicyclic heterocycles (52b).^100^ Six membered heterocycles (52c) were less well tolerated with poor to moderate yields observed. The inclusion of a 4-acetyl group (52d) acted to improve the yield but conversely, the installation of an aryl group (52e) or bromo group (52f) in this position resulted in diminished yields.^100^ Impressively, however, spirocyclic structure 52g, an increasingly popular motif in drug discovery, was amenable to the reaction conditions. Finally, expansion to a seven-membered heterocycle resulted in a good yield of the desired product (52h).^100^ The reason for the lower yields seen here could be attributed to a side reaction with quinuclidine. With the quinuclidine also being a tertiary amine and also being in vast excess, it can abstract a hydrogen from itself and generate a quinuclidine radical which can also undergo Giese addition to form a side product.^100^ Despite this, many of the products described here pose as promising candidates for DEL inclusion following Boc deprotection.^101^

**Scheme 26:**
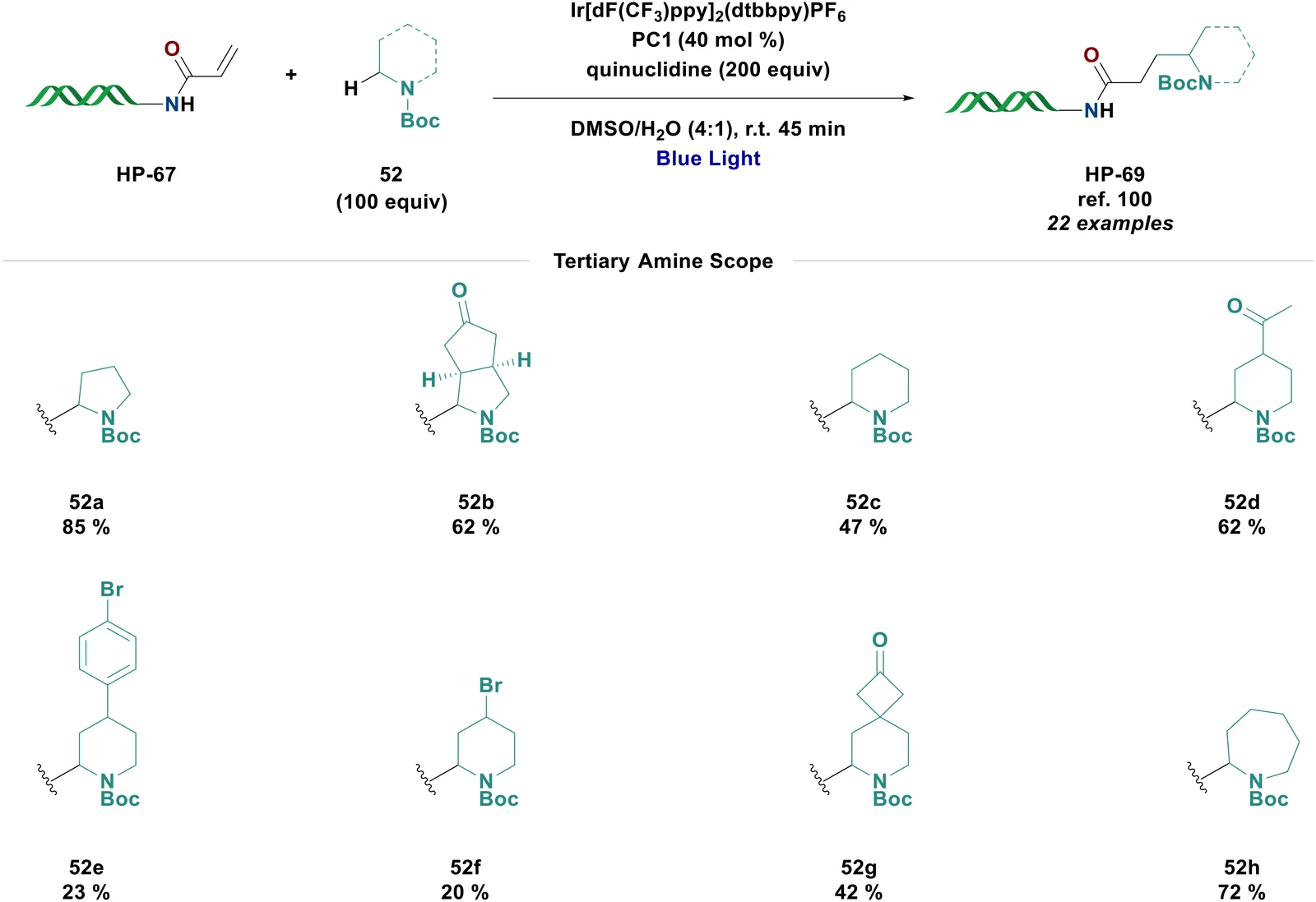
On-DNA Giese reaction between DNA-conjugated alkene HP-67 and tertiary amine 52 towards the synthesis of HP-69 along with the scope of tertiary amines compatible with the reaction.

This method of using a quinuclidine HAT catalyst for on-DNA Giese-type reactions was expanded by Hili who demonstrated that the reactants could be reversed by using a DNA-tethered amine and coupling it with a range of styrene derivatives (Scheme 27).^102^ Interestingly, they were also able to expand the scope to include secondary amines such as substrate HP-70 using this method by employing DNA-conjugated phenyl amines to assist in radical stabilisation. Keeping the alkene off-DNA avoided issues with the quinuclidine-mediated Giese-addition side product as it was removed during workup. Using substrate HP-70, a range of styrene derivatives were explored. Styrenes bearing electron-withdrawing groups (55a–b) were seen to promote reactivity while those with electron-donating groups (55c) reacted poorly. Along with styrenes, vinyl heterocycles (55d–e) were seen to be amenable to the reaction conditions.^102^

**Scheme 27:**
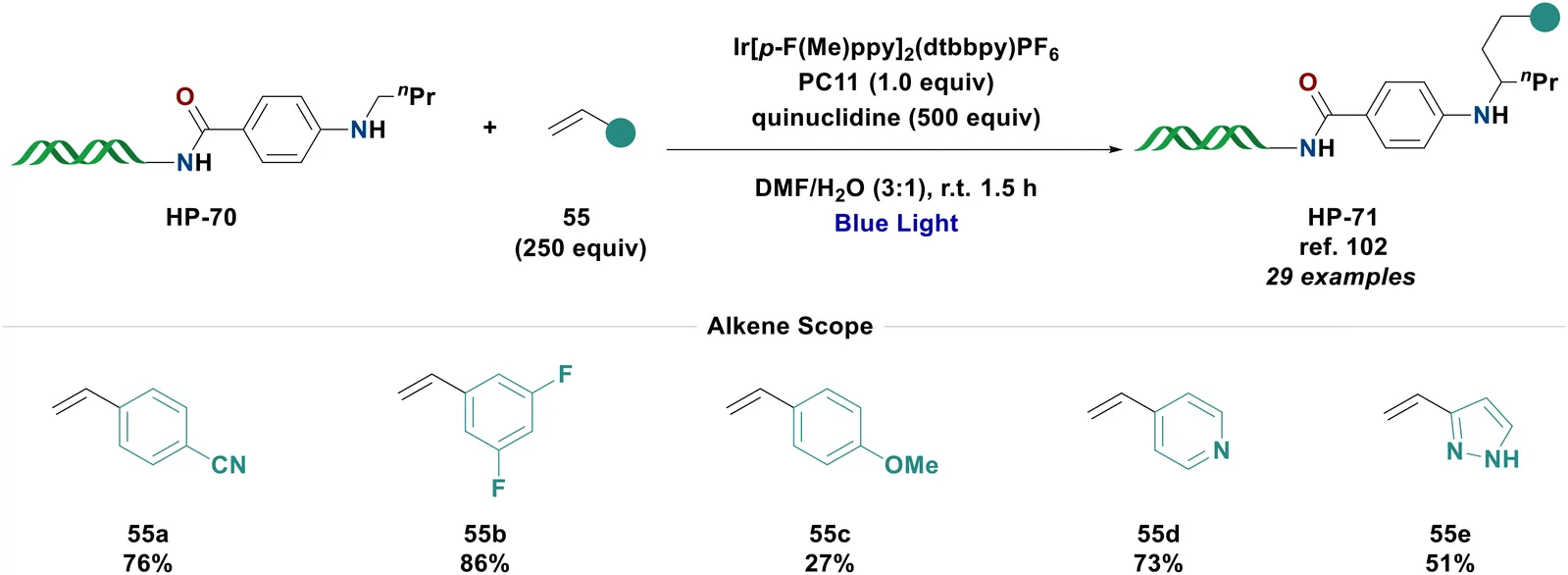
On-DNA Giese reaction between DNA-conjugated amine HP-70 and alkene 55 towards the synthesis of HP-71 along with the scope of alkenes compatible with the reaction.

### 3.3 Metallaphotoredox

Beyond standalone photoredox, integrating a transition-metal catalyst gives metallaphotoredox platforms that enable otherwise inaccessible transformations under mild and DNA-compatible conditions. The field of metallaphotoredox catalysis is vast,^103–105^ and in the context of on-DNA chemistry, nickel–photoredox systems have proven synthetically powerful (Scheme 28). In a typical nickel–photoredox catalytic cycle, a PC is excited to PC* which subsequently undergoes reductive quenching in the presence of a radical precursor (RP) molecule 56 to generate a PC radical anion and a radical intermediate 57. Rather than directly reacting with a second coupling partner, this radical is instead captured by an active Ni^0^ catalyst 58 to form a Ni^I^ species 59. Oxidative addition of a coupling partner such as an aryl halide 60 subsequently occurs to form Ni^III^ intermediate 61 which can then release the functionalised product 62 in a reductive elimination. This leaves Ni^I^ species 63 which can accept an electron from the PC radical anion thus regenerating both the active Ni^0^ catalyst and the ground state PC.

**Scheme 28:**
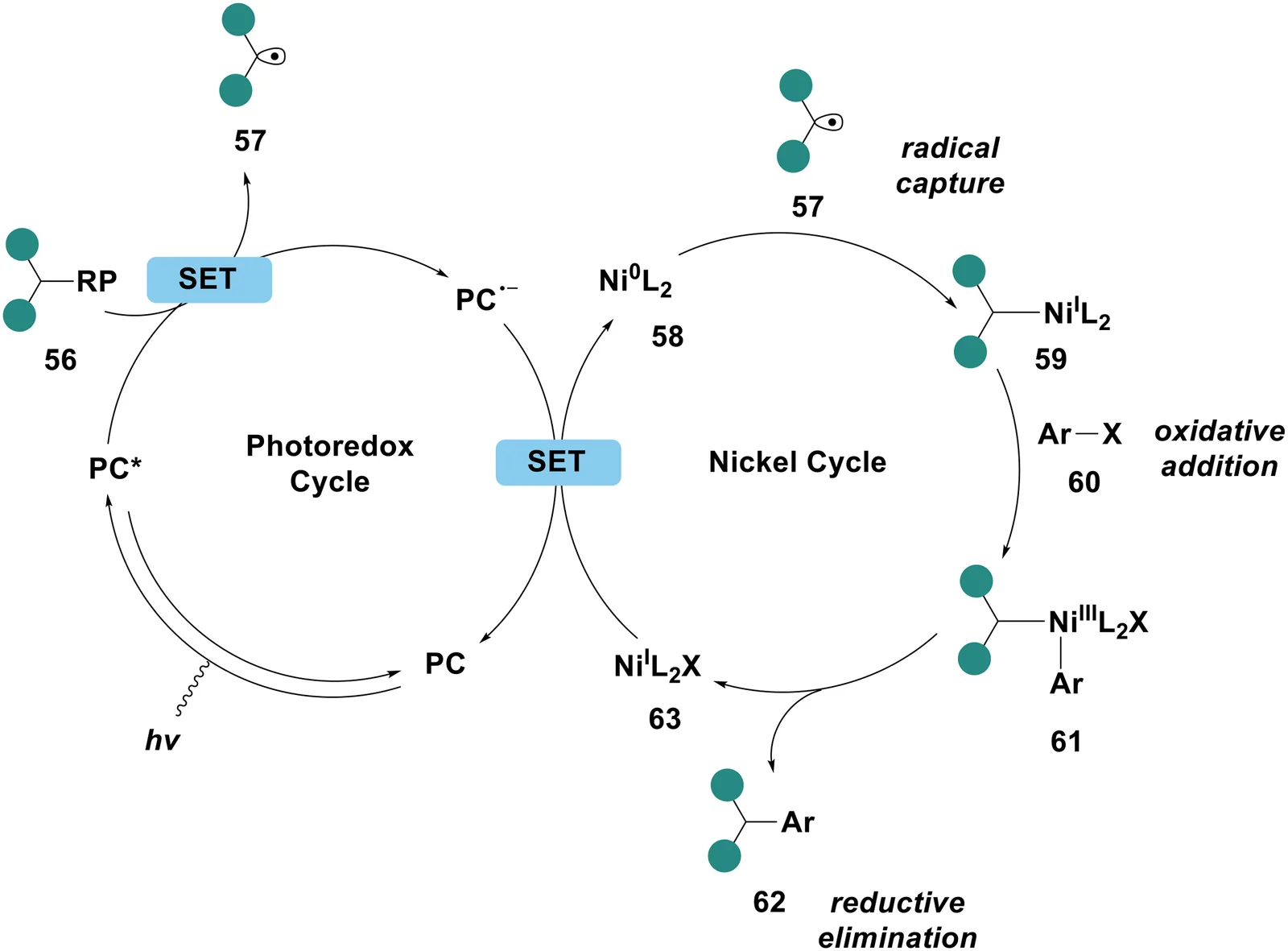
Metallaphotoredox catalytic cycle whereby the PC is coupled to a nickel catalyst. Excited PC* can accept an electron from radical precursor 56 to form radical 57. 57 can coordinate to Ni^0^ to form Ni^I^ species 59. Oxidative addition of 60 forms Ni^III^ species 61 which can undergo reductive elimination to afford 62. Ni^I^ is then reduced back to Ni^0^ by the PC radical anion.

The utility of this methodology for on-DNA synthesis was pioneered by Flanagan (Pfizer) and Molander. In the work by Flanagan, a DNA-tagged aryl iodide (HP-72) was coupled to alkyl radicals derived from amino acid 64 and bromide 65 radical precursors to afford products of type HP-73 (Scheme 29).^106,107^ When exploring amino acid precursors, tertiary amino radicals (64a) performed best under the reaction conditions. This included privileged aliphatic heterocycles (64b), bridged bicyclic heterocycles (64c) and spirocycles (64d).^106^ Secondary amino radicals (64e) were also amenable in moderate yield.^106^ The yield of alkyl bromides was generally lower, albeit still above the threshold for DEL inclusion. Simple linear (65a) and cyclic (65b) alkyl bromides gave good yields, however, the inclusion of an amino group (65c–d) or spirocycles (65e) saw a reduction to the yield.^107^ In the case of 65d, the yield of product derived from the alkyl bromide was significantly lower than when the corresponding amino acid was used to prepare the same product (64b). It is worth noting that silanol was an important inclusion in the alkyl bromide reaction. As seen before (*vide supra*, Scheme 14), upon oxidation, the silanol forms a silicon-centred radical which assists in the abstraction of the bromide to form the alkyl radical.^107^

**Scheme 29:**
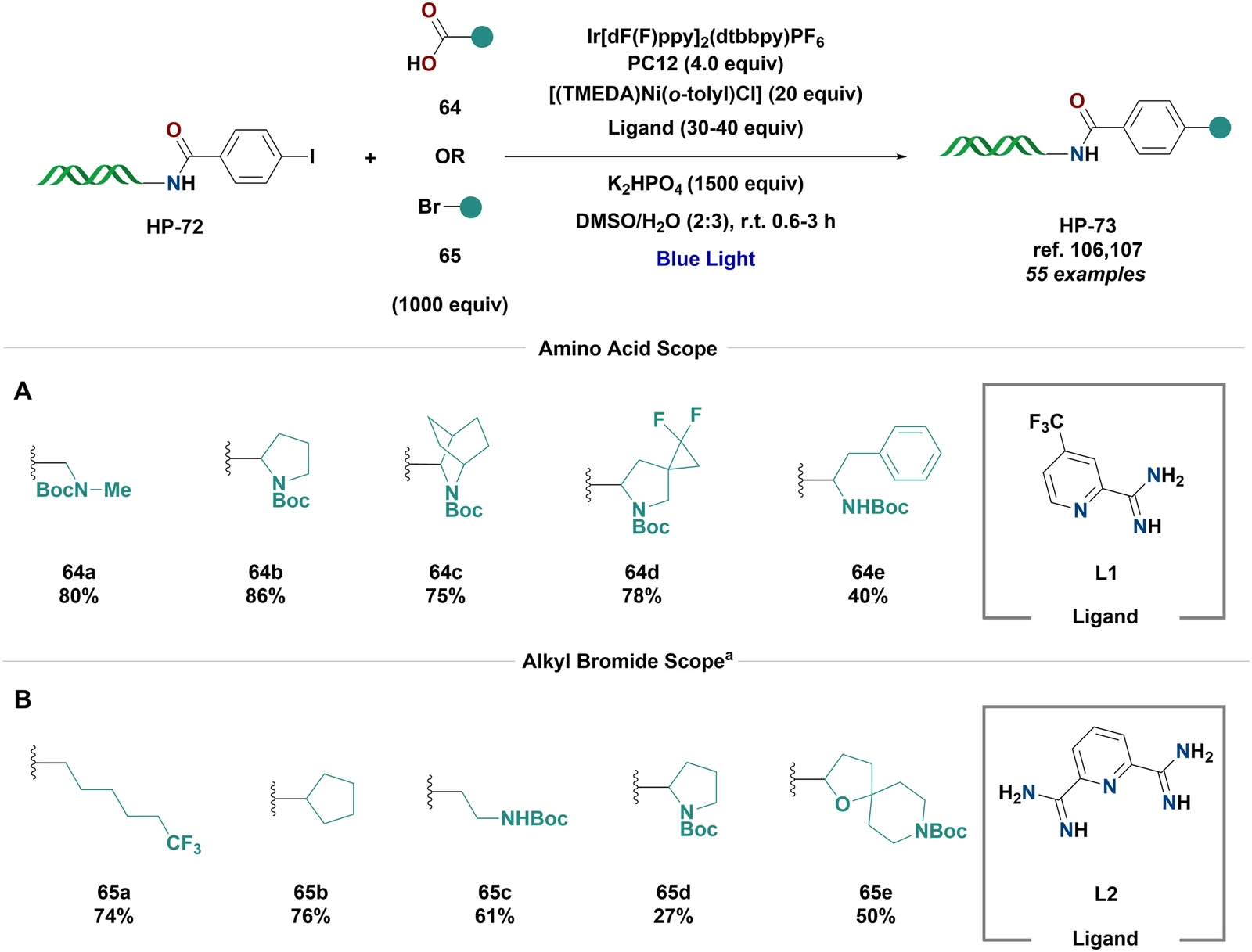
On-DNA aryl–alkyl coupling reaction between DNA-conjugated aryl iodide HP-72 and carboxylic acid 64 or alkyl bromide 65 towards the synthesis of HP-73. (A) Scope of carboxylic acids compatible with the reaction with HP-72 and L1. (B) Scope of alkyl bromides compatible with the reaction with HP-72 and L2. ^a^Silanol (400 equiv.) was also included for alkyl bromides.

Simultaneously, Molander developed a versatile approach towards C(sp^2^)–C(sp^3^) cross-coupling on-DNA, utilising a wide range of radical precursors (66) including carboxylic acids (67), DHP derivatives (68), α-silylamines (69) and alkyl bromides (70).^28,84^ Amino acids, α-silylamines and alkyl bromides worked best with iridium-based PCs meanwhile DHP derivatives employed 4CzIPN (PC6) (Scheme 30). The coupling of amino radicals to DNA-conjugated aryl halides (HP-74) here was seen to be less effective than demonstrated in the Pfizer report, with only moderate to good yields observed across a range of tertiary (67a–b) and secondary (67c) amino radicals.^28^ In the case of 67a, 75% yield was seen here compared to 86% obtained by Pfizer (64b). This could suggest that the nickel catalytic system used by Pfizer was more effective in inducing cross coupling. When α-silylamines were used as the radical precursor, the scope was limited to α-amino radicals (68a–c).^84^ When DHP-derived radicals were utilised, aryl bromides performed better than aryl iodides. Secondary alkyl radicals (69a) coupled effectively while benzyl radical (69b) and α-alkoxy radical (69c) were also tolerated.^28^ Finally, alkyl bromides were the most explored class of radical precursor. Here, triethylamine was included to act as a reductant and drive the PC towards its active radical anion state while magnesium chloride was also utilised to help stabilise the DNA backbone.^84^ Heterocyclic groups (70a–b) were seen to be amenable with larger, less strained ring systems giving more favourable yields.^84^ Linear alkyl bromides were also tolerated including those bearing a terminal chloride (70c). With 70c, it is worth noting the high selectivity towards functionalisation through the bromide over the chloride.^84^

**Scheme 30:**
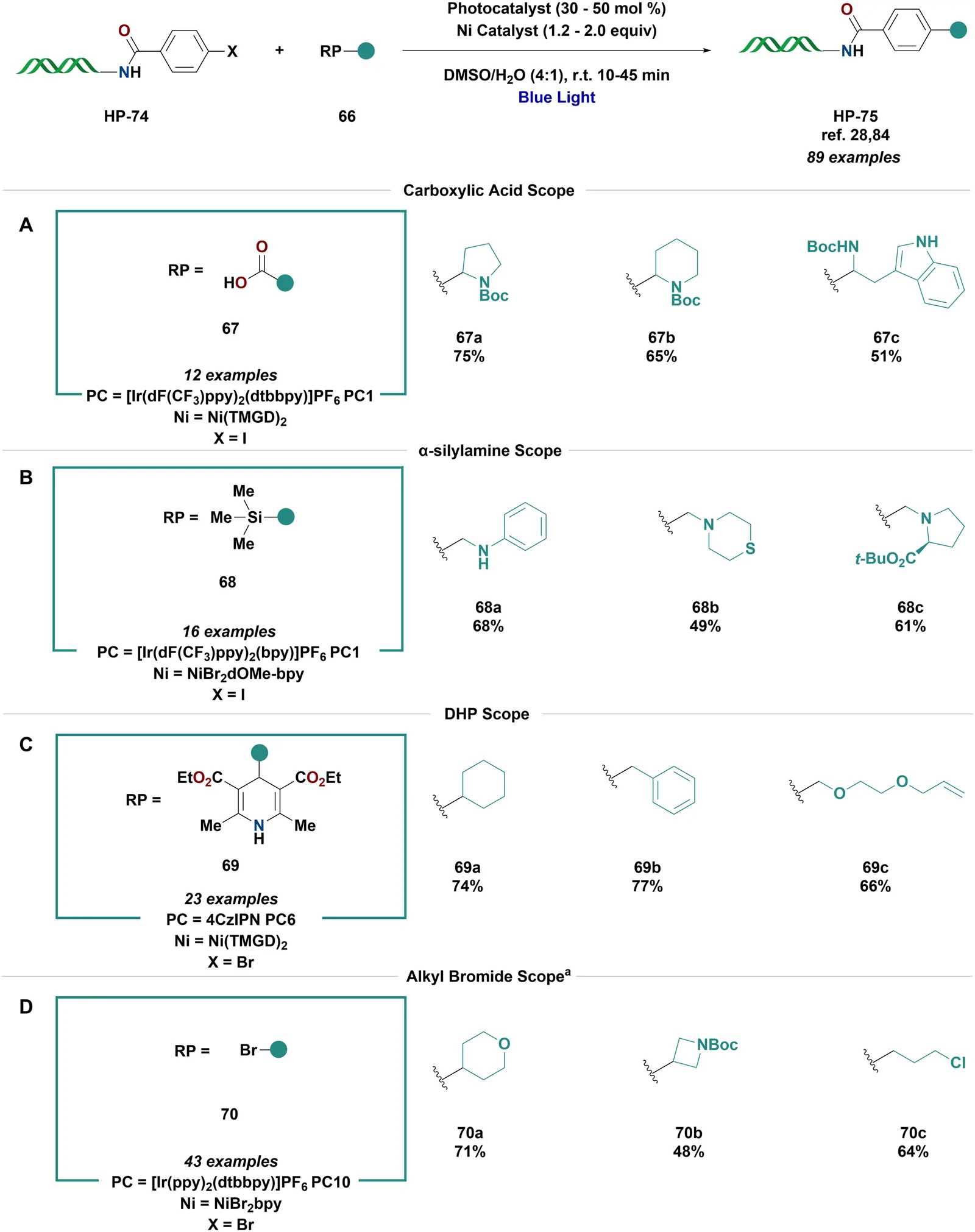
On-DNA reaction between DNA-conjugated aryl halide HP-74 and various radical precursors 66 towards the synthesis of HP-75. (A) Scope of carboxylic acids (67) compatible with the reaction. (B) Scope of α-silylamines (68) compatible with the reaction. (C) Scope of DHP derivatives (69) compatible with the reaction. (D) Scope of alkyl bromides (70) compatible with the reaction. ^a^NEt_3_ (287 equiv.) and MgCl_2_ (250 equiv.) were also used for alkyl bromides.

Lu additionally leveraged a nickel–photoredox dual catalytic system.^108^ Here, an alkyl boronate ester radical precursor 71 was used, a novel precursor for on-DNA photoredox chemistry (Scheme 31). This widens the utility of boronate esters to encompass C(sp^2^)–C(sp^3^) coupling alongside the C(sp^2^)–C(sp^2^) coupling achieved with on-DNA Suzuki couplings. Again, coupling occurs with a DNA-tethered aryl halide, in this case, an aryl bromide (HP-76). A range of benzyl radicals (71a–d) were compatible with the reaction including those bearing a halide (71b), electron-withdrawing group (71c) and electron-donating group (71d).^108^ Extending the conjugation with a naphthalene moiety (71e) saw a slight reduction to the yield. Aliphatic nitrogen heterocycles (71f) gave the desired product HP-77 in good yield, however simple cyclic (71g) and linear (71h) alkyl groups only gave moderate yields.^108^ When the scope of aryl bromide was evaluated with boronate ester 72, those possessing halides (HP-78) and electron donating groups (HP-79) provided excellent yields.^108^ Electron withdrawing groups (HP-80) on the other hand only gave moderate yields. A range of nitrogen heterocycles such as quinoline (HP-81) were also similarly amenable to the reaction conditions.^108^

**Scheme 31:**
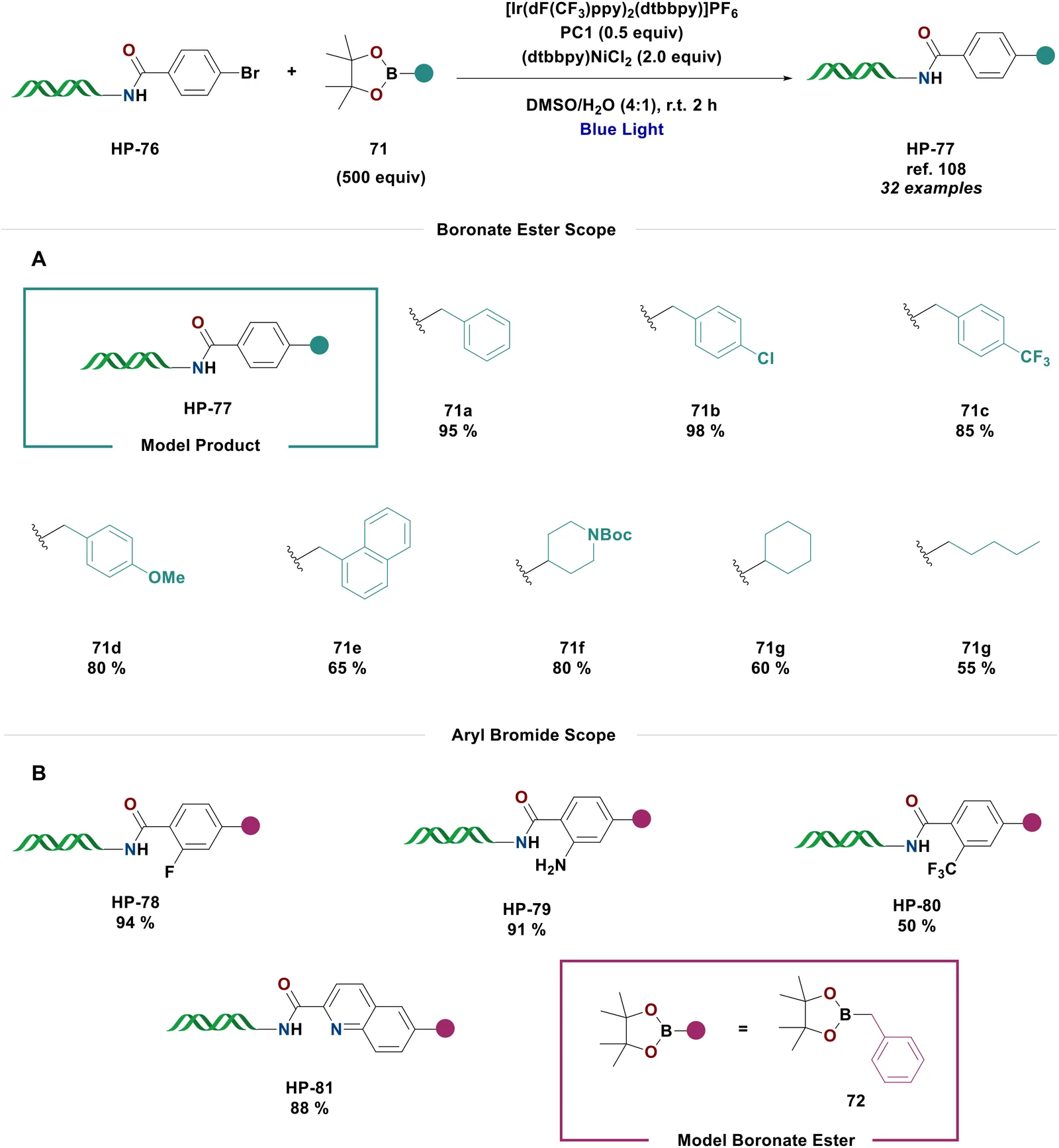
On-DNA reaction between DNA-conjugated aryl bromide HP-76 and boronate ester 71 towards the synthesis of HP-77. (A) Scope of boronate ester compatible with the reaction with HP-76. (B) Scope of DNA conjugated aryl bromides compatible with the reaction with boronate ester 72.

Alongside conventional metallaphotoredox dual catalysis whereby a nickel catalyst is coupled to an iridium photoredox catalyst, transition metals such as palladium can also undergo photoactivation before proceeding onto a classical polar catalytic cycle. Using this principle, Huang recently reported a radical addition of azabicyclo[1.1.0]butanes 73 to 1,3 dienes 74 to give 1-azabicyclo[2.1.1]hexanes 75, a potential pyridine bioisostere.^109^ This reaction was also seen to be amenable to on-DNA synthesis utilising a DNA-conjugated 1,3 diene. In terms of the mechanism, palladium catalyst 76 is initially photoexcited to 77 upon irradiation with blue light (Scheme 32). Simultaneously, azabicyclo[1.1.0]butane 73 reacts with LiBr to form bromide intermediate 78 which can undergo single electron reduction with the excited palladium catalyst 77 to give radical intermediate 79 and palladium(i) species 80.^109^79 can subsequently capture the 1,3-diene 74 to form radical intermediate 81 which can coordinate to the palladium to form a π-allyl intermediate in 82.^109^ The nitrogen-centred anion in 82 can then coordinate to the palladium displacing the bromide before undergoing reductive elimination to afford the product 75 and regenerate the palladium(0) catalyst 76.^109^ This strategy represents a [2π + 2σ] cycloaddition and highlights how using strained precursors such as 73 can allow for σ bonds to become activated towards cycloaddition reactions.

**Scheme 32:**
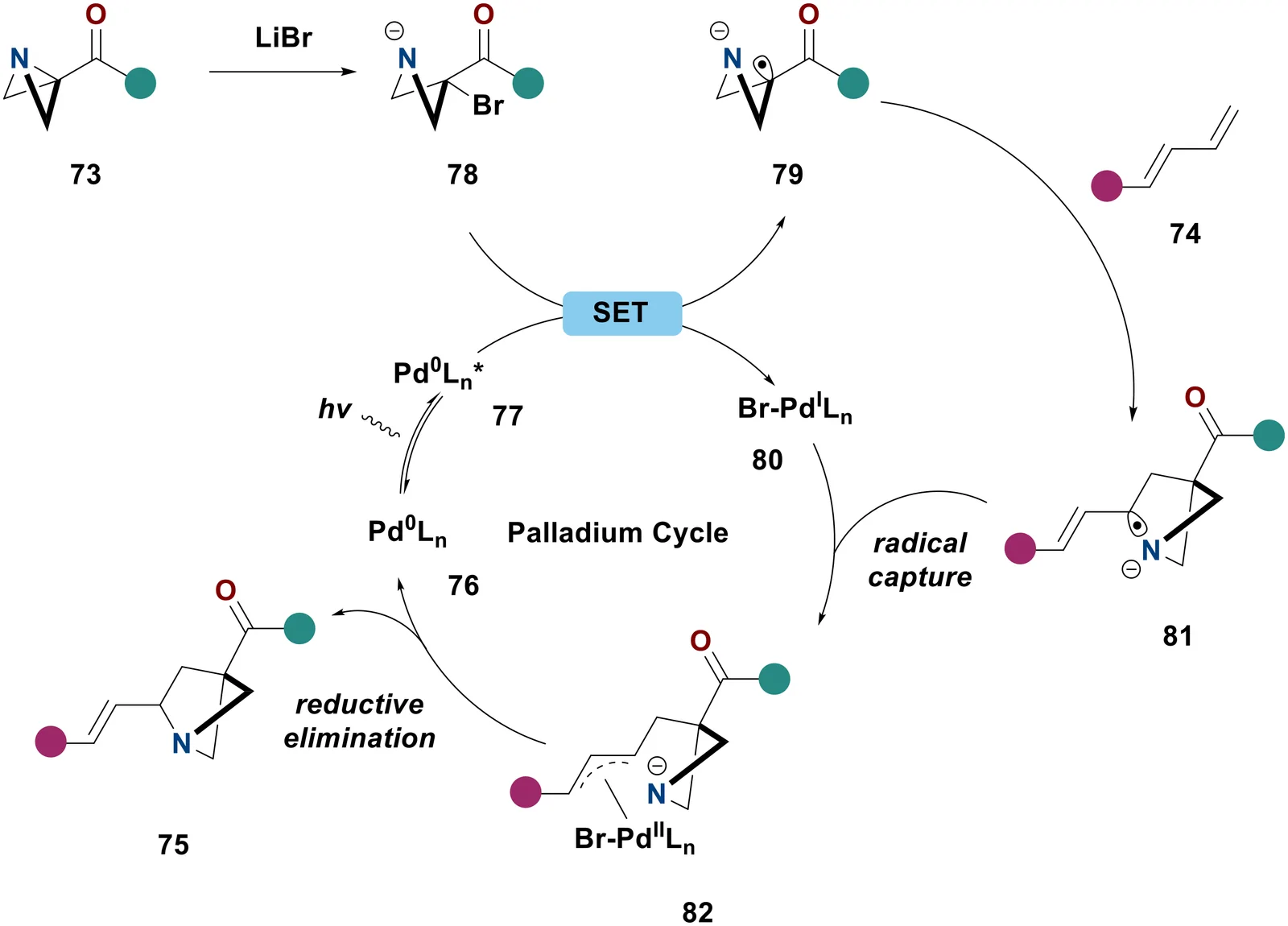
Palladium photoredox catalytic cycle towards the synthesis of 75. Reaction of 73 with LiBr forms bromide intermediate 78. SET from excited Pd catalyst 77 converts 78 into radical intermediate 79 and Pd(i) species 80. Radical 79 captures diene 74 to form 81 – coordination gives 82, which reductively eliminates to afford 75 and regenerate 76.

In the context of on-DNA synthesis, a DNA-conjugated phenyl butadiene moiety HP-82 was chosen as the model substrate. With slight modifications to the reaction conditions, the methodology was seen to tolerate several azabicyclo[1.1.0]butanes including aryl (73a–b), heteroaryl (73c) and alkyl (73d) derivatives (Scheme 33).^109^

**Scheme 33:**
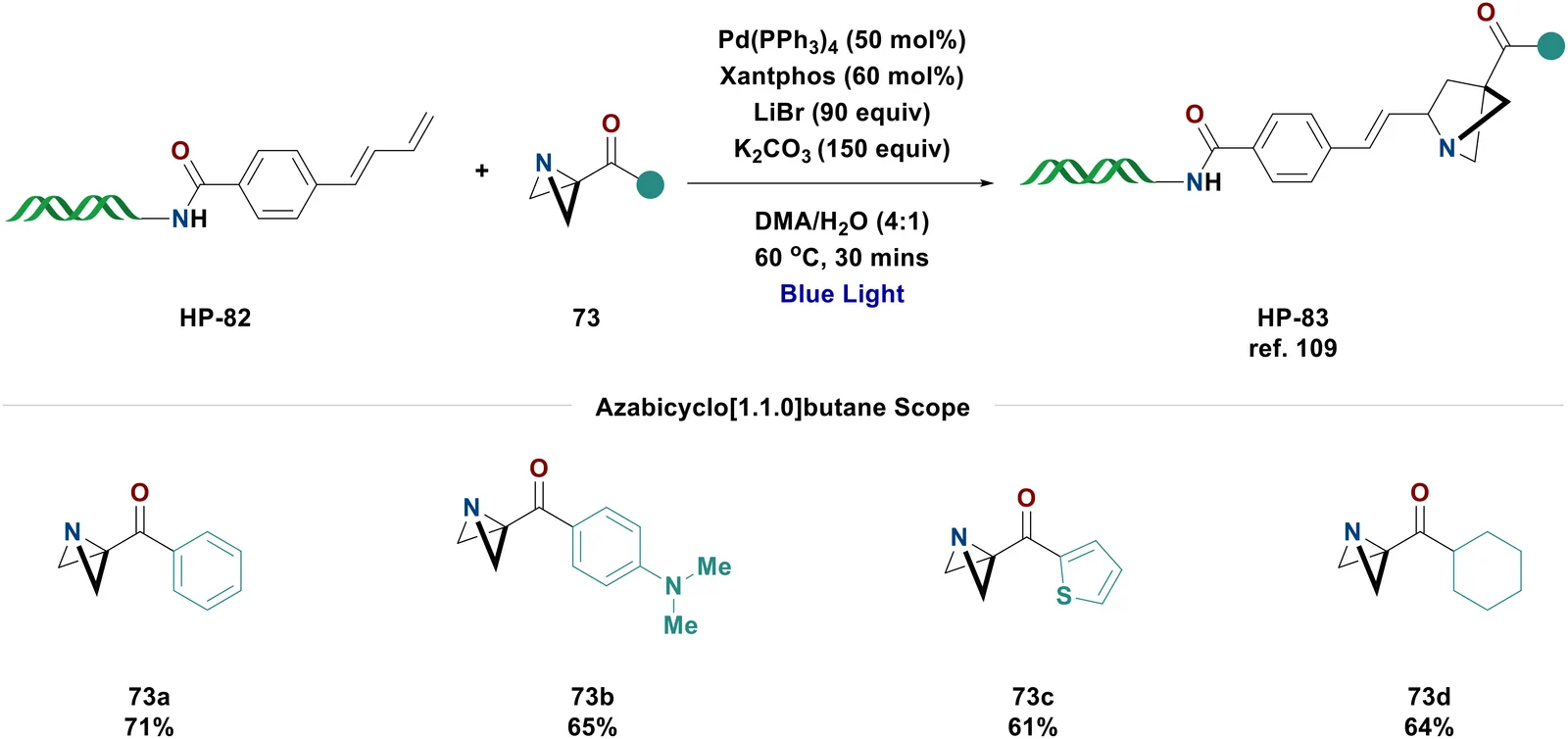
On-DNA [2π + 2σ] cycloaddition reaction between DNA-conjugated 1,3 diene HP-82 and azabicyclo[1.1.0]butane 73 towards the synthesis of HP-83 along with the scope of azabicyclo[1.1.0]butanes compatible with the reaction.

While many on-DNA reactions have been extensively optimised to tolerate water as co-solvent, alternative strategies have been developed to allow for completely inert reactions in pure organic solvents. This strategy typically involves immobilisation or complexation of the negatively charged DNA backbone to a positively charged support which allows the reaction to take place exclusively at the chemical moiety in the presence of an organic solvent before the DNA is released to give the functionalised product. Examples of positively charged solid supports include cationic resins such as PEG+ polymers which are used in the APTAC (amphiphilic polymer-facilitated transformations under anhydrous conditions) approach,^110^ and polystyrene strong anion exchange resins, such as Strata-XA or PAX, which are used in the reversible adsorption to solid support (RASS) strategy.^111^ In addition, didecyldimethylammonium bromide (DDAB) surfactants can be used to translate this principle into more traditional solution phase chemistry and carry out surf–DNA reactions.^112,113^ Here, the cationic surfactant forms a temporary complex with the DNA backbone allowing for the photochemical reaction to be carried out under inert conditions in organic solvent before a saturated salt solution is added to remove the surfactant during the work-up.

In the context of metallaphotoredox catalysis, the APTAC and surf–DNA strategies have been utilised to carry out the classic sp^2^–sp^3^ coupling between aryl halides and alkyl radicals in a photoredox–nickel dual catalytic system (Scheme 34).^110,112,113^ In the APTAC approach, carboxylic acids 83 were used as a radical precursor to generate alkyl radicals that could couple to the PEG+ supported aryl halide HP-84 and afford functionalised products HP-85 following a saturated NaCl wash.^110^ In the case of the surf–DNA strategy carried out by Chheda, the addition of the DDAB surfactant, allowed the headpiece HP-84 to fully dissolve in DMSO.^112,113^ Radical precursors such as carboxylic acid 84 or boronate ester 85 could then be used to couple to the aryl halide and afford product HP-86 or HP-87. Despite these promising results, surf–DNA remains an emerging platform. The surfactant-mediated transfer of DNA conjugates into organic solvent enables efficient coupling chemistry, but recovery of the DNA after reaction remains a key limitation, with only 65% recovery reported.^112^ This recovery penalty places additional pressure on reaction efficiency and fidelity, since incomplete conversion or significant by-product formation would further erode the useful library population. Nevertheless, the approach is conceptually powerful. For example, with further optimisation of surfactant structure, work-up and DNA-release conditions, these could provide access to water-sensitive transformations that are currently inaccessible under conventional aqueous on-DNA conditions.

**Scheme 34:**
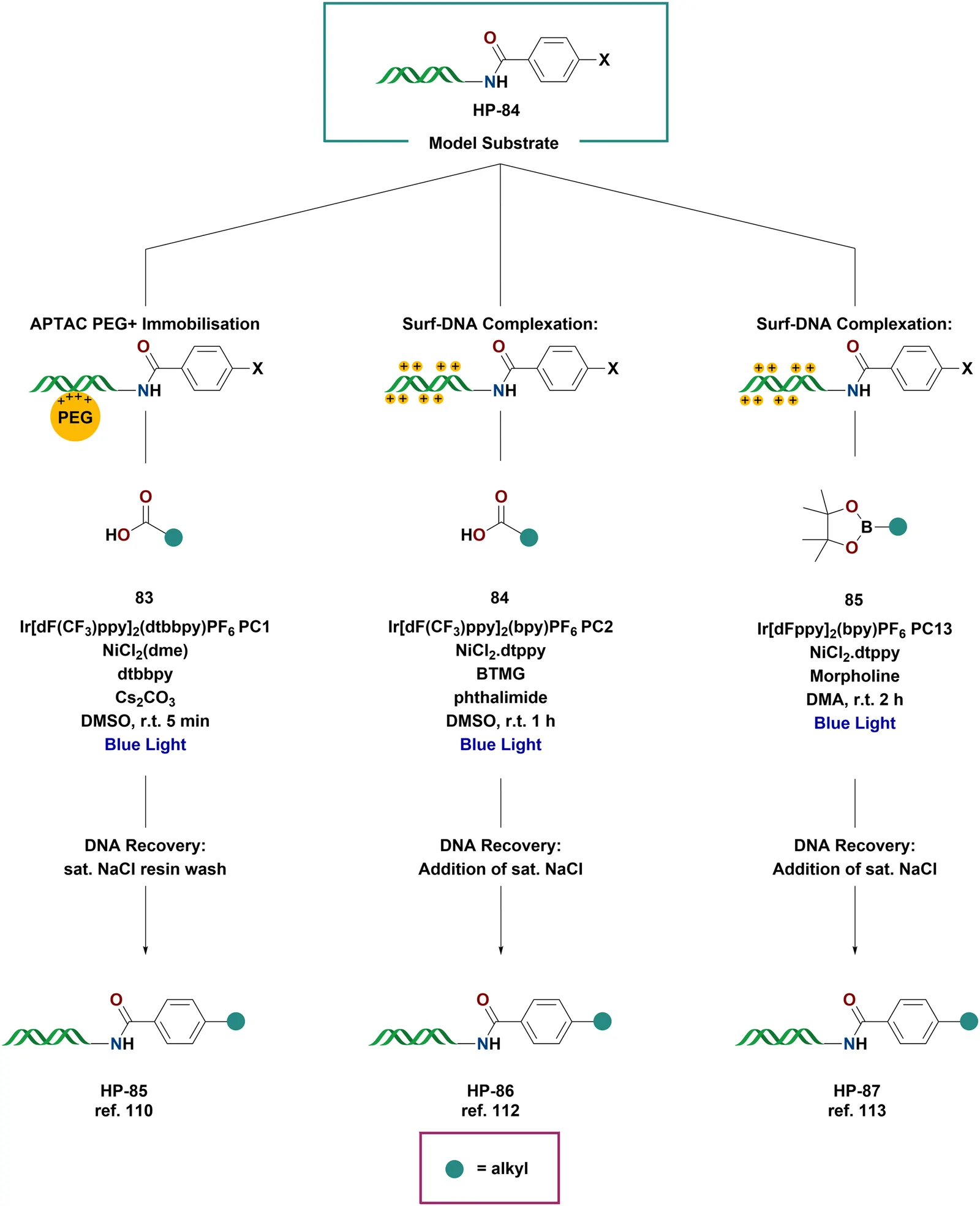
On-DNA aryl–alkyl coupling reactions on DNA-conjugated aryl halides HP-84 using carboxylic acid 83–84 or boronate ester 85 radical precursors. Reactions were carried out under inert conditions, in neat organic solvent, without the need for water as a co-solvent. To facilitate this, the DNA barcode was coordinated to a PEG+ bead for the APTAC approach or it was coordinated to a DDAB surfactant for the surf–DNA approach.

### 3.4 Electron donor–acceptor (EDA) complex

Electron donor–acceptor (EDA) complexes provide a complementary strategy for radical generation without the need for an exogenous photocatalyst.^114^ These EDA complexes are formed in the ground state and are diffusion-limited.^25,114^ While neither the electron donor (ED) or electron acceptor (EA) species are capable of absorbing light on their own, upon the formation of the EDA complex this becomes possible and allows for a SET event to form two radical species (Scheme 35).^25,90,114^ In the case of photoredox chemistry, a highly conjugated nitrogen heterocycle such as a Hantzsch ester or NADP derivative is typically employed as the ED while a redox-active ester (RAE) such as *N*-hydroxyphthalimide is utilised as an effective EA. Following SET and radical formation the RAE can undergo decarboxylation to release a radical intermediate that can react with a coupling partner. The second radical intermediate formed from this coupling can then abstract a hydrogen from another equivalent of the ED to form the desired functionalised product.^90^

**Scheme 35:**
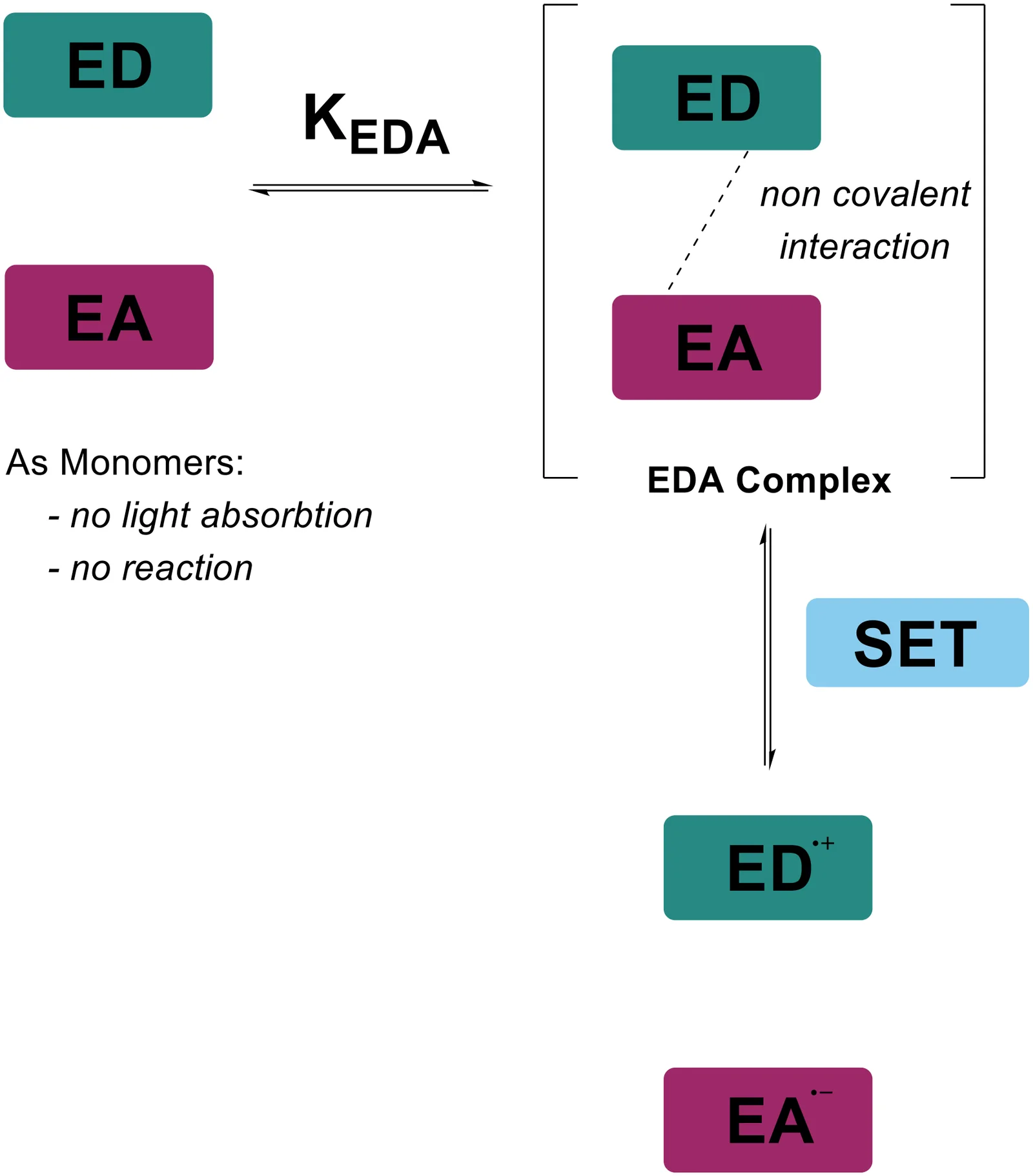
Radical formation from an electron donor–acceptor (EDA) complex. Two chemical species (ED and EA) are unable to absorb light or react as monomers. However, through non-covalent association, they can form an EDA complex with a new charge-transfer absorption band. Upon irradiation, this complex undergoes single-electron transfer from the electron donor to the electron acceptor, generating a pair of radical species.

Mendoza reported the use of NADH and BuNAH (an analogue of NADH) as an effective ED in on-DNA Giese reactions involving Michael acceptor HP-88 affording product HP-89 (Scheme 36).^115^*N*-Hydroxyphthalimide 86 was used as the EA and radical precursor molecule. Most of this report focuses on developing the methodology off-DNA therefore the scope of on-DNA products highlighted is relatively limited. Despite this, good to excellent reactivity was seen throughout. Both secondary (86a–b) and tertiary (86c–d) radicals were well tolerated including secondary α-amino radical 86b and tertiary radical 86d derived from the drug gemfibrozil.^115^ This report provided the proof-of-concept that EDA complexes were effective at enabling photoredox coupling on-DNA in high yield.

**Scheme 36:**
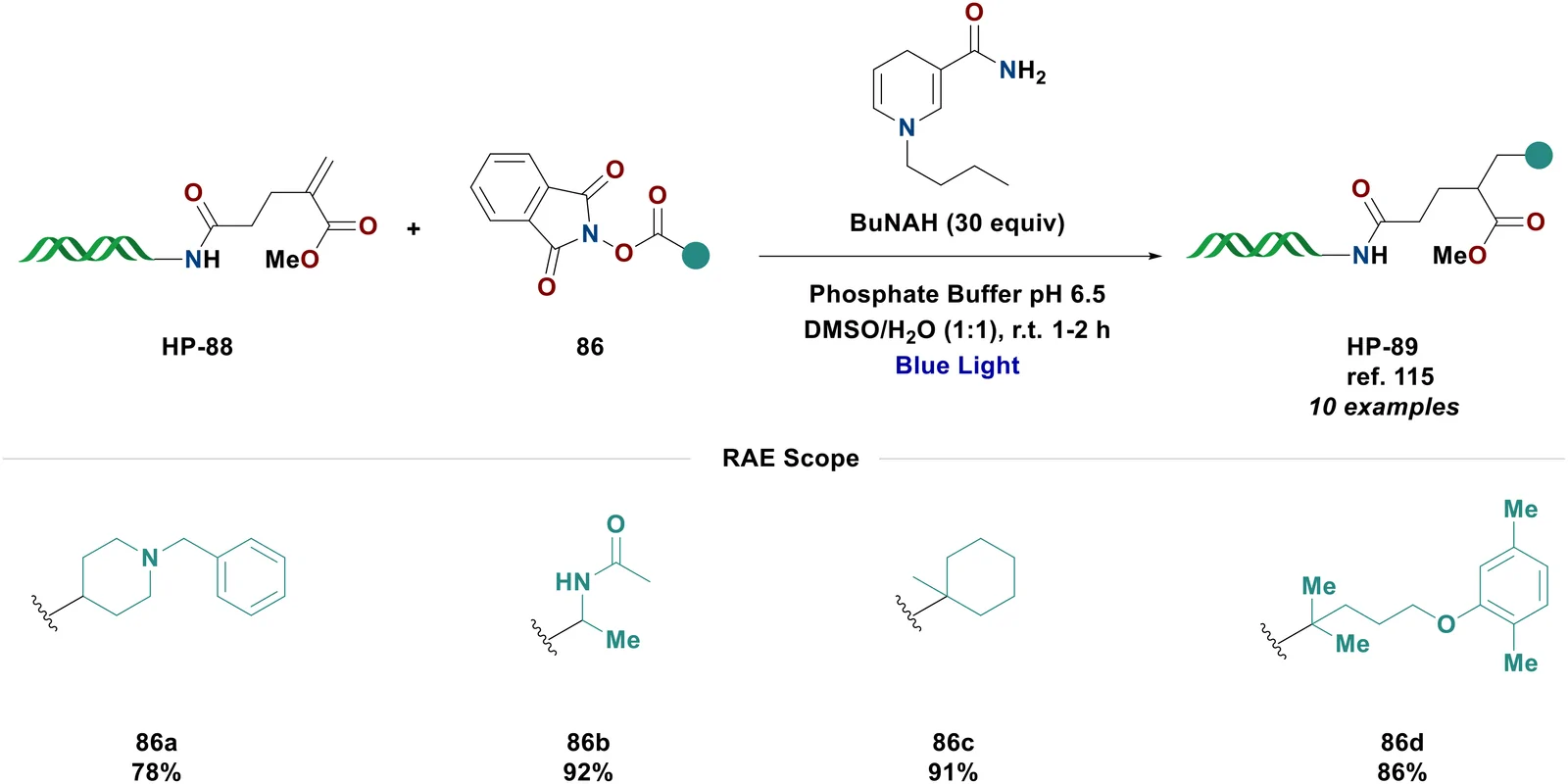
On-DNA Giese reaction between DNA-conjugated alkene HP-88 and redox-active ester (RAE) 86 towards the synthesis of HP-89 along with the scope of RAEs compatible with the reaction. EDA complex formed between the RAE and BuNAH.

Molander subsequently extended this concept to an EDA-enabled Giese reaction. Here, a Hantzsch ester was used as the ED and a *N*-hydroxyphthalimide 87 was again used as the EA and radical precursor.^90^ Following SET and decarboxylation, radical addition to DNA-conjugated trifluoromethyl-substituted alkene HP-90 occurs to form products of type HP-91 (Scheme 37). Unlike in previous reactions using trifluoromethyl-substituted alkenes, an E1cb type elimination of a fluoride does not occur and trifluoromethylated product HP-91 is formed rather than *gem*-difluoroalkenes. This is due to the fact that this process is terminated by a HAT from the Hantzsch ester rather than reduction by a PC to form an anion species that can then undergo the E1cb.^90^ A broad range of redox-active ester-derived radicals were compatible with the reaction conditions. Primary radicals such as benzyl radical (87a) and amino radical (87b) gave products in good to excellent yield.^90^ Secondary radicals (87c) were exceptionally well tolerated including strained ring systems (87d) and heterocycles (87e).^90^ Moderate to good yields were observed with tertiary radicals (87f–h).^90^ Importantly 87g was the first example of BCP being incorporated into on-DNA chemistry and paved the way for further exploration of this motif in the iridium-PC-catalysed Giese reaction with BCP halides as described previously.^61^ The reactivity of tertiary radical 87h derived from gemfibrozil was seen to be similar to that reported by Mendoza (86d).^115^ This gemfibrozil RAE precursor was subsequently used to investigate the reaction's tolerance to variation in the DNA-tethered activated alkene. Using this gemfibrozil RAE precursor (87h), good to excellent conversion was maintained upon the addition of a chloride (HP-92) or a methoxy group (HP-93) to the phenyl ring of the vinyl arene.^90^ However, incorporation of a methyl substituent reduced the yield to 47%, consistent with increased steric congestion around the alkene (HP-94). Beyond trifluoromethyl-substituted styrenes, a range of DNA-conjugated vinyl heterocycles such as vinyl pyrazole HP-95 and vinyl triazole HP-96 were seen to tolerate the reaction conditions well.^116^ Overall, this methodology gave rise to a versatile scope and provides a new mode of reactivity for DNA-conjugated trifluoromethyl-substituted alkenes, making these substrates prime candidates for inclusion into DELs.

**Scheme 37:**
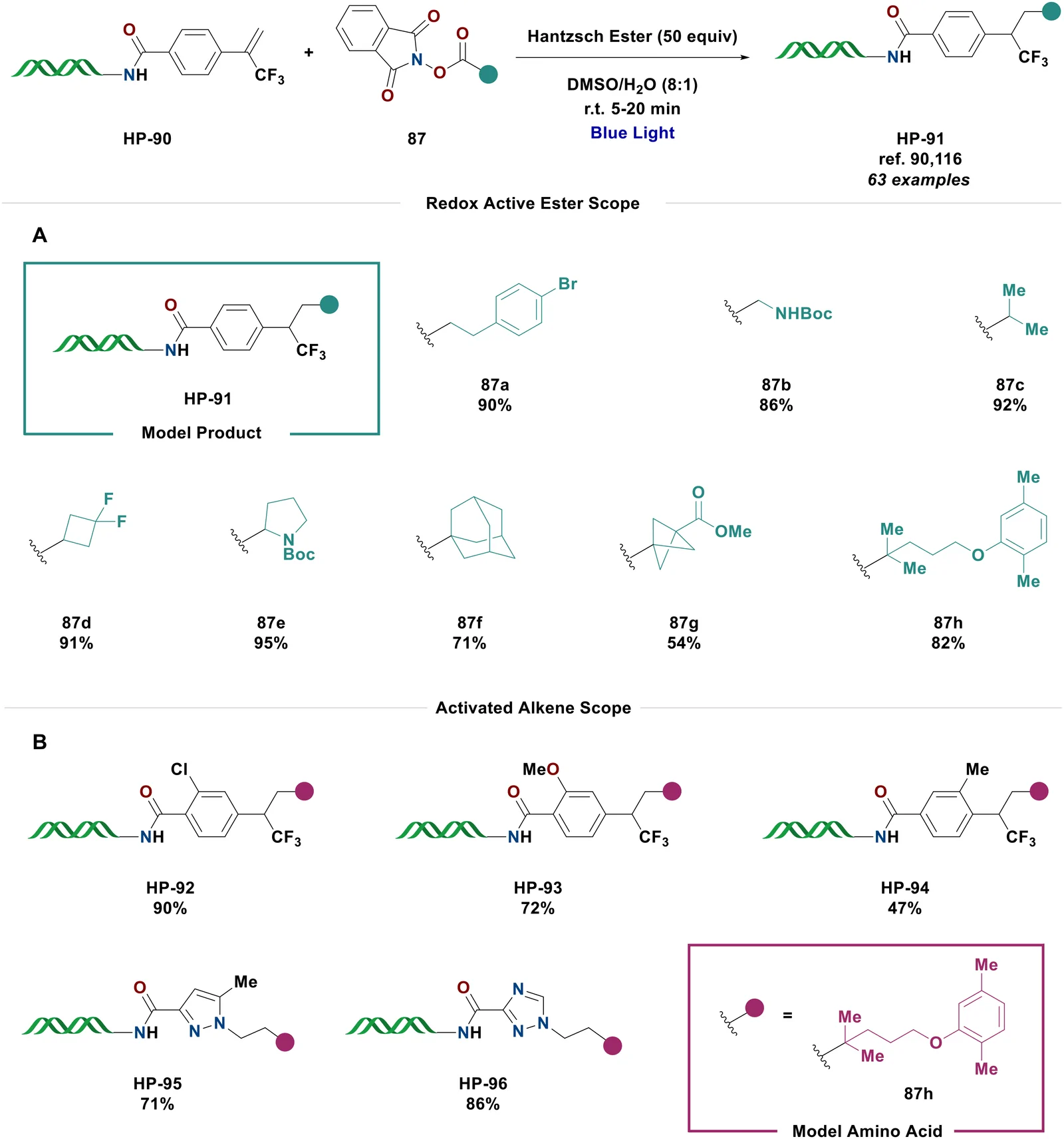
On-DNA Giese reaction between DNA-conjugated alkene HP-90 and redox-active ester (RAE) 87 towards the synthesis of HP-91. (A) Scope of RAEs compatible with the reaction with HP-90. (B) Scope of DNA conjugated alkenes tolerated with RAE 87h. EDA complex formed between the RAE and Hantzsch ester (HE).

Along with Giese-type reactions, another class of reactions that has been adapted to on-DNA synthesis by utilising an EDA complex is the C(sp^2^)–C(sp^3^) coupling of alkyl radicals derived from feedstock alcohols to a DNA-conjugated pyridine as reported by Nappi (Scheme 38).^117^ In this methodology, two separate EDA complexes form: one between the Hantzsch ester and the DNA-tethered cyano-pyridine (HP-97) and another between another equivalent of Hantzsch ester and a 4-cyano-2-fluorobenzoate ester 88 which can be easily synthesised from feedstock alcohols.^117^ This generates a persistent cyano-pyridine radical anion and an alkyl radical which forms following β-scission of the benzoate radical anion.^117^ These two radical species can then undergo radical–radical coupling followed by elimination of the cyano group to facilitate re-aromatisation and give the desired product HP-98. The on-DNA examples demonstrate that primary, secondary and tertiary radicals can all be incorporated effectively when stabilised by an adjacent aryl group (88a–c). Other aromatic systems also proved competent, with indole derivative 88d giving a moderate yield.^117^ The reaction was also compatible with more complex substrates, including the haloperidol-derived product 88e.

**Scheme 38:**
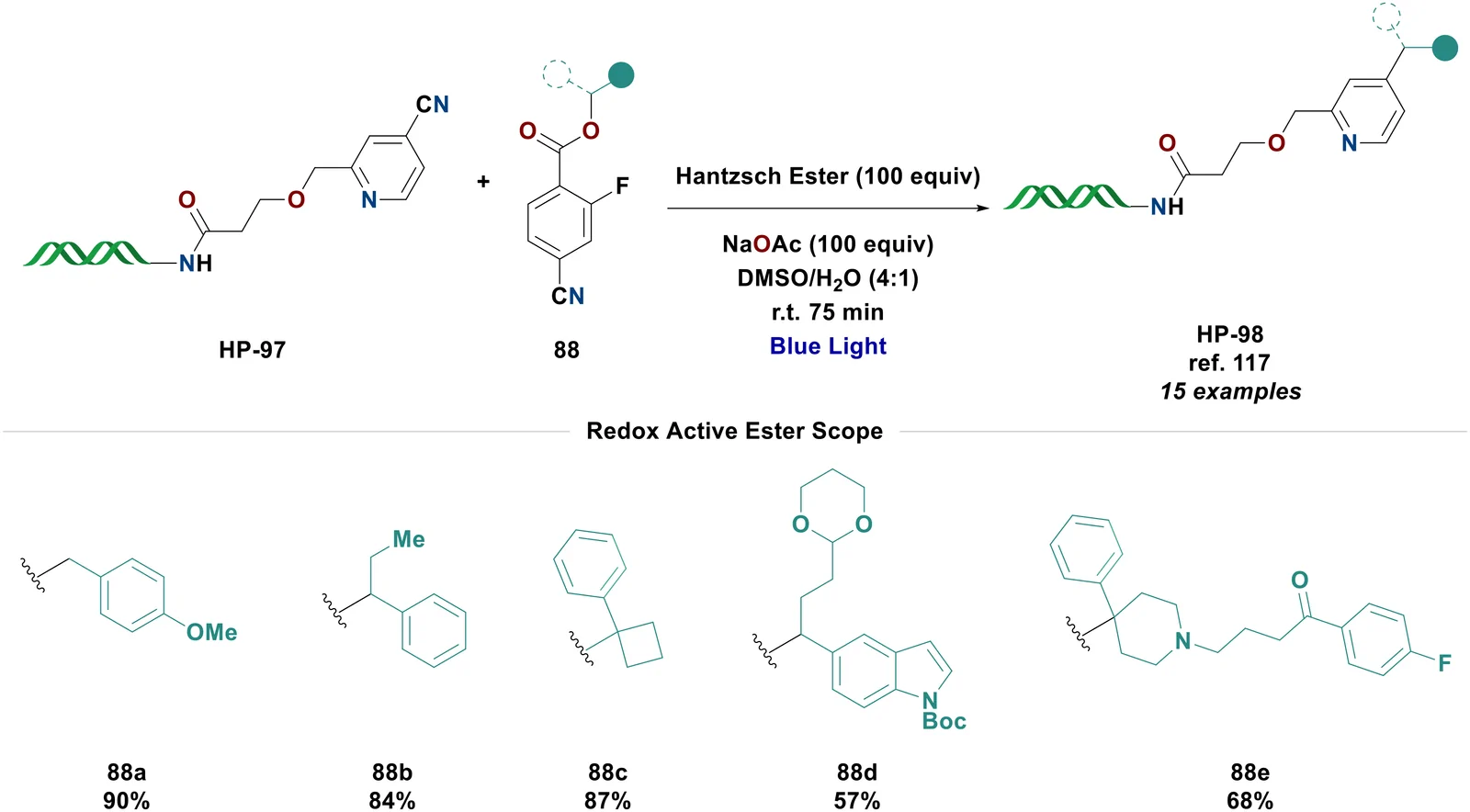
On-DNA substitution reaction between DNA-conjugated cyano pyridine HP-97 and redox-active ester (RAE) 88 towards the synthesis of HP-98 along with the scope of RAEs compatible with the reaction. EDA complex formed between the RAE and Hantzsch ester (HE).

As well as forming C–C bonds, EDA complexes are commonly used in C–S bond formation reactions. Recent work by the Zhang research group has utilised aryl sulfinates 89 which can form effective EDA complexes with DNA-conjugated aryl halides HP-99 or thianthrenium (TT) salts HP-100 and generate diaryl sulfones HP-101 or HP-102 (Scheme 39).^118,119^ With DNA conjugated iodoindole HP-99, aryl sulfinates bearing alkyl 89a, electron-donating 89b and electron-withdrawing 89c substituents were reported to give excellent yields of the sulfone product HP-101 as were heteroaryl sulfinates (89d).^118^ Electron-withdrawing sulfinate 89c was particularly effective in giving near quantitative yield. Alkyl sulfinates (89e–f) were also seen to tolerate the reaction conditions, albeit with more moderate yields.^118^ This is likely due to the fact that alkyl sulfinates do not form EDA complexes as readily. With DNA conjugated TT salts, moderate to good yields were observed with a range of aryl sulfinates. Again, alkyl 89g, electron-donating 89h and electron-withdrawing 89i substituents were tolerated along with heteroaryl sulfinates (89j–k).^119^ As with the aryl halides, electron-withdrawing substituents on the sulfinate enhanced formation of the diaryl sulfone (HP-102). While alkyl sulfinates were not reactive with the aryl TT salt, sulfinates with more highly conjugated aryl groups such as 89k were seen to be amenable here and thus expand the scope over what was possible with the aryl halides. Furthermore, while the yields with the TT salts were seen to be diminished overall, their ease of synthesis from a mild C–H activation with feedstock arenes makes them excellent substrates for this chemistry.^119^ For both of these reactions, a RASS approach was used and allowed the reaction to be carried out in DMSO without using water as a co-solvent. For this approach, the DNA was immobilised on a cationic PAX resin while the photoredox reaction was carried out before being eluted upon the addition of a saturated salt solution after the reaction was complete.^111,119^ It is also worth noting here, that the use of a RASS system allows for the use of sulfinate 89 and caesium carbonate in vast excess and several orders of magnitude higher than typical on-DNA reactions and this likely contributes to the high yields seen here, especially in the case of iodoindole substrate HP-99.

**Scheme 39:**
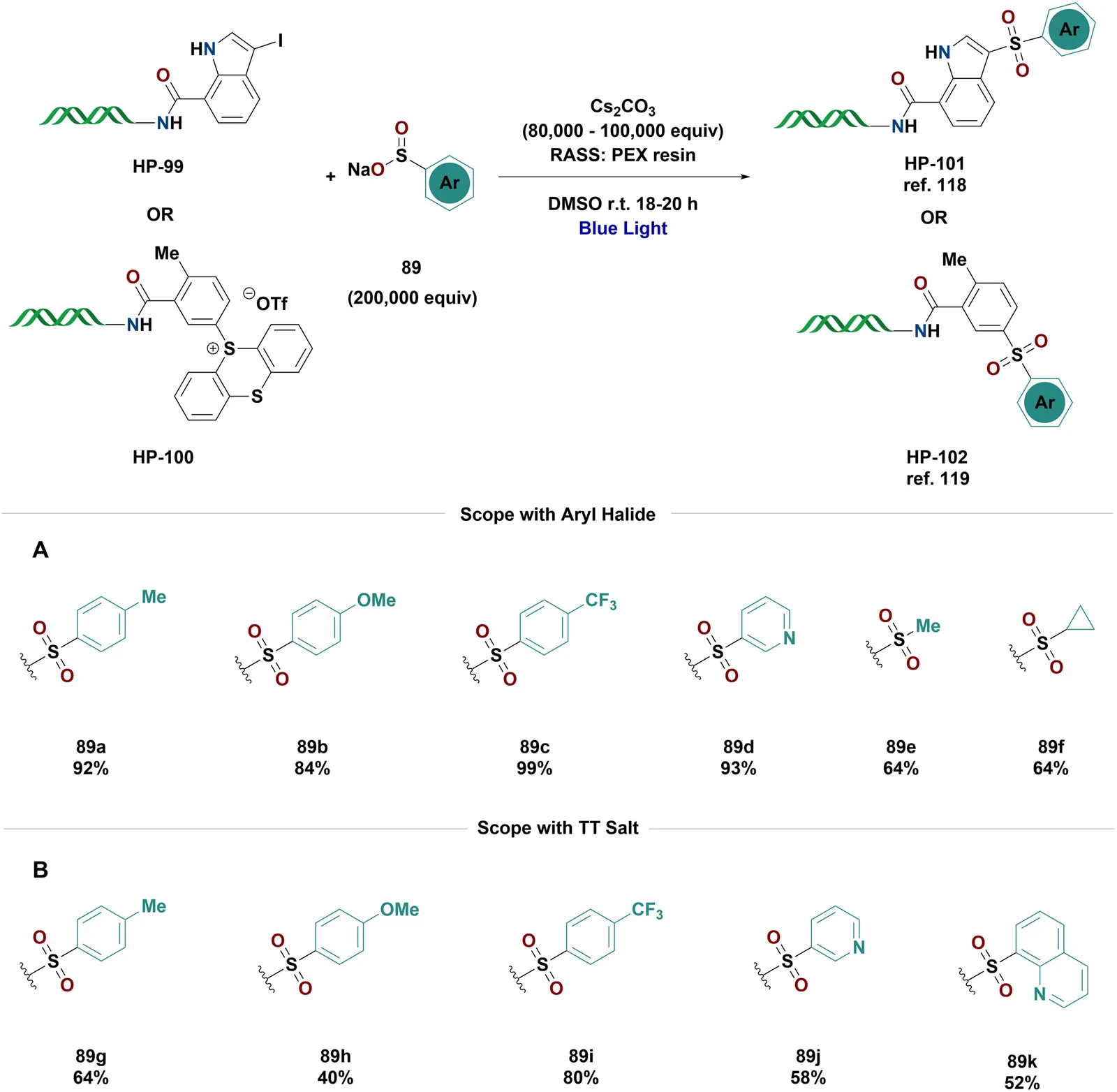
On-DNA reaction between DNA-conjugated aryl halides HP-99 or thianthrenium (TT) salts HP-100 and aryl sulfinates 89 towards the synthesis of HP-101 and HP-102. (A) Scope of sulfinates 89 tolerated by the reaction with DNA conjugated aryl iodides HP-99. (B) Scope of sulfinates 89 tolerated by the reaction with DNA conjugated TT salts HP-100. EDA complex formed between the sulfinate 89 and DNA conjugated aryl iodide HP-99 or TT salt HP-100. Reactions were carried out under inert conditions using RASS.

Along with diaryl sulfones, diaryl sulfides have also been constructed on-DNA under photoredox conditions using an EDA complex. This has been reported by the Hu research group whereby DNA-conjugated phenylthiols HP-103 were coupled with aryl halides 90 (Scheme 40).^120^ The reaction was seen to tolerate phenyl iodide 90a along with derivatives bearing electron-donating and withdrawing substituents (90b–c).^120^ Sterically bulky (90d) and heterocyclic (90e) aryl iodides were also seen to perform well.^120^ Beyond aryl iodides, alkyl iodides were also seen to tolerate the reaction. Here, the EDA complex is slightly different. Rather than a complex facilitated by π-stacking as seen in the previous examples, here the thiolate anion coordinates to the iodine and upon irradiation donates an electron to the alkyl iodide to form an alkyl radical anion species and a thiolate radical.^120,121^ The alkyl radical anion can then release iodide to form an alkyl radical. This alkyl radical can then either couple to another thiolate anion and form another radical anion that propagates the cycle or it can undergo a radical combination with the thiolate radical to terminate the cycle and form sulfide HP-104.^120,121^ A broad scope of alkyl iodides were seen to be amenable including primary (90f), secondary (90g) and tertiary (90h) examples including structurally rich bioisosteres such as BCP 90i and cubane 90j.^120^

**Scheme 40:**
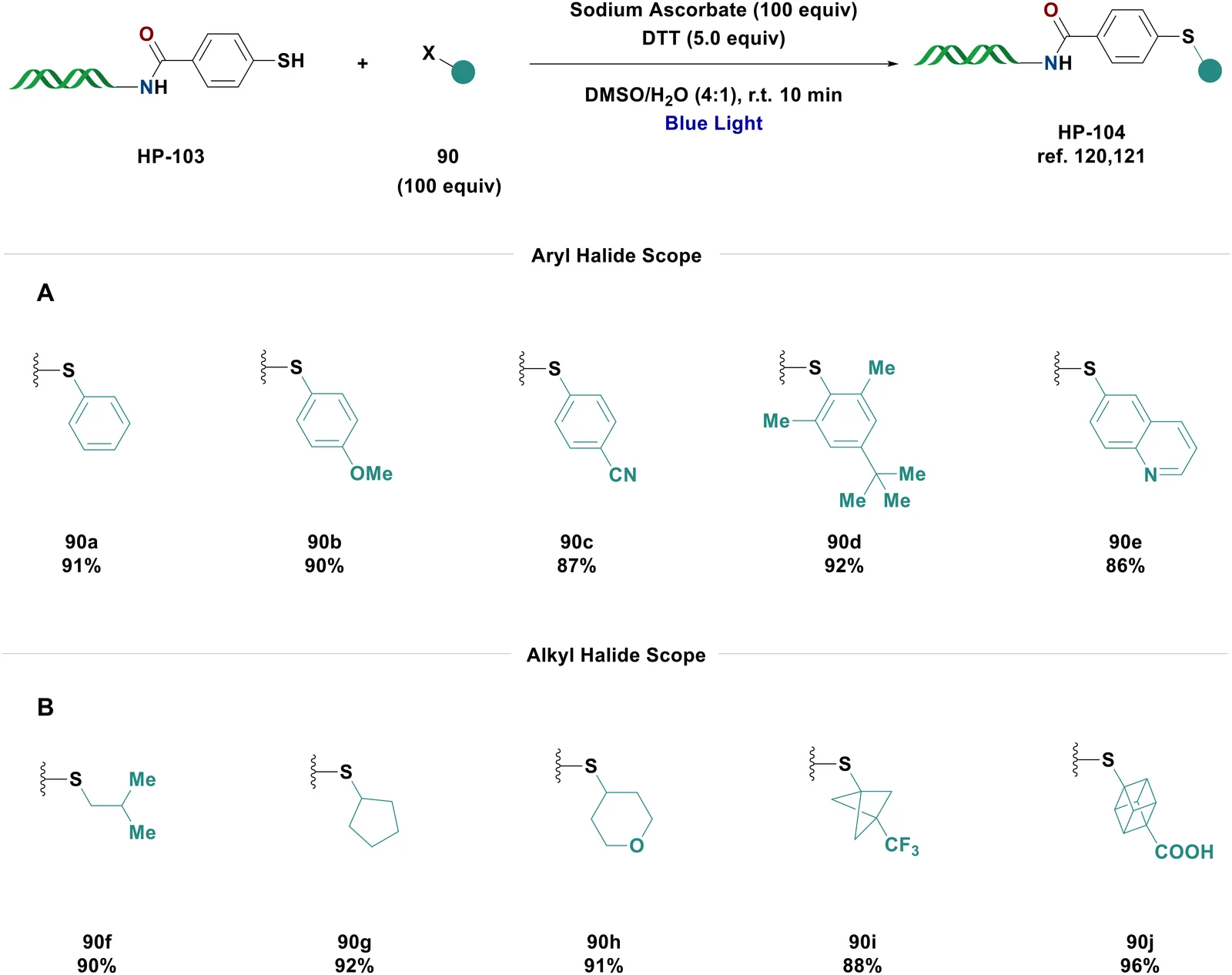
On-DNA reaction between DNA-conjugated thiophenol HP-103 and aryl or alkyl iodides 90 towards the synthesis of HP-104. (A) Scope of aryl iodides compatible with the reaction with DNA conjugated thiophenol HP-103. (B) Scope of alkyl iodides compatible with the reaction with DNA conjugated thiophenol HP-103. EDA complex formed between the DNA conjugated thiophenol HP-103 and iodide 90.

### 3.5 Energy transfer (EnT) catalysis

Beyond photoredox pathways, triplet energy-transfer (EnT) catalysis provides a complementary activation mode in which the photocatalyst acts as a photosensitiser rather than as a redox reagent. Upon absorption of light, the ground-state photocatalyst is promoted from S_0_ to an excited singlet state, S_1_.^56,122–124^ For many transition-metal and organic photocatalysts, this singlet excited state undergoes rapid intersystem crossing to generate a longer-lived triplet excited state, T_1_. This triplet-state photocatalyst can then transfer energy to a suitable ground-state substrate through triplet–triplet energy transfer, typically by a Dexter-type exchange mechanism.^56,122–124^ This short-range process requires close catalyst–substrate contact and orbital overlap, and formally involves a concerted exchange of two electrons. The photocatalyst is thereby returned to its ground state while the substrate is promoted to its triplet excited state. Efficient EnT requires that the photocatalyst has a triplet energy equal to, or greater than, that of the target substrate, together with a sufficiently long triplet lifetime to allow productive substrate encounter.^56,122^ In EnT-mediated alkene [2 + 2] cycloadditions, triplet sensitisation of 91 generates an excited alkene 92 with diradical character, which can add to a second alkene 93 to form a triplet 1,4-diradical intermediate 94 (Scheme 41). Subsequent intersystem crossing to the singlet surface (95) enables radical recombination and formation of the cyclobutane product 96.

**Scheme 41:**
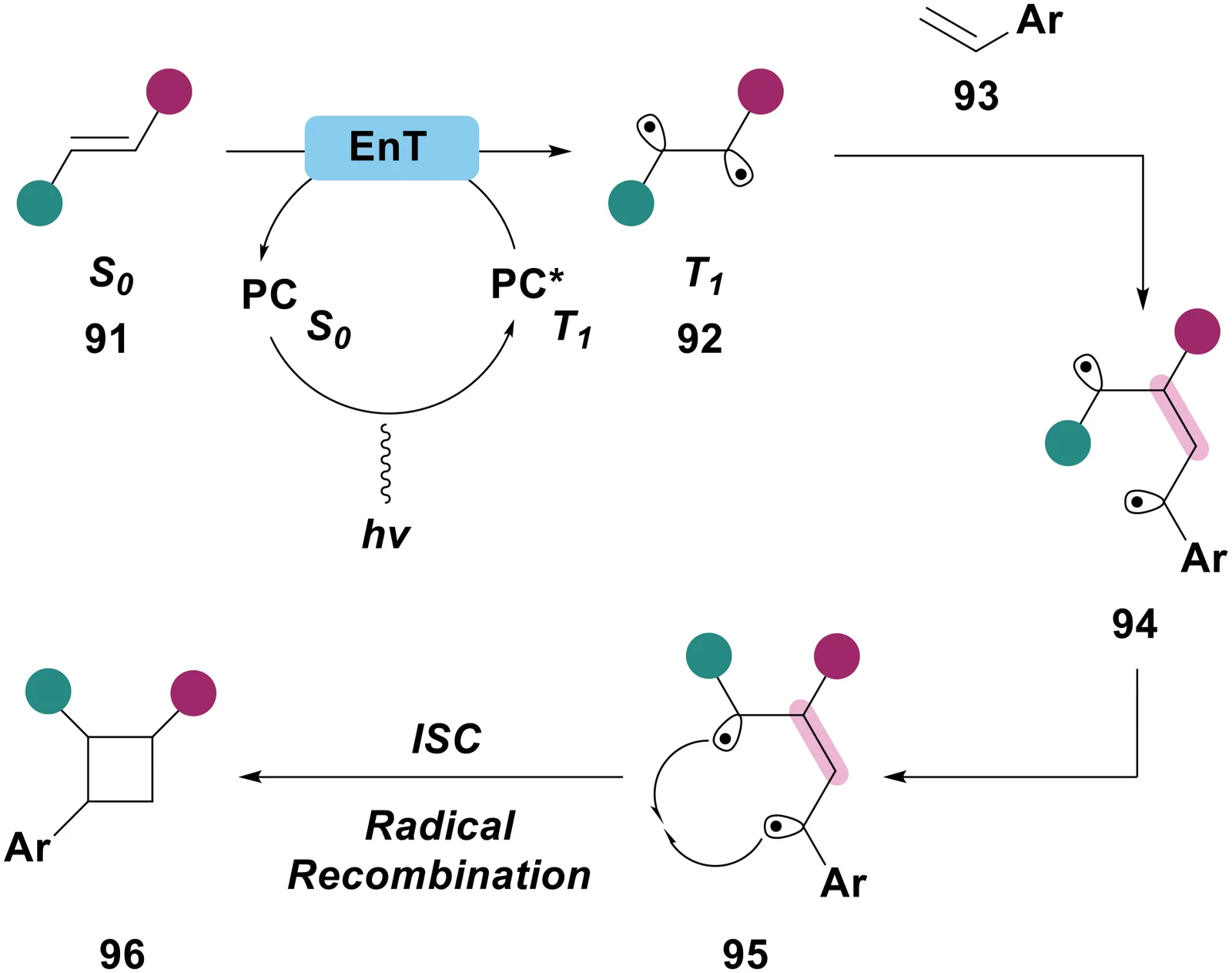
Mechanism for an energy-transfer (EnT) catalysed photochemical [2 + 2] reaction. Energy transfer from an excited PC* to alkene 91 generates diradical species 92. 92 can capture an aryl alkene 93 to form a new C–C bond in 94 which can undergo ring closure to form cyclobutane product 96.

The first on-DNA example of this EnT strategy was reported by Pfizer in 2020. They showed that 1,2-disubstituted alkenes, including cinnamates and enones, underwent on-DNA [2 + 2] cycloaddition with DNA-tethered styrenes (HP-105) to give cyclobutane HP-106 in moderate to good yield (Scheme 42).^77^ Variation in the aryl group of the cinnamate coupling partner (97) was evaluated and seen to tolerate the inclusion of halides (97b), and electron-donating groups (97c).^77^ However strong electron-withdrawing groups were not established in the scope, suggesting that these groups were not tolerated. This trend is consistent with the requirement for a sufficiently reactive triplet alkene, as strongly electron-withdrawing substituents may alter the alkene triplet energy and reduce productive diradical formation. The replacement of the phenyl ring with nitrogen heterocycles such as indole (97d) and quinoline (97e) also resulted in moderate to good yields.^77^ Beyond cinnamates, other enone derivatives were tolerated including carboxylic acid derivative 97f, aldehyde derivative 97g and ketone derivative 97h.^77^ This report is useful because in many cases, the diastereoisomer peaks were separable by LCMS allowing for diastereomeric ratios to be calculated.

**Scheme 42:**
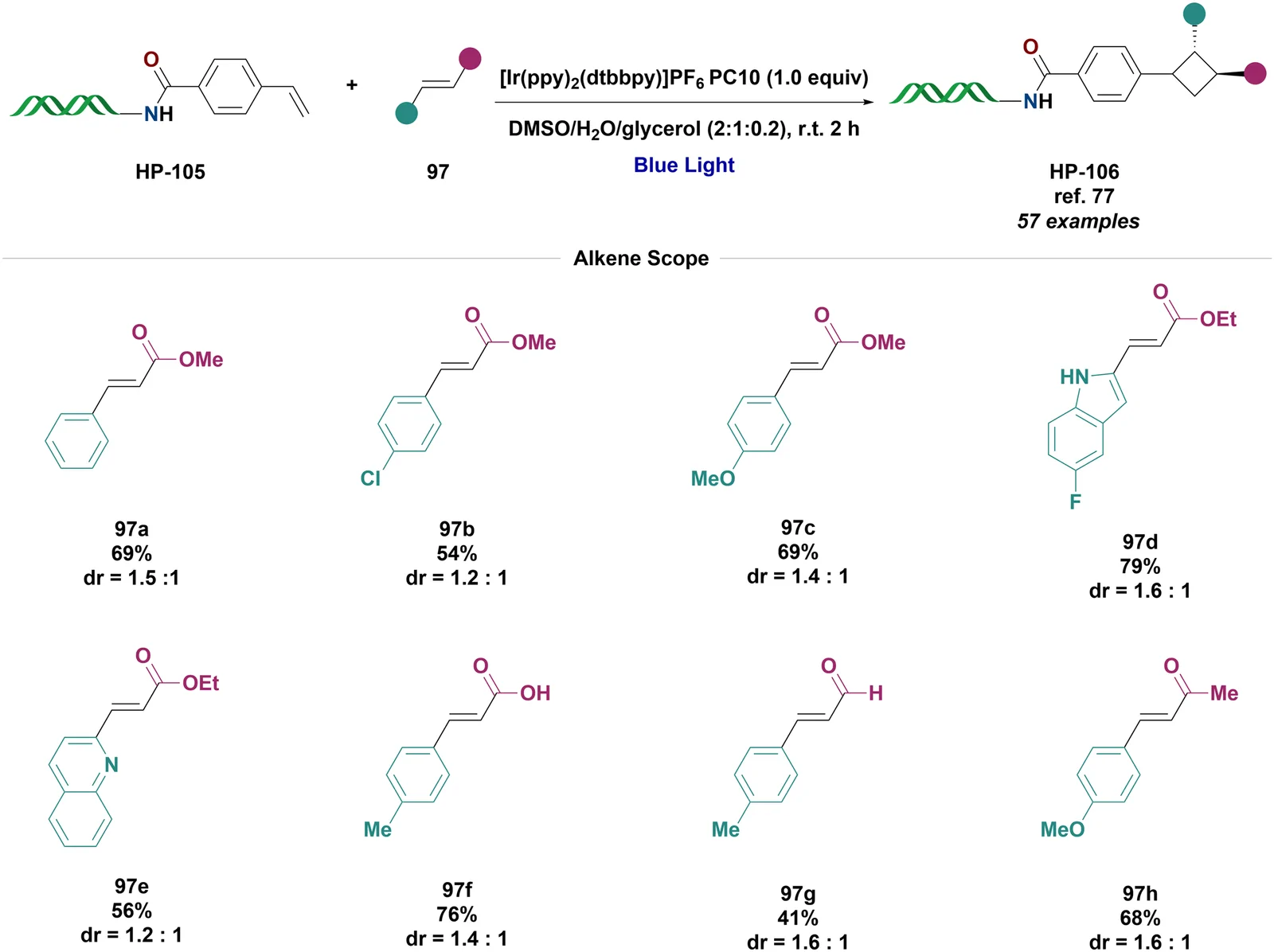
On-DNA [2 + 2] cycloaddition reaction between DNA-conjugated styrene HP-105 and disubstituted alkenes 97 towards the synthesis of HP-106 along with the scope of alkenes compatible with the reaction.

Following this proof-of-concept, the Molander group sought to develop an on-DNA [2 + 2] reaction of their own. For this, they took inspiration from their previous work developing an on-DNA Giese reaction involving BCP iodides whereby it was revealed that employing a DNA-conjugated BCP iodide HP-107 resulted in a hydrolytic ring opening to afford an *exo*-methylenecyclobutanol HP-108 (Scheme 43).^61,125^ This provided the perfect substrate and platform to develop an on-DNA [2 + 2] reaction.

**Scheme 43:**
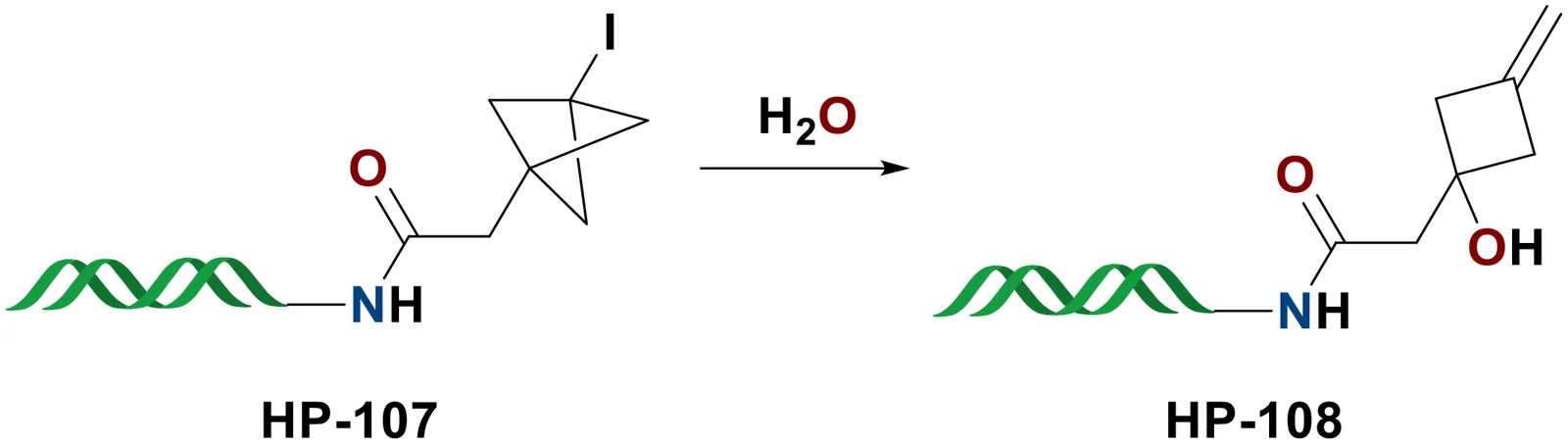
On-DNA hydrolytic ring opening of BCP iodide HP-107 to *exo*-methylenecyclobutanol HP-108.

Indole derivatives 98 (a privileged medicinal chemistry fragment) were selected as the other coupling partner resulting in the formation of complex spiro[3.3]heptane structures HP-109 (Scheme 44).^125^ This process was demonstrated to have a broad scope with many excellent yields above 90%. Using DNA substrate HP-108, the scope of indoles was explored and the reaction was seen to proceed in the presence of a halide (98b–c) and electron-withdrawing substituent (98d).^125^ Electron-donating substituents proved to make the indole less reactive with an azaindole being required to achieve a good 77% yield (98e).^125^ Alternatively, replacing the ester group in the 2-position with a double Boc protected amide (98f) was well tolerated however, the inclusion of a cyano group in this position (98g) was less favourable.^125^ Furthermore, a range of DNA-tethered alkenes was also explored with indole 99. This included *exo*-methylenecyclobutanes (HP-110), even those already bearing spirocyclic structures (HP-111 and HP-112), along with an unactivated alkene (HP-113) which was seen to be less reactive.^125^

**Scheme 44:**
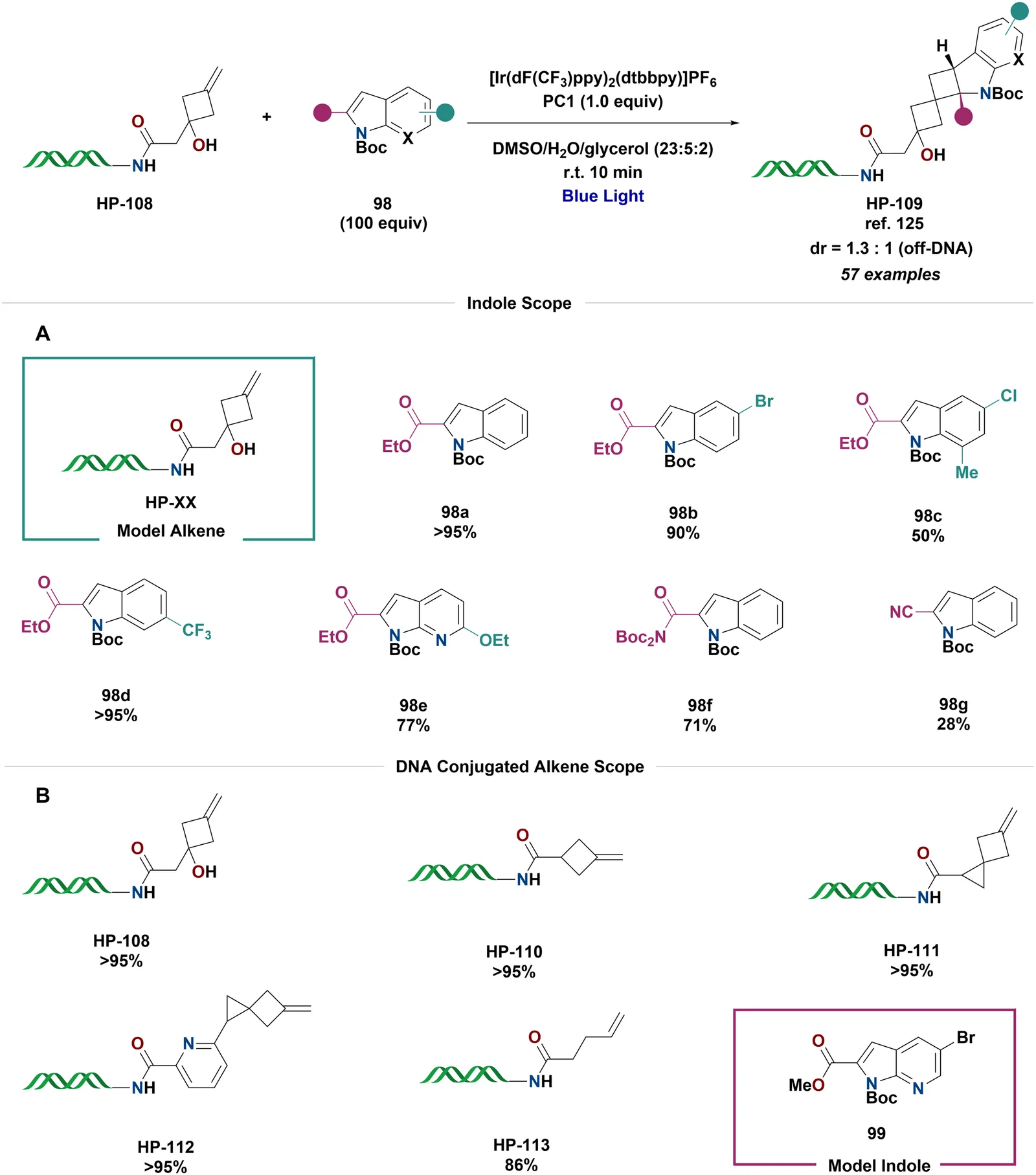
On-DNA [2 + 2] cycloaddition reaction between DNA-conjugated *exo*-methylenecyclobutanol HP-108 and indole 98 towards the synthesis of HP-109. (A) Scope of indoles compatible with the reaction with HP-108. (B) Scope of DNA conjugated alkenes tolerated with indole 99.

Building on these developments, Molander went on to subsequently develop an on-DNA aza-[2 + 2] cycloaddition utilising DNA-conjugated *exo*-methylenecyclobutane HP-110 and 2-isoxazoline-3-carboxylates 100 to form 2-oxa-1-azabicyclo[3.2.0]heptanes HP-114 (Scheme 45).^126^ The 2-isoxazoline-3-carboxylate derivative bearing a phenyl ring (100a) showed excellent reactivity of 95%, on par with that observed with indoles. However, the inclusion of a bromide (100b) or the electron withdrawing cyano substituent (100c) on the phenyl ring resulted in a decrease to a moderate yield.^126^ Despite this, these reactions are still sufficiently efficient for inclusion into DEL programmes, providing access to promising drug fragments. In contrast to the presence of halides and electron-withdrawing groups, an electron-donating group in this position reinstated the excellent yield (100d). This is the opposite effect to that seen with indoles whereby electron-withdrawing groups performed more favourably than electron-donating groups. Primary alkyl substitution of the 2-isoxazoline-3-carboxylate with a malonate group (100e) was similarly well tolerated as was the inclusion of a nitrogen containing spirocycle (100f).^126^ Together, these Molander reports provide direct on-DNA access to sp^3^-rich spirocyclic scaffolds, expanding DEL synthesis into regions of chemical space that are poorly served by classical on-DNA reactions.

**Scheme 45:**
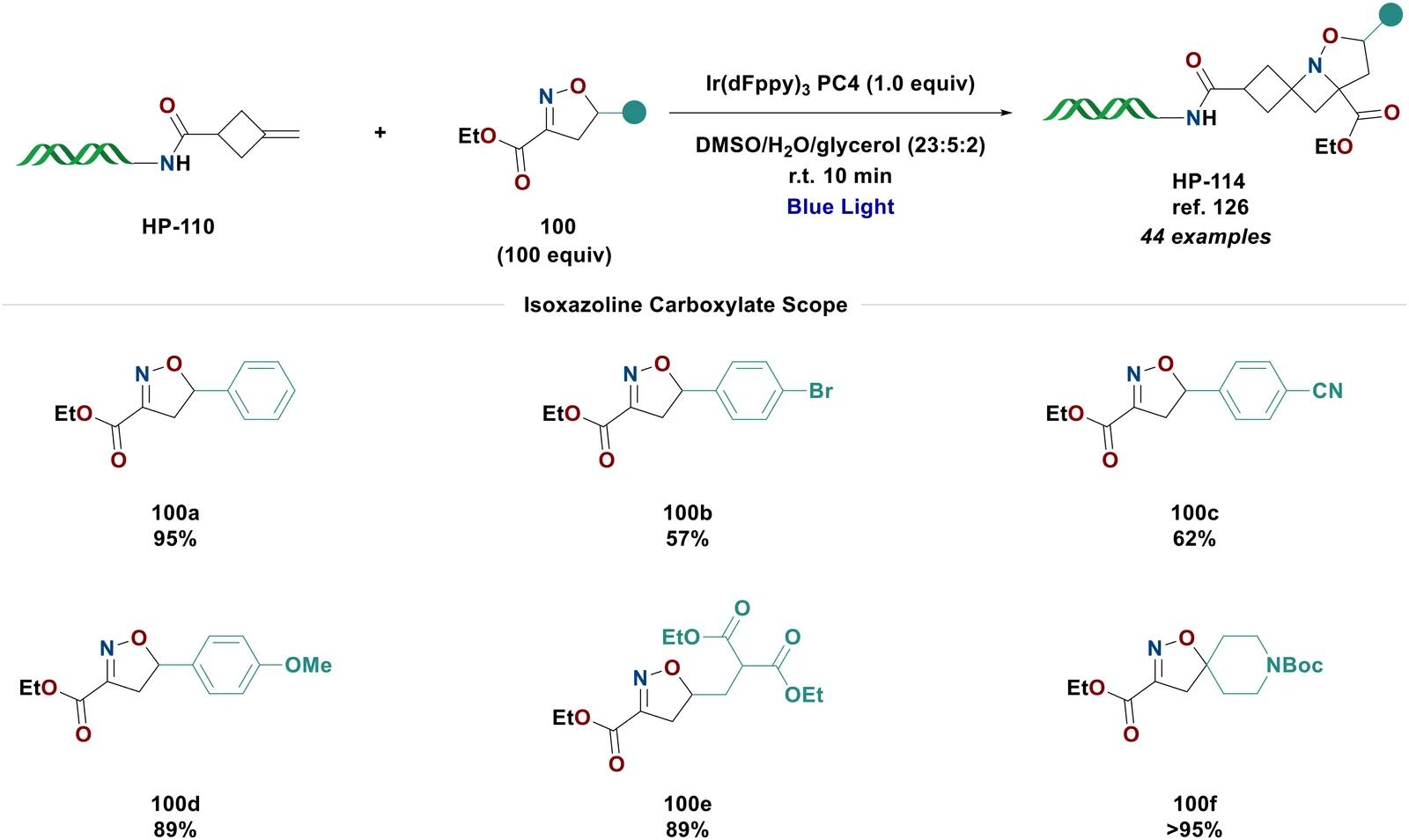
On-DNA aza-[2 + 2] cycloaddition reaction between DNA-conjugated *exo*-methylenecyclobutane HP-110 and 2-isoxazoline-3-carboxylates 100 towards the synthesis of HP-114 along with the scope of 2-isoxazoline-3-carboxylates compatible with the reaction.

### 3.6 Photolysis and direct excitation

Photolysis offers an alternative mode of photoactivation and radical generation. Irradiation promotes bond homolysis to generate radical intermediates, rather than proceeding through photocatalyst-mediated SET or energy transfer. This homolytic cleavage can be facilitated by the presence of a weak bond within the molecule or driven by the thermodynamically favourable release of a stable compound such as nitrogen gas. In other cases, direct photon absorption by photoactive functional groups such as nitro groups form reactive radical intermediates that promote bond cleavage and subsequent functionalisation.

Ritter has recently exploited photolysis to induce a range of photochemical transformations using a novel selenonium salt HP-116 (Scheme 46). In this case, photolysis is induced by breaking a weak C–Se bond in HP-116 to form an aryl radical. This reactivity is consistent with population of a C–Se σ* antibonding orbital and the relatively weak C–Se bond of the selenonium motif (71 kcal mol^−1^), which enables efficient photochemical fragmentation.^127^ The aryl-selenonium linchpin is installed directly on DNA by regioselective C–H functionalisation of electron-rich arenes such as HP-115 using an activated selenoxide reagent (101) under aqueous acidic conditions.^128^ Following photolysis, the resulting aryl radical can couple with a range of radical acceptors. One such example is the reaction with 4-methylbenzenethiol 102 to form a new C–S bond in biaryl sulfide HP-117. Here the aryl radical couples to the thiolate to form a radical anion species. This radical anion can then participate in electron transfer to propagate fragmentation of another aryl-selenonium salt, closing a radical-chain process. By adding thiophenols to DNA-conjugated arenes, this offers a complementary approach to the work of Hu, who used DNA-conjugated thiols.^120^ An analogous reaction was demonstrated between HP-116 and sodium iodide 103 whereby the aryl radical generated from photolysis can form a radical anion species with the iodide and then donate an electron to form the corresponding iodoarene HP-118.^128^ This is a particularly powerful transformation as the aryl iodide is a useful precursor for other on-DNA reactions such as Suzuki coupling or metallaphotoredox coupling and therefore allows for rapid library expansion.^40,107^ Previously, iodoarene precursors have been constructed off-DNA first before DNA conjugation, so to have a robust methodology to generate these from the parent arene on-DNA increases the capabilities of the DEL platform. Finally, selenonium salt HP-116 can also undergo a similar reaction with 3-methylisoquinoline 104 in an Minisci-type coupling to give HP-119.^128^ Here, following photolysis, the aryl radical can form a new C–C bond at the α-nitrogen position of the 3-methylisoquinoline 104 to form a radical cation. Deprotonation by 1,2,2,6,6-pentamethylpiperidine (PMP) allows for re-aromatisation and the formation of another radical species which can then donate an electron to form functionalised product HP-119. This highlights how the selenonium salt HP-116 is a powerful precursor for a range of photochemical transformations, particularly due to the facile nature of the C–Se bond and offers great potential for library diversification.^128^

**Scheme 46:**
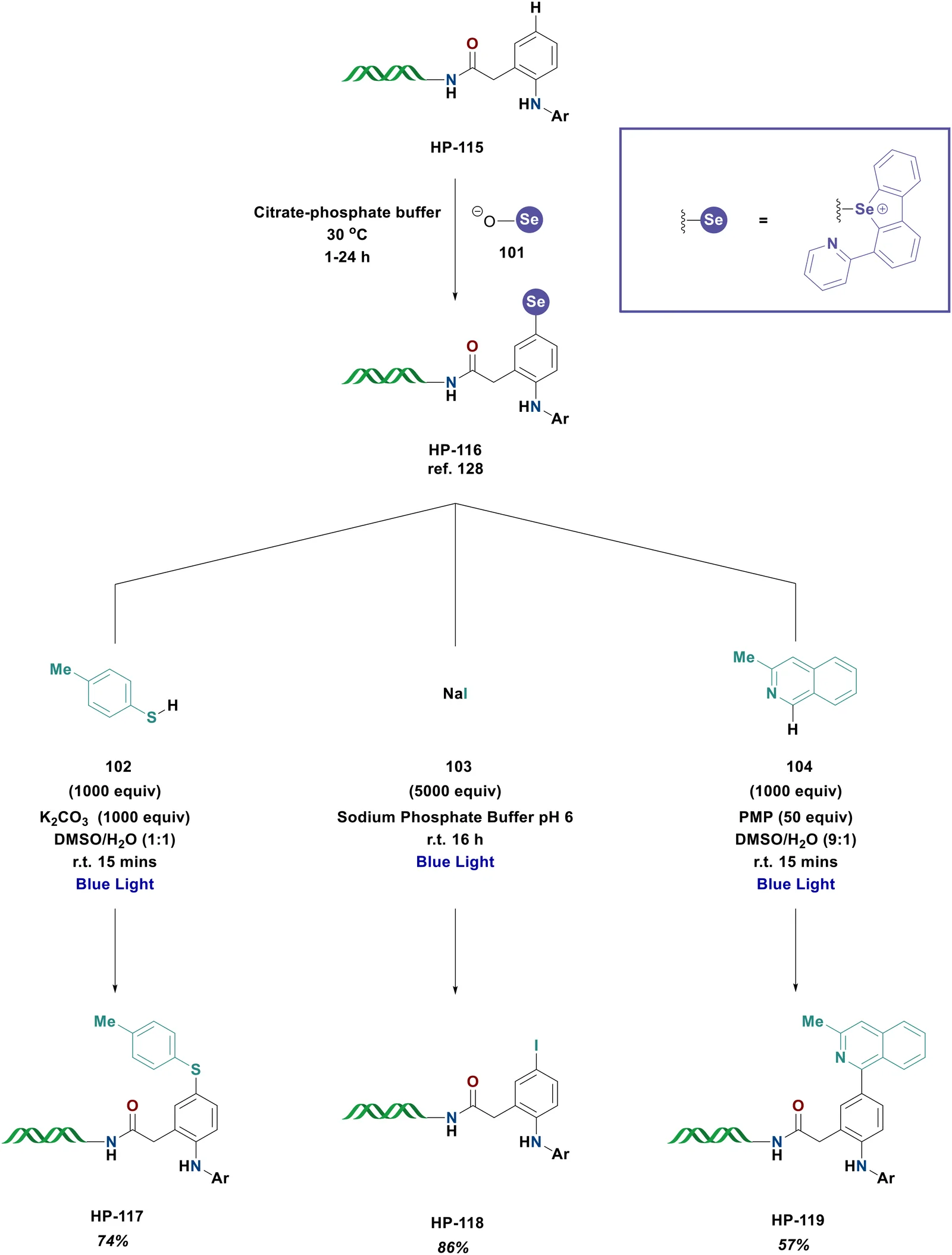
On-DNA photochemical transformations enabled by the photolysis of selenonium salt HP-116 which can be constructed by selective C–H activations of DNA-conjugated arene HP-115 with selenoxide 101. Following the formation of selenonium salt HP-116, photolysis can break the C–Se bond allowing for radical coupling with a thiophenol, iodide or nitrogen heterocycle.

Building on this, Ritter utilised photolysis to enable a separate on-DNA transformation, namely the ruthenium-mediated S_N_Ar reaction of DNA-conjugated amines (Scheme 47). In this process, the ruthenium catalyst 106 forms an η-6 arene (107) upon premixing with arene 105 and thus activates it towards the addition of amine HP-120 in an S_N_Ar process. However, the ruthenium remains coordinated in intermediate HP-121 and therefore photolysis is required to break down the complex and release the product HP-122. This highlights how photochemistry can be used as a reaction enabling tool as opposed to a direct activator or catalyst. On-DNA S_N_Ar processes were previously known with activated arenes,^33,34^ however, this methodology further expands the scope of on-DNA S_N_Ar to include unactivated arenes.^129^

**Scheme 47:**
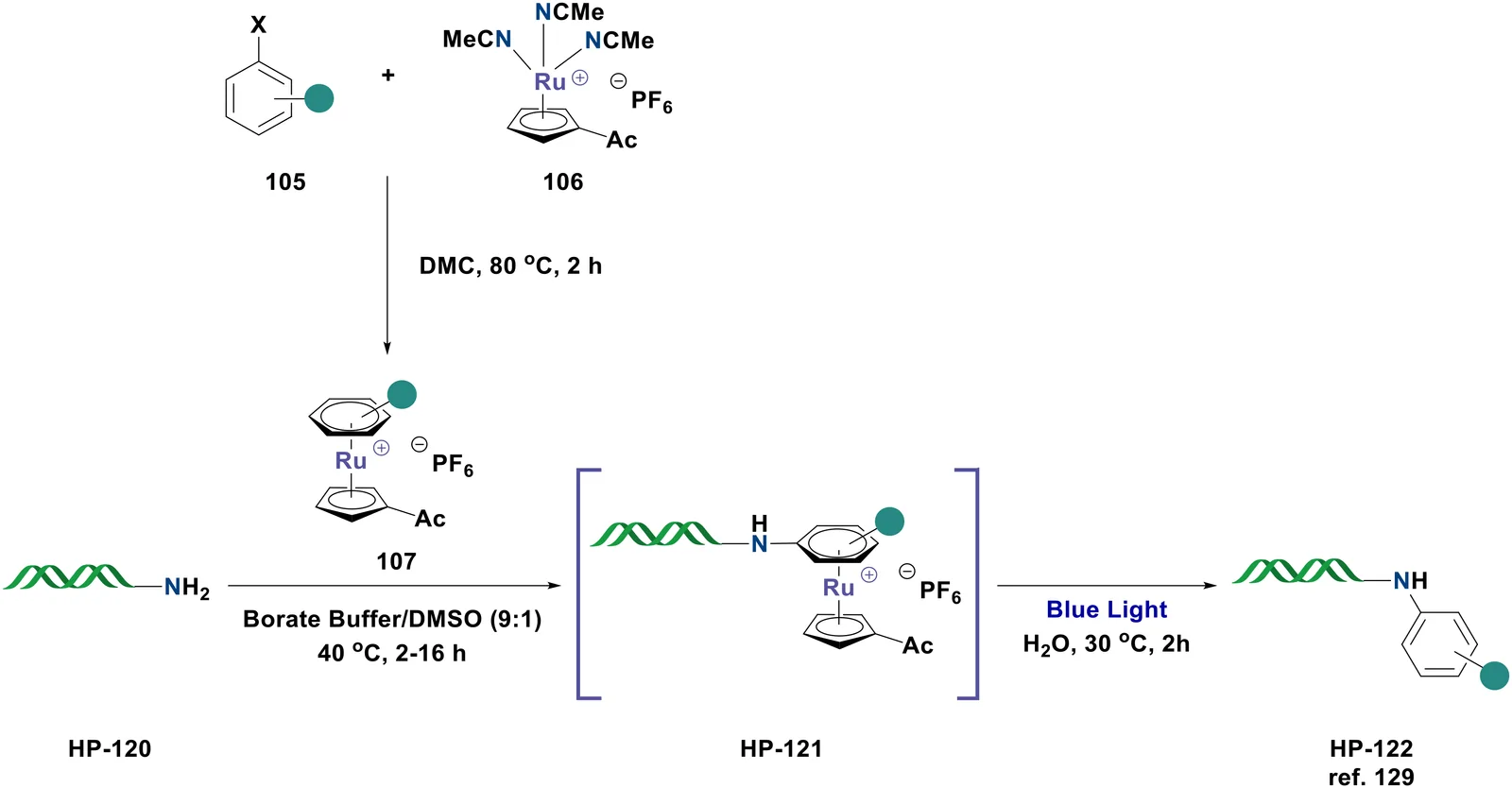
On-DNA synthesis of HP-122 enabled by the photolysis of the Ru catalyst. Aryl halide 105 and ruthenium catalyst 106 are premixed to form a complex that activates the aryl halide toward nucleophilic aromatic substitution (S_N_Ar) with DNA-tethered amine HP-120 to form intermediate HP-121. Irradiation with blue light breaks down the complex to release product HP-122.

Another example of an on-DNA process being enabled by photochemical homolysis can be seen in the work of Li whereby a S–S bond is broken in *p*-tolyl disulfide 108 to generate a sulfur-centred radical.^130^ This radical can then abstract a hydrogen from the heterocycle formed by the polar addition of DNA-conjugated aldehyde HP-123 and anilines 109–112, to facilitate aromatisation to products HP-124–HP-127 (Scheme 48). A range of different heterocycles were constructed using this method including benzimidazoles HP-124, benzothiazoles HP-125, quinazolinones HP-126 and quinazolines HP-127.^130^

**Scheme 48:**
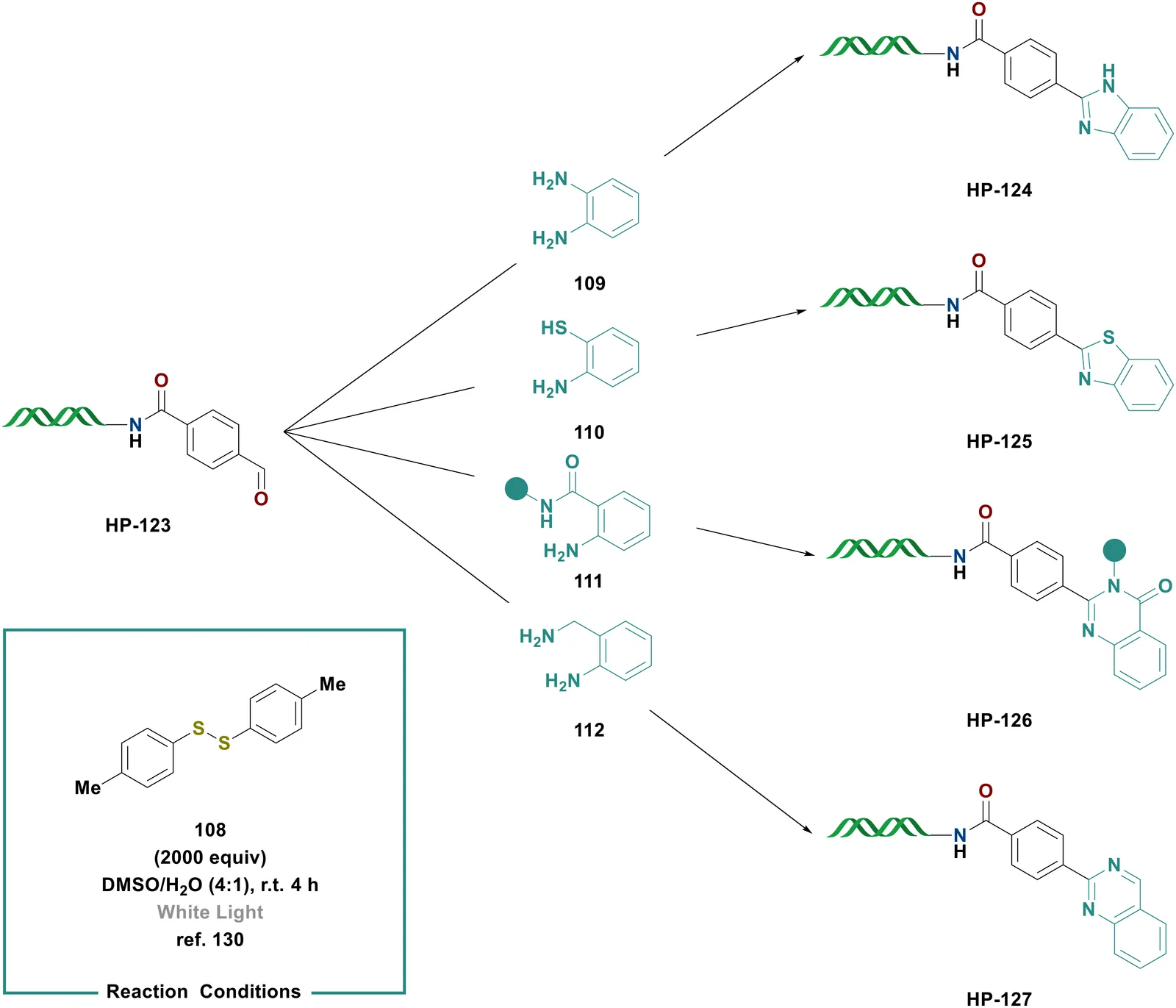
On-DNA synthesis of DNA-conjugated heterocycles HP-124–HP-127 initiated by the photolysis of 108. Amine and thiol addition to DNA-conjugated aldehyde HP-123 forms dihydroheterocycles which can be aromatised by hydrogen-atom abstraction from thiolate radicals from the photolysis of 108.

Lu also reported a photochemical method towards heterocycle synthesis with an on-DNA primary amine and *o*-nitrobenzyl alcohol cyclization (PANAC) photoclick reaction (Scheme 49).^62^ Here, photochemical excitation of the nitro group in HP-128 at 365 nm facilitates an intramolecular rearrangement resulting in the loss of water to form nitroso intermediate HP-129.^131,132^ This can react with primary amine 113 to form indazolone HP-130.^62,131,132^ Lu used this methodology to construct a library of >100 000 compounds which provided several promising hits against E1A-binding protein p300.^62^

**Scheme 49:**
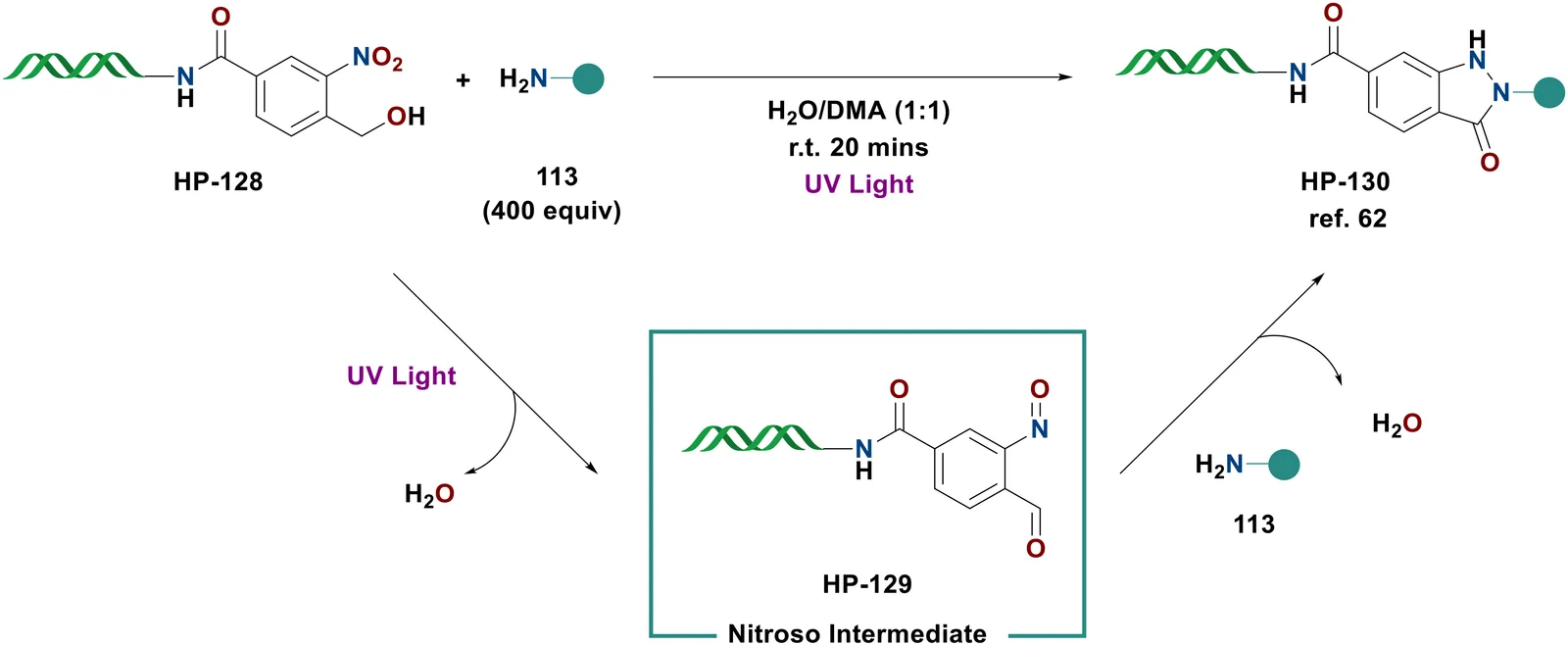
On-DNA synthesis of DNA-conjugated indazolone HP-130. Excitation of the nitro group in HP-128 allows for radical rearrangement and the elimination of H_2_O to form nitroso intermediate HP-129 which can capture amine 113 to form HP-130.

Photolysis can also be driven by the release of nitrogen gas in the case of a C–N or N–N bond homolysis. Zhang reported a DNA-compatible amination reaction facilitated by a DNA-conjugated diazo compound HP-131 (Scheme 50).^133^ Upon irradiation this can release nitrogen gas to form a carbene intermediate that can couple with amines (114) to form products of type HP-132. Aliphatic amines (114a–c) such as betahistine 114b were seen to tolerate the reaction, as were amino acids 114c, nitrogen heterocycles (114d) and aromatic amines (114e–f), including those bearing electron-withdrawing groups such as 114f.^133^ This methodology also utilised PAX resin in a RASS protocol to carry out the reaction under anhydrous conditions.^133^

**Scheme 50:**
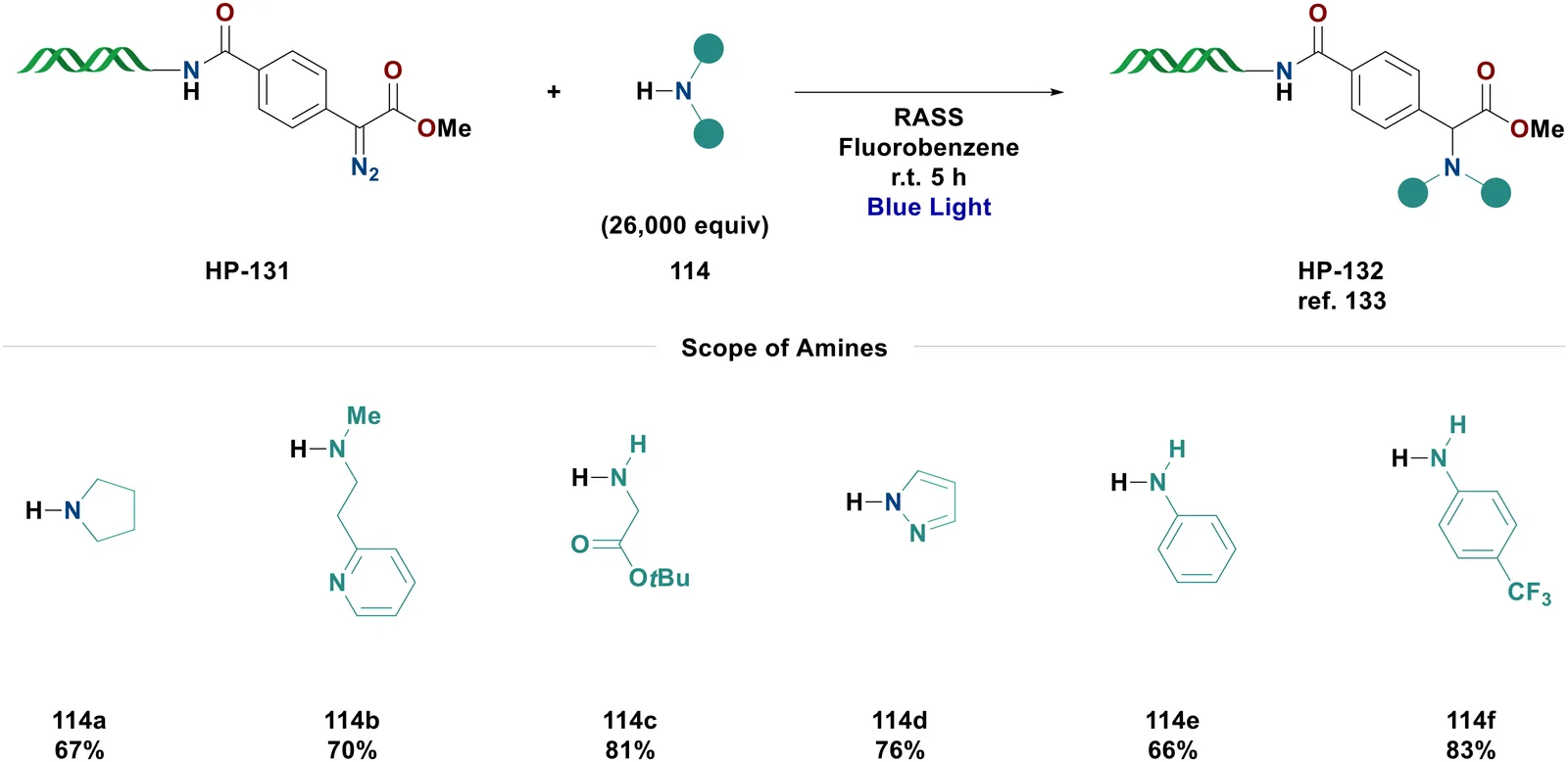
On-DNA reaction between DNA-conjugated diazo substrate HP-131 and amines 114 towards the synthesis of HP-132 along with the scope of amines compatible with the reaction. Photolysis of HP-131 forms a carbene which can couple to amine 114. Reaction carried out under inert conditions using RASS.

In a similar approach, the Li research group also used photolysis to stimulate the release of nitrogen gas and generate a useful reactive intermediate. Rather than diazo compounds, tetrazole substrates (HP-133) are used here which upon the loss of nitrogen forms a nitrile imine intermediate HP-134 which can go on to capture a range of coupling partners (115–117).^134–136^ In the case of alkene 115 and amine 116, a cycloaddition reaction occurs to form pyrazoline HP-135 and triazole HP-136 respectively (Scheme 51).^134,136^ Meanwhile in the case of carboxylic acid 117, diacylhydrazine HP-137 was formed.^135^ Here, the carboxylic acid reacts with the nitrile in HP-134 before undergoing an acyl shift to afford HP-137.^135,137^ Interestingly, in each case, UV radiation at 302 nm was used to induce photolysis without inducing significant DNA damage. This is potentially due to the reactive nature of the tetrazole ring, in that only 10 minutes of irradiation was necessary to induce photolytic cleavage therefore not exposing the DNA to enough radiation to cause damage.

**Scheme 51:**
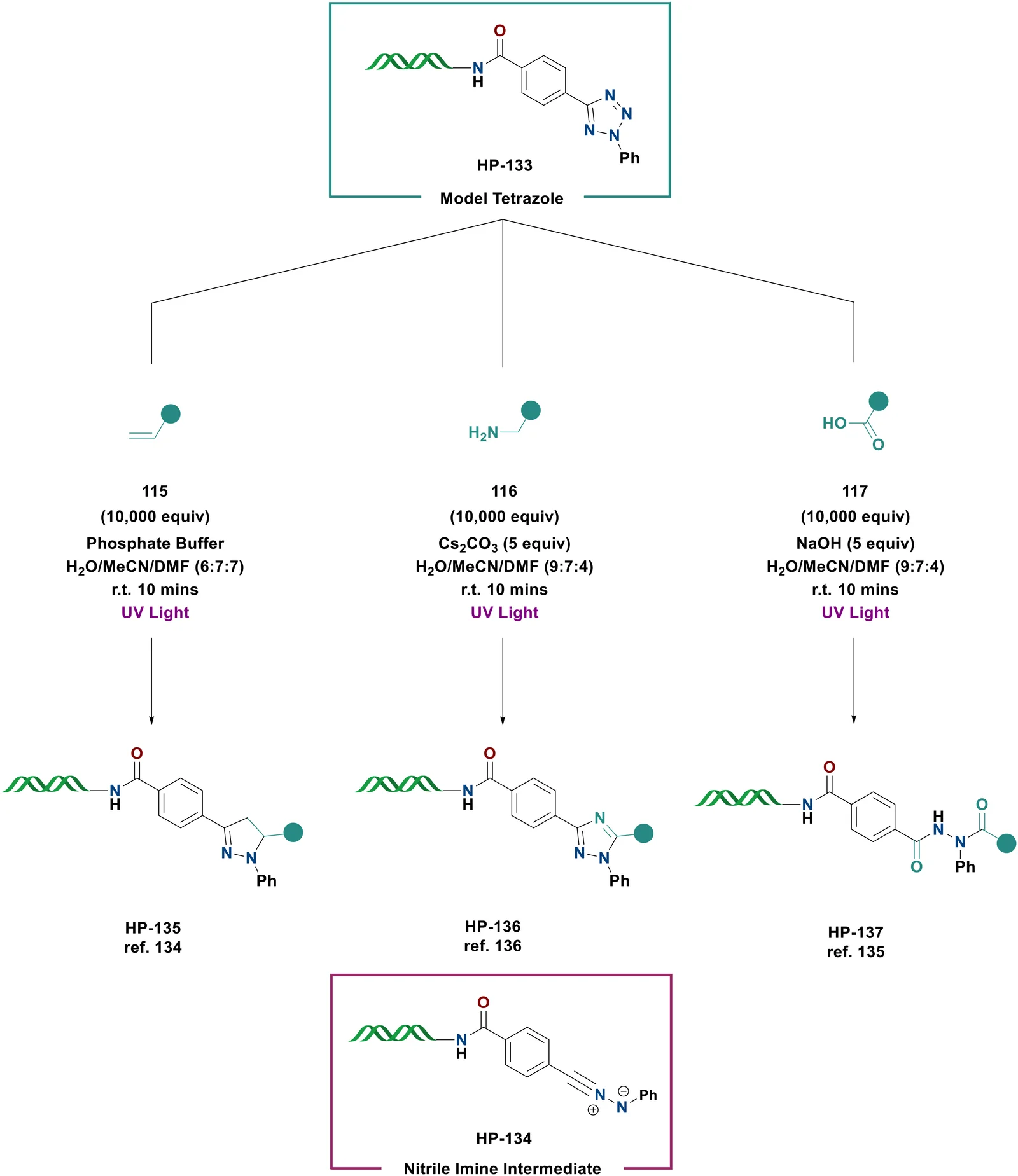
On-DNA synthesis of HP-135–HP-137 enabled by the photolysis of tetrazole HP-133. Upon irradiation with UV light, HP-133 releases nitrogen to form intermediate HP-134 which can undergo functionalisation *in situ*. Coupling with alkene 115 affords HP-135. Coupling with amine 116 affords HP-136. Coupling with carboxylic acid 117 affords HP-137.

Finally, DNA conjugated azides such as HP-138 can also undergo photolysis to release nitrogen. Here, a nitrene rather than a carbene is formed.^138^ This singlet nitrene can react with the phenyl ring to form a bicyclic azirine which can break down into a ketimine which can subsequently undergo nucleophilic attack with amine 118 to form a seven-membered 3H-azepine HP-139 (Scheme 52).^138,139^ This methodology presented by Zhang is the first account of an on-DNA, photochemical skeletal editing reaction and 3H-azepine HP-139 presents a useful synthetic handle for further diversification and skeletal modification.^138,139^ Once again, a RASS protocol is utilised with the DNA being tethered to a PAX resin to allow for the use of DMA as the neat organic solvent. Following on from this, Zhang devised an alternative photochemical route to HP-139 starting from DNA-conjugated nitrobenzene HP-140. Here, the addition of P(OiPr)_3_ was necessary to act as a reducing agent to facilitate the removal of oxygen.^139,140^ The use of a RASS system here was imperative in preventing any unwanted interaction between the P(OiPr)_3_ and the DNA backbone.^140^

**Scheme 52:**
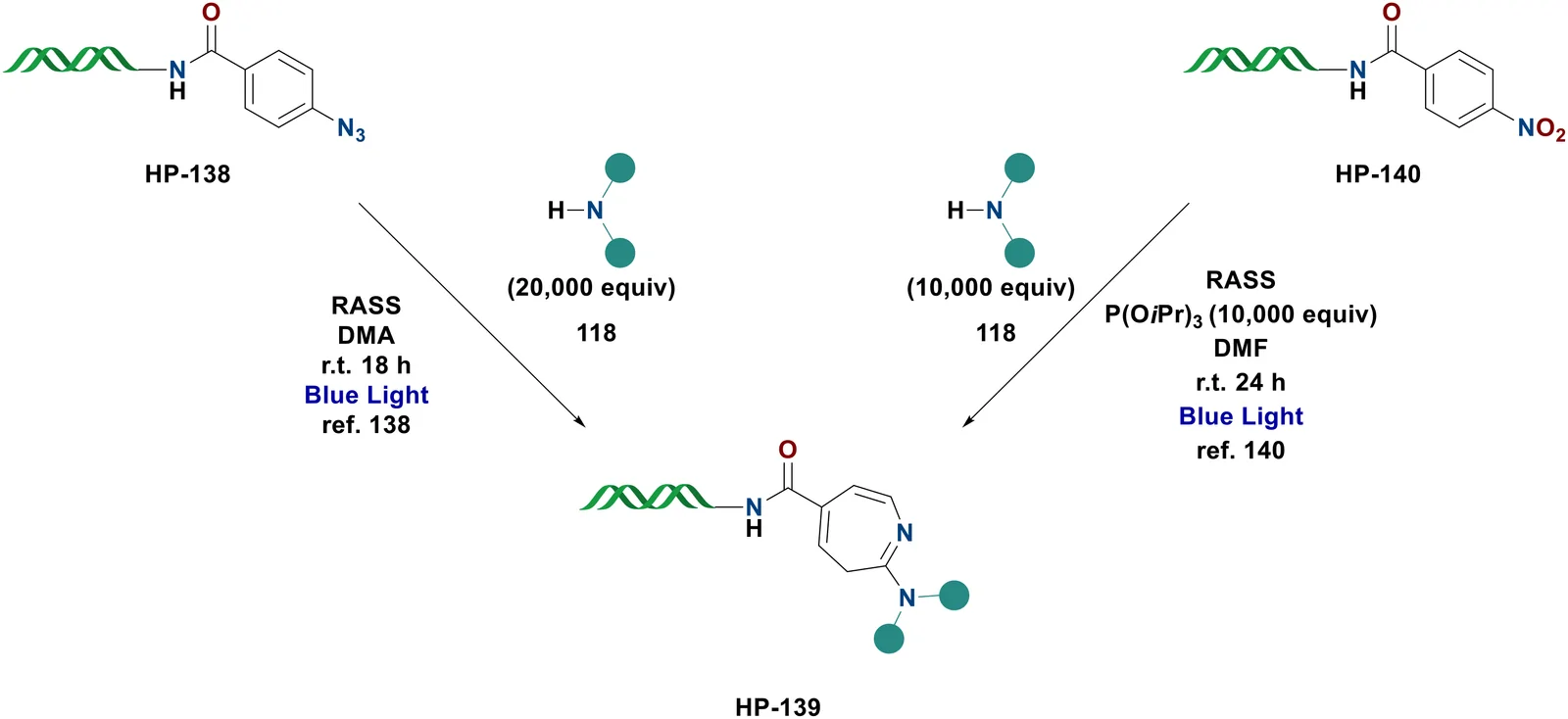
On-DNA skeletal editing of DNA-conjugated azide HP-138 or DNA conjugated nitroarene HP-140 to construct seven-membered ring HP-139 with amine 118. Reactions carried out under inert conditions using RASS.

## 4. Future directions

Despite the rapid growth in the number of on-DNA photochemical transformations, this field is still in its infancy. The development and adaptation of novel on-DNA photochemical reactions remain a key tool towards building structurally complex DELs that cover untapped regions of chemical space. In particular, EnT catalysed reactions are still relatively unexplored in the on-DNA landscape compared to SET processes and more work could be done to adapt these useful processes that build molecular complexity. In addition, the field of on-DNA photocatalysis is dominated by blue light reactions along with several examples of UV or white light reactions. In the off-DNA literature there is an extensive catalogue of red-light-activated processes which have yet to be adapted towards on-DNA synthesis.^141,142^ These lower energy wavelengths (*e.g.* red light) could be beneficial in inducing less DNA damage and could open the door to unexplored classes of photocatalyst. Furthermore, the field of biocatalysis has yet to be explored in the context of on-DNA photochemistry. Biocatalysis has been used to facilitate several simple on-DNA transformations including amide coupling,^143^ and aldol reactions,^144,145^ however it remains an underutilised tool in on-DNA synthesis so far. Since these enzymes also require an aqueous environment to operate, they are highly amenable to the typical on-DNA workflow. In the context of on-DNA photochemistry, an engineered photoenzyme containing a chromophore could be used to directly carry out a desired transformation.^146^ Conversely, a dual-catalytic approach could be used whereby a traditional PC such as those described in this review could be coupled to a redox enzyme which can participate in a dual catalytic cycle.^147,148^

Looking downstream of the DEL workflow, it is worth noting that there are few cases where on-DNA photochemistry has been used in the construction of a DEL for compound screening. Several proof-of-concept libraries have been constructed but screening of these against any biological target is not yet reported in the public domain.^69,134^ Lu's photochemical, on-DNA indazolone synthesis was used in the construction of a >100 000 compound library that was screened against p300, however further examples of photochemically derived DELs remain limited.^62^ One possible reason for this is the drop-off in reaction yield when longer DNA tags are used. For example, in Molander's report of an on-DNA Giese reaction with BCP iodides, the yield dropped from 91% to 39% when a longer tag was used.^61^ This limitation has started to be addressed by the DEL community. For example, Jeon reported an on-DNA nitro reduction reaction optimised to a longer DNA tag of 44 base pairs rather than the 6 base pair tag typically used.^149^ Similar studies with these on-DNA photochemical reactions would be equally valuable and would strengthen the case for using photochemically built DELs in compound screening.

Finally, with rapid advances in the fields of machine learning and chemputation,^150^ these tools could be used to efficiently adapt and optimise novel on-DNA photochemical transformations. High-throughput automated experimentation can generate large, internally consistent datasets that can be used to train machine-learning models for reaction optimisation and prediction.^151,152^ These advances are already making their way into the DEL space with open-source packages such as DELi, which helps users decode libraries, design barcodes and enumerate potential libraries.^153^ Off-DNA photochemistry is already benefitting from creative uses of machine learning such as the discovery of new organo-photocatalysts reported by Glorius.^154^ As these tools continue to improve, it is likely that the design, execution and optimisation of on-DNA photochemical reactions will become increasingly data-driven, lowering the barrier to incorporating photochemistry into mainstream DEL production.

## 5. Conclusions

DNA-encoded libraries have become a powerful platform for early-stage hit discovery because they allow millions to billions of compounds to be screened in a single pooled experiment. However, the quality of a DEL is defined not only by its numerical size, but by the chemistry used to construct it. Classical on-DNA reactions such as amide coupling, reductive amination, S_N_Ar chemistry and transition-metal cross-coupling have enabled reliable library synthesis, but they often favour relatively planar, sp^2^-rich products. One core challenge for the field is, therefore, to build molecular shape, stereochemical information and sp^3^-rich complexity within the two or three encoded synthetic cycles commonly used in DEL construction.

Photochemistry is emerging as a powerful solution to this problem. Excited-state activation can access reaction pathways that are difficult to achieve using conventional ground-state chemistry, often under mild and DNA-compatible conditions. Photoredox single-electron transfer has enabled on-DNA Giese reactions, *gem*-difluoroalkene synthesis, radical cyclopropanation, carbonyl coupling, atom-transfer radical addition and Minisci-type heteroarene functionalisation. These methods have translated diverse radical precursors, including carboxylic acids, amino acids, xanthates, imines, sulfinates, silicates, diazo compounds, redox-active esters and alkyl halides, into encoded molecular diversity.

Beyond classical photoredox chemistry, complementary light-driven activation modes are now expanding what can be achieved on-DNA. Hydrogen-atom transfer allows C–H bonds adjacent to nitrogen to act as radical precursors without pre-functionalisation. Metallaphotoredox catalysis has enabled C(sp^2^)–C(sp^3^) cross-coupling between DNA-conjugated aryl halides and alkyl radical precursors. Electron donor–acceptor complexes provide photocatalyst-free routes to radical generation, while energy-transfer catalysis has introduced [2 + 2] cycloaddition chemistry to DEL synthesis. Direct photolysis has further enabled aryl radical, carbene, nitrile imine and nitrene chemistry from photolabile DNA-conjugated precursors. The medicinal chemistry value of these methods is high. On-DNA photochemistry has enabled access to cyclobutanes, bicyclo[1.1.1]pentanes, spirocycles, *gem*-difluoroalkenes, azabicyclic structures, C–S linked products and heteroaryl-functionalised scaffolds. These motifs offer routes into more three-dimensional, saturated and bioisosteric chemical space that is poorly served by many classical DEL reactions.

The DEL community should now combine continued reaction discovery with library implementation. New photochemical transformations are still needed to access scaffold classes, bond disconnections and three-dimensional structures that remain unexplored using current on-DNA chemistry. However, the real test will be whether these reactions can be translated into encoded libraries that can be synthesised, selected and sequenced. In practical terms, the value of on-DNA photochemistry will be measured by the diversity and quality of the libraries it enables. If successful, these methods should provide DELs with greater shape, complexity and medicinal relevance, leading to more diverse hits and potentially stronger starting points for drug discovery.

## Author contributions

Oliver J. Corrigan: conceptualisation, investigation, writing – original draft. Sarah L. Allinson: supervision, writing – review & editing. Jamie H. Docherty: conceptualisation, supervision, writing – review & editing.

## Conflicts of interest

There are no conflicts to declare.

## Data Availability

Data sharing is not applicable to this article as no datasets were generated or analysed during the current study.
